# Supplementary material for: Telling the same story: Fishers and landing data reveal changes in fisheries on the Southeastern Brazilian Coast
Source: PLoS One. 2021 Jun 1;16(6):e0252391. doi: 10.1371/journal.pone.0252391 (PMC8168859; doi:10.1371/journal.pone.0252391)
Supplement: S1 Data — (DOCX) [file pone.0252391.s001.docx]

**S1 Data**

**S1 Table. Trophic level (TL) of species landed Arraial do Cabo, from 1992 to 2008.** Trophic levels were obtained from FishBase where a higher value means higher trophic levels (Froese and Pauly, 2019).

| **LANDED SPECIES - FIPAC** | | | |
| --- | --- | --- | --- |
| **Common name** | **Scientific name** | **Trophic Level** | **Taxonomic group** |
| Brazilian sardinella | *Sardinella brasiliensis* | 3.1^a^ | Teleosts |
| Bluefish | *Pomatomus saltatrix* | 4.5^a^ |  |
| Largehead hairtail | *Trichiurus lepturus* | 4.4^a^ |  |
| Little tunny | *Euthynnus alletteratus* | 4.5^a^ |  |
| Chub mackerel | *Scomber japonicus* | 3.4^a^ |  |
| Horse-eye jack | *Caranx latus* | 4.2^a^ |  |
| Common dolphinfish | *Coryphaena hippurus* | 4.4^a^ |  |
| Atlantic moonfish | *Selene setapinnis* | 3.7^a^ |  |
| Namorado sandperch | *Pseudopercis numida* | 3.9^a^ |  |
| Atlantic thread herring | *Opisthonema oglinum* | 4.5^a^ |  |
| Atlantic bonito | *Sarda sarda* | 4.5^a^ |  |
| White mullet | *Mugil curema* | 2^a^ |  |
| Lebranche mullet | *Mugil liza* | 2^a^ |  |
| Atlantic bumper | *Chloroscombrus chrysurus* | 3.5^a^ |  |
| Jack Crevalle | *Caranx hippos* | 3.6^a^ |  |
| Lesser amberjack | *Seriola fasciata* | 4.5^a^ |  |
| Frigate tuna | *Auxis thazard* | 4.4^a^ |  |
| Argentine conger | *Conger orbignyanus* | 3.7^a^ |  |
| Silver porgy | *Diplodus argenteus* | 3.1^a^ |  |
| Unicorn leatherjacket filefish | *Aluterus monoceros* | 3.8^a^ |  |
| Dusky grouper | *Epinephelus marginatus* | 4.4^a^ |  |
| Toadfish | *Porichthys porosissimus* | 3.7^a^ |  |
| Yellowtail amberjack | *Seriola lalandi* | 4.2^a^ |  |
| Atlantic sailfish | *Istiophorus albicans* | 4.5^a^ |  |
| Whitemouth croaker | *Micropogonias furnieri* | 3.1^a^ |  |
| Red porgy | *Pagrus pagrus* | 3.9^a^ |  |
| Tile fish | *Lopholatilus villarii* | 3.8^a^ |  |
| Snowy grouper | *Epinephelus niveatus* | 4^a^ |  |
| Atlantic bluefin tuna | *Thunnus thynnus* | 4.5^a^ |  |
| King weakfish | *Macrodon ancylodon* | 3.9^a^ |  |
| Striped weakfish | *Cynoscion striatus* | 3.9^a^ |  |
| Skipjack tuna | *Katsuwonus pelamis* | 4.4^a^ |  |
| Smallscale weakfish | *Cynoscion microlepidotus* | 4^a^ |  |
| Atlantic bigeye | *Priacanthus arenatus* | 4^a^ |  |
| Common halfbeak | *Hyporhamphus unifasciatus* | 2^a^ |  |
| Sand drum | *Umbrina coroides* | 3.1^a^ |  |
| Yellowfin tuna | *Thunnus albacares* | 4.4^a^ |  |
| Brazilian flathead | *Percophis brasiliensis* | 4.2^a^ |  |
| Grey triggerfish | *Balistes capriscus* | 4.1^a^ |  |
| Argentine goatfish | *Mullus argentinae* | 3.5^a^ |  |
| Albacore | *Thunnus alalunga* | 4.3^a^ |  |
| King mackerel | *Scomberomorus cavalla* | 4.4^a^ |  |
| Brazilian codling | *Urophycis brasiliensis* | 3.9^a^ |  |
| Ladyfish | *Elops saurus* | 3.5^a^ |  |
| Florida pompano | *Trachinotus carolinus* | 3.5^a^ |  |
| Atlantic spadefish | *Chaetodipterus faber* | 4.5^a^ |  |
| Brazilian flounder | *Paralichthys brasiliensis* | 4.4^a^ |  |
| Mackerel scad | *Decapterus macarellus* | 4^a^ |  |
| Argentine hake | *Merluccius hubbsi* | 4.2^a^ |  |
| American harvestfish | *Peprilus paru* | 4.5^a^ |  |
| Gag | *Mycteroperca microlepis* | 3.7^a^ |  |
| Gafftopsail sea catfish | *Bagre marinus* | 3.5^a^ |  |
| Southern red snapper | *Lutjanus purpureus* | 3.6^a^ |  |
| Bigtooth corvina | *Isopisthus parvipinnis* | 4^a^ |  |
| Corocoro grunt | *Orthopristis ruber* | 3.6^a^ |  |
| Bermuda sea chub | *Kyphosus sectatrix* | 2^a^ |  |
| Atlantic goliath grouper | *Epinephelus itajara* | 4.1^a^ |  |
| Ballyhoo halfbeak | *Hemiramphus brasiliensis* | 2.3^a^ |  |
| Bluewing searobin | *Prionotus punctatus* | 3.8^a^ |  |
| Maracaibo leatherjacket | *Oligoplites palometa* | 4.3^a^ |  |
| Brazilian mojarra | *Eugerres brasilianus* | 3.4^a^ |  |
| Jenny mojarra | *Eucinostomus gula* | 2.7^a^ |  |
| Common snook | *Centropomus undecimalis* | 4.2^a^ |  |
| Barred grunt | *Conodon nobilis* | 3.6^a^ |  |
| Broadband anchovy | *Anchoviella lepidentostole* | 3.1^a^ |  |
|  |  |  |  |
| Spinner shark | *Carcharhinus brevipinna* | 4.2^a^ | Elasmobranchs |
| Oceanic whitetip shark | *Carcharhinus longimanus* | 4.2^a^ |  |
| Spiny butterfly ray | *Gymnura altavela* | 4.5^a^ |  |
| Shortfin mako | *Isurus oxyrinchus* | 4.5^a^ |  |
| Argentine angelshark | *Squatina argentina* | 4.1^a^ |  |
| Smooth hammerhead | *Sphyrna zygaena* | 4.9^a^ |  |
| Chola guitarfish | *Rhinobatos percellens* | 3.6^a^ |  |
| Sandbar shark | *Carcharhinus plumbeus* | 4.5^a^ |  |
| Dusky smooth-hound | *Mustelus canis* | 3.6^a^ |  |
| Tiger shark | *Galeocerdo cuvier* | 4.5^a^ |  |
| Bonnethead | *Sphyrna tiburo* | 3.9^a^ |  |
| Blacktip shark | *Carcharhinus limbatus* | 4.4^a^ |  |
|  |  |  |  |
| Squid | *Doryteuthis plei* | 3,9 | Mollusks and crustaceans |
| Common octopus | *Octopus vulgaris* | 3.5^b^ |  |
| South American rock mussel | *Perna perna* | 2^b^ |  |
| Lobster | *Panulirus sp.* | 2,6 |  |

^a^ TLs were derived from FishBase.

^b^ TLs were derived from SeaLifeBase

**S2 Table. Parameters of the Poisson Generalized linear model for the number of species cited by fishers based on years of practice.**

| **Overexploited species** | | **Estimate** | | **Std. Error** | | **Z-value** | | **P- value** | | **AIC** | |
| --- | --- | --- | --- | --- | --- | --- | --- | --- | --- | --- | --- |
| (Intercept) | | 0.584 | | 0.134 | | 4.357 | | 1.32e-05 | |  | |
| Years of practice | | 0.009 | | 0.003 | | 2.616 | | 0.008 | | 519.34 | |
|  | |  | |  | |  | |  | |  | |
| **New target species** | | **Estimate** | | **Std. Error** | | **Z-value** | | **P- value** | | **AIC** | |
| (Intercept) | | 0.424 | | 0.145 | | 2.924 | | 0.003 | |  | |
| Years of practice | | 0.01 | | 0.004 | | 2.451 | | 0.0142 | | 509.02 | |
|  | |  | |  | |  | |  | |  | |
| AIC = Akaike information criterion | | | |  | |  | |  | |  | |

**S3 Table. Fishing effort of the artisanal fishing fleet of Arraial do Cabo, Rio de Janeiro, Brazil.**

| **Year** | **Number of boats** | | **Fishing days** | **Reference** |
| --- | --- | --- | --- | --- |
| 1993 | | - | 2.169 | FIPAC |
| 1994 | | - | 2.604 | FIPAC |
| 1995 | | 345 | 1.814 | FIPAC |
| 2002 | | 300 | - | Pinto da Silva (2002) |
| 2004 | | 217 | - | Caldasso et al. (2012) |
| 2017 | | 159 | - | FIPERJ (2017) |
| 2018 (Jan-Jun) | | 181 | 3.640 | PMAP-BS (2018) |
| 2018 (Jul-Dec) | | 146 | 2.206 | PMAP-BS (2018) |
| 2019 | | 171 | 4.310 | PMAP-BS (2019) |


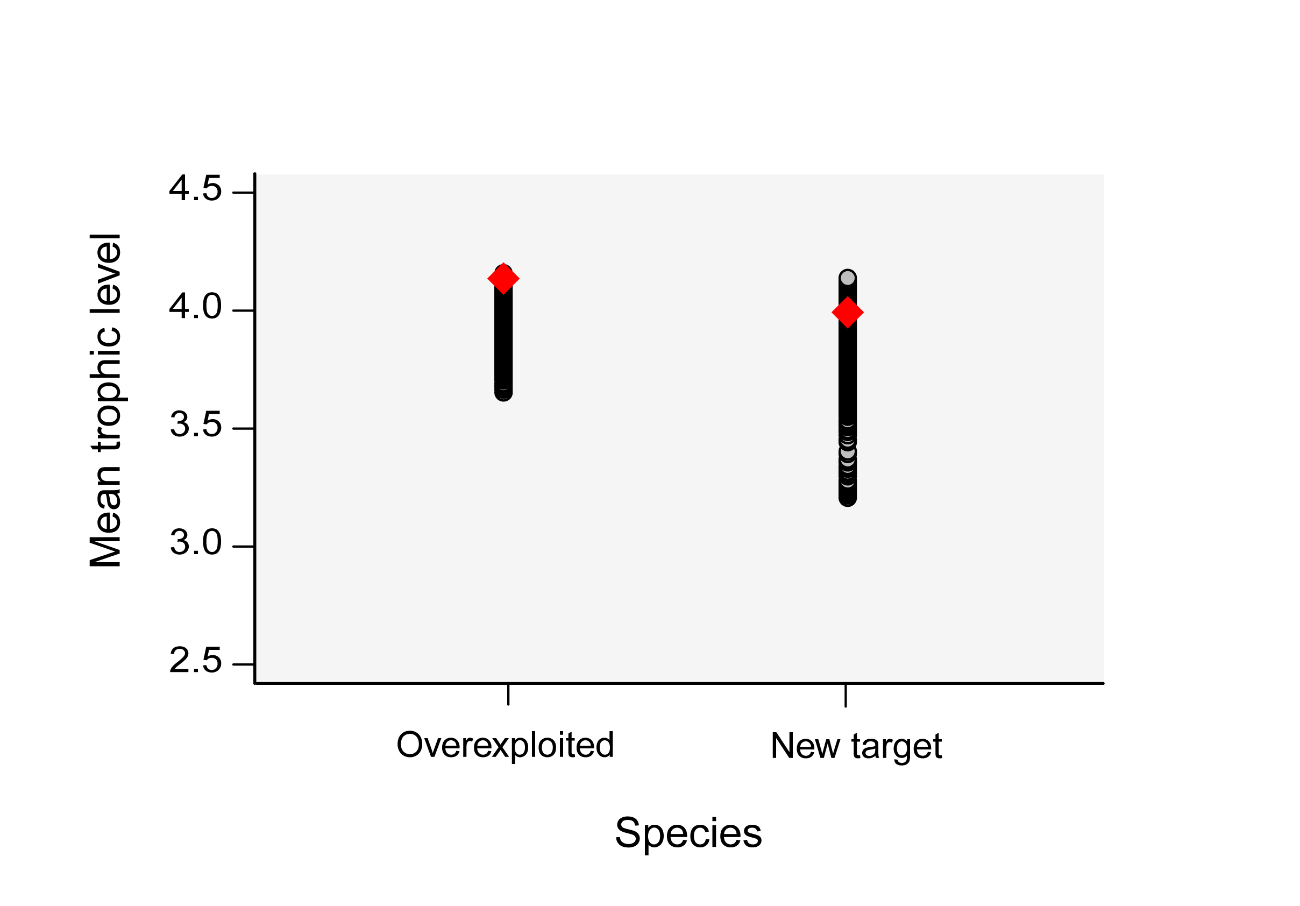


**S1 Fig. Differences in mean trophic level (MTL) of overexploited and new target species cited by local fishers.** Observed MTL (red diamond) are contrasted against null MTL values (gray circles), generated through randomization of citation frequency of species within each category.

**
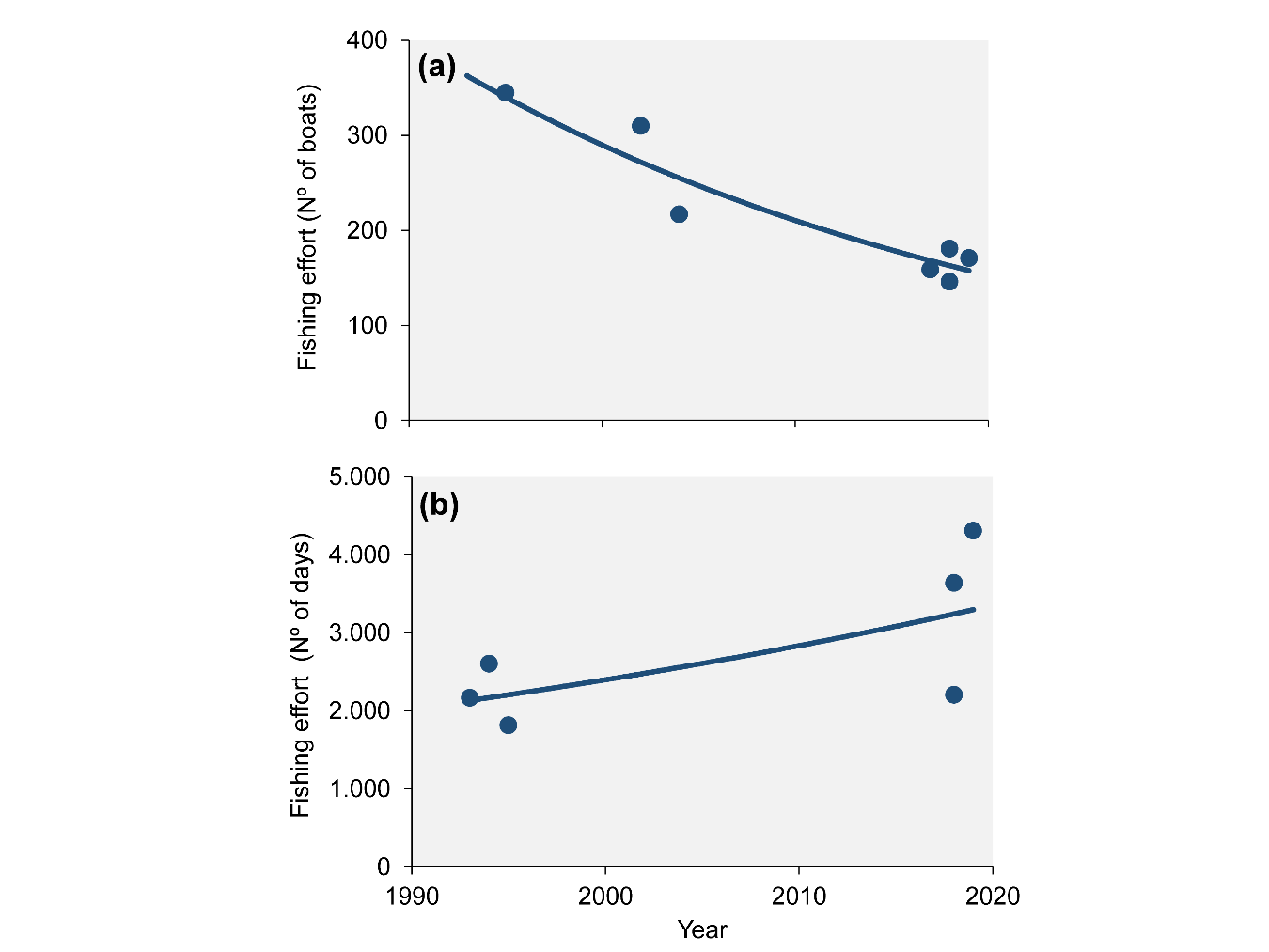
**

**S2 Fig. Fishing effort in number of boats (a) and fishing days (b) over the years for Arraial do Cabo, Rio de Janeiro, Brazil.**

**References**

Caldasso, L.P., Valle, R.,Vinha, V., 2012. Governança em Reserva Extrativista Marinha. First ed. PoD, Rio de Janeiro, 188p.

FIPAC, (Fundação Instituto de Pesca de Arraial do Cabo), 1992-1995. Acompanhamento e Avaliação da Atividade Pesqueira Municipal.

FIPERJ, (Fundação Instituto de Pesca do Rio Janeiro), 2017. Relatório Anual 2017. 108p.

Froese, R., Pauly, D. Editors., 2019. FishBase. World Wide Web electronic publication.www.fishbase.org, version (08/2019).

Pinto da Silva, P.S.V., 2002. Common property to co-management: Social change and participation in Brazil’s first Maritime Extractive Reserve. Thesis (phd). London School of Economics and Political Science (United Kingdom).

PMAP-BS, 2018. Projeto de Monitoramento da Atividade Pesqueira na Bacia de Santos- Relatório Técnico Semestral- janeiro a junho de 2018.

PMAP-BS, 2018. Projeto de Monitoramento da Atividade Pesqueira na Bacia de Santos- Relatório Técnico Semestral- julho a dezembro de 2018.

PMAP-BS, 2019. Projeto de Monitoramento da Atividade Pesqueira na Bacia de Santos- Relatório Técnico Semestral- janeiro a junho de 2019.

**S1 File.** **Questionnaire applied in interviews with fishers in Arraial do Cabo.**

Date: ___/___/_____ Interview number:________

Name:_______________________________________________________

Age: _________

How long have you been fishing? _________

What was the main fishing gear that you used in the beginning of your career? _________________________________________________________

Which fishing gear do you use today? ____________________________

How would you describe the current conditions of fish stocks in Arraial do Cabo regarding abundance? ( ) unaltered ( ) declined ____% ( ) augmented____

Do you know of any species that were once important in commercial fishing, but today is overexploited in the region? ( ) Yes ( ) No

If Yes, which species? _____________________________________________

What are the main causes of overexploitation of these species’ stocks?___________________________________________________________

Do you know of any species that were formerly discarded or only used as bait that today are fishing targets? ( ) Yes ( ) No

If Yes, which species? ____________________________________________

How much time did you spend fishing at the beginning of your career?

( ) Days ____ ( ) Hours_____

How much time do you spend fishing today? ( ) Days ______ ( ) Hours_____
