# Supplementary material for: Telling the same story: Fishers and landing data reveal changes in fisheries on the Southeastern Brazilian Coast
Source: PLoS One. 2021 Jun 1;16(6):e0252391. doi: 10.1371/journal.pone.0252391 (PMC8168859; doi:10.1371/journal.pone.0252391)
Supplement: Attachment — Submitted filename: PONE-D-20-30952_reviewer-reviewFINAL.pdf [file pone.0252391.s002.pdf]

PLOS ONE

Fishing down the food web inferred from local ecological knowledge and landing data in the Southeastern Brazilian coast

--Manuscript Draft--

**Manuscript Number:**	PONE-D-20-30952
**Article Type:**	Research Article
**Full Title:**	Fishing down the food web inferred from local ecological knowledge and landing data in the Southeastern Brazilian coast
**Short Title:**	Fishers and landing data show fishing down the food web on the Southeastern Brazilian coast
**Corresponding Author:**	Carine de Oliveira, M.D<br>Universidade Federal de Santa Maria<br>Santa Maria, Rio Grande do Sul BRAZIL
**Keywords:**	Shifting baseline; marine resources; overfishing; local ecological knowledge; landing data; fisheries management
**Abstract:**	A complete understanding of the effects of fishing on marine ecosystems relies on information about the most conserved state of these environments. Non-conventional approaches such as the use of historical data and local ecological knowledge can provide information and help adjust our environmental references. Also, the combination of different types of data can indicate patterns that would be undetectable when evaluated separately. Here we investigated changes in fisher's perceptions regarding overexploited and new target species in artisanal fisheries in a secular fishing village of subtropical Brazil. Using landing data, we identified temporal changes in landing catches and in the mean trophic level (MTL) of high trophic level species (≥ 3.5 and >4) over 16 years. Fishers' knowledge revealed shifts in perception associated with years of fishing practice. More experienced fishers recognized a greater number of overexploited and new target species than fishers in the beginning of their careers. Landing data has revealed declining catch trends of 72% for five large-bodied mesopredator species. Due to the overfishing of mesopredators, there was a shift in target species, towards lower trophic level fish that were previously discarded. Temporal changes in MTL corroborated the occurrence of fishing down the web, identified through the replacement of target species by local fishers. Our work reveals that multiple sources of information can be combined to establish historical baselines and improve the detection of change in marine ecosystems.
**Order of Authors:**	Carine de Oliveira, M.D<br>Carlos E.L. Ferreira<br>Jéssica Bornholdt<br>Moysés. C. Barbosa<br>Vinicius J. Giglio<br>Mariana G. Bender
**Additional Information:**	
**Question**	**Response**
**Financial Disclosure**<br><br>Enter a financial disclosure statement that describes the sources of funding for the work included in this submission. Review the submission guidelines for detailed requirements. View published research articles from *PLOS ONE* for specific	C.O.F. acknowledges Coordenação de Aperfeiçoamento de Pessoal de Nível Superior (CAPES) for financial support. V.J.G. received a post-doctoral grant #2017/22273-0, São Paulo Research Foundation (FAPESP). CELF is supported by grants from FAPERJ and CNPq. Costão Rochoso Project (FUNBIO Grant Pesquisa Marinha 020/2017) has provided financial, and logistic support during field sampling in Arraial do Cabo. This work is part of a research program supported by Serrapilheira Institute (grant number Serra-1708-15364) awarded to Guilherme Ortigara Longo (PI).

Powered by Editorial Manager® and ProduXion Manager® from Aries Systems Corporation

examples.<br><br>This statement is required for submission and **will appear in the published article** if the submission is accepted. Please make sure it is accurate.<br><br>**Unfunded studies**<br>Enter: *The author(s) received no specific funding for this work.*<br><br>**Funded studies**<br>Enter a statement with the following details:<br>• Initials of the authors who received each award<br>• Grant numbers awarded to each author<br>• The full name of each funder<br>• URL of each funder website<br>• Did the sponsors or funders play any role in the study design, data collection and analysis, decision to publish, or preparation of the manuscript?<br>• **NO** - Include this sentence at the end of your statement: *The funders had no role in study design, data collection and analysis, decision to publish, or preparation of the manuscript.*<br>• **YES** - Specify the role(s) played.<br><br>* typeset	
**Competing Interests**<br><br>Use the instructions below to enter a competing interest statement for this submission. On behalf of all authors, disclose any competing interests that could be perceived to bias this work—acknowledging all financial support and any other relevant financial or non-financial competing interests.<br><br>This statement **will appear in the published article** if the submission is accepted. Please make sure it is accurate. View published research articles from *PLOS ONE* for specific examples.	NO authors have competing interests

Powered by Editorial Manager® and ProduXion Manager® from Aries Systems Corporation

**NO authors have competing interests**<br><br>Enter: *The authors have declared that no competing interests exist.*<br><br>**Authors with competing interests**<br><br>Enter competing interest details beginning with this statement:<br><br>*I have read the journal's policy and the authors of this manuscript have the following competing interests: [insert competing interests here]*<br><br>* typeset	
**Ethics Statement**<br><br>Enter an ethics statement for this submission. This statement is required if the study involved:<br><br>• Human participants<br>• Human specimens or tissue<br>• Vertebrate animals or cephalopods<br>• Vertebrate embryos or tissues<br>• Field research<br><br>Write "N/A" if the submission does not require an ethics statement.<br><br>General guidance is provided below. Consult the submission guidelines for detailed instructions. **Make sure that all information entered here is included in the Methods section of the manuscript.**	The interviews were approved by the Ethics Committee of Universidade Federal de Santa Maria, Brazil (CAAAE 29157919.6.0000.5346). The form of consent obtained was written.

**Format for specific study types**<br><br>**Human Subject Research (involving human participants and/or tissue)**<br>• Give the name of the institutional review board or ethics committee that approved the study<br>• Include the approval number and/or a statement indicating approval of this research<br>• Indicate the form of consent obtained (written/oral) or the reason that consent was not obtained (e.g. the data were analyzed anonymously)<br><br>**Animal Research (involving vertebrate animals, embryos or tissues)**<br>• Provide the name of the Institutional Animal Care and Use Committee (IACUC) or other relevant ethics board that reviewed the study protocol, and indicate whether they approved this research or granted a formal waiver of ethical approval<br>• Include an approval number if one was obtained<br>• If the study involved *non-human primates*, add *additional details* about animal welfare and steps taken to ameliorate suffering<br>• If anesthesia, euthanasia, or any kind of animal sacrifice is part of the study, include briefly which substances and/or methods were applied<br><br>**Field Research**<br><br>Include the following details if this study involves the collection of plant, animal, or other materials from a natural setting:<br>• Field permit number<br>• Name of the institution or relevant body that granted permission	
**Data Availability**<br><br>Authors are required to make all data underlying the findings described fully available, without restriction, and from the time of publication. PLOS allows rare exceptions to address legal and ethical concerns. See the PLOS Data Policy and FAQ for detailed information.	No - some restrictions will apply

A Data Availability Statement describing where the data can be found is required at submission. Your answers to this question constitute the Data Availability Statement and **will be published in the article**, if accepted.<br><br>**Important:** Stating 'data available on request from the author' is not sufficient. If your data are only available upon request, select 'No' for the first question and explain your exceptional situation in the text box.<br><br>Do the authors confirm that all data underlying the findings described in their manuscript are fully available without restriction?	
**Describe where the data may be found in full sentences. If you are copying our sample text, replace any instances of XXX with the appropriate details.**<br><br>• If the data are **held or will be held in a public repository**, include URLs, accession numbers or DOIs. If this information will only be available after acceptance, indicate this by ticking the box below. For example: *All XXX files are available from the XXX database (accession number(s) XXX, XXX.).*<br>• If the data are all contained **within the manuscript and/or Supporting Information files**, enter the following: *All relevant data are within the manuscript and its Supporting Information files.*<br>• If neither of these applies but you are able to provide **details of access elsewhere**, with or without limitations, please do so. For example:<br><br>*Data cannot be shared publicly because of [XXX]. Data are available from the XXX Institutional Data Access / Ethics Committee (contact via XXX) for researchers who meet the criteria for access to confidential data.*<br><br>*The data underlying the results presented in the study are available from (include the name of the third party*	Data cannot be shared publicly because of the questionnaires applied to fishers and it is not possible to disclose the names of the participants. Data are available from the Ethics Committee of Universidade Federal de Santa Maria, Brazil (contact via cep.ufsm@gmail.com) for researchers who meet the criteria for access to confidential data.

and contact information or URL).<br>• This text is appropriate if the data are owned by a third party and authors do not have permission to share the data.<br><br>* typeset	
Additional data availability information:	Tick here if your circumstances are not covered by the questions above and you need the journal’s help to make your data available.; Tick here if the URLs/accession numbers/DOIs will be available only after acceptance of the manuscript for publication so that we can ensure their inclusion before publication.

Powered by Editorial Manager® and ProduXion Manager® from Aries Systems Corporation

Manuscript
Click here to access/download;Manuscript;Manuscript_Fogliarini et al_Plos

Fishers and landing data show fishing down the food web on the Southeastern Brazilian coast

Carine O. Fogliarini[a*], Carlos E.L. Ferreira[b], Jéssica Bornholdt[a], Moysés. C. Barbosa[b], Vinicius J. Giglio[c], Mariana G. Bender[a]

[a]Marine Macroecology and Conservation Lab, Departamento de Ecologia e Evolução, Universidade Federal de Santa Maria. Avenida Roraima, 1000, prédio 17 sala 1140, Bairro Camobi, Santa Maria, Rio Grande do Sul, Brazil.

[b]Reef Systems Ecology and Conservation Lab, Departamento de Biologia Marinha, Universidade Federal Fluminense, Niterói, RJ, Brazil.

[c]Marine Conservation and Ecology Lab, Instituto do Mar, Universidade Federal de São Paulo, Santos, São Paulo, Brazil.

* Corresponding author
E-mail: carine_fogliarini@hotmail.com

Abstract

A complete understanding of the effects of fishing on marine ecosystems relies on information about the most conserved state of these environments. Non-conventional approaches such as the use of historical data and local ecological knowledge can provide information and help adjust our environmental references. Also, the combination of different types of data can indicate patterns that would be undetectable when evaluated separately. Here we investigated changes in fisher's perceptions regarding overexploited and new target species in artisanal fisheries in a secular fishing village of subtropical Brazil. Using landing data, we identified temporal changes in landing catches and in the mean trophic level (MTL) of high trophic level species ($\geq$ 3.5 and >4) over 16 years. Fishers' knowledge revealed shifts in perception associated with years of fishing practice. More experienced fishers recognized a greater number of overexploited and new target species than fishers in the beginning of their careers. Landing data has revealed declining catch trends of 72% for five large-bodied mesopredator species. Due to the overfishing of mesopredators, there was a shift in target species, towards lower trophic level fish that were previously discarded. Temporal changes in MTL corroborated the occurrence of fishing down the web, identified through the replacement of target species by local fishers. Our work reveals that multiple sources of information can be combined to establish historical baselines and improve the detection of change in marine ecosystems.

Keywords: Shifting baseline, marine resources, overfishing, local ecological knowledge, landing data, fisheries management

Introduction

Marine and coastal ecosystems are globally threatened due to human impacts such as overfishing, pollution, habitat destruction and climate change [1, 2]. Indeed, anthropogenic activities have been changing the oceans for millennia, with fishing being the most ancient impact to marine ecosystems [3]. ~~For a long period of time~~ fishing has been unsustainable [4], surpassing the recovery limits of several fish stocks across the globe [5]. Recent studies argue that fishing stocks are gradually recovering in different parts of the world [6], mainly in regions where there are effective fisheries management [7]. Although these recent findings show positive global scenarios [8] overfishing is responsible for substantially modifying the abundance and biomass patterns of species and can decrease individuals' body size of a population [9, 10, 11, 12]. Fisheries are directed towards top predators and select individuals with larger body sizes [13], a common pattern with known consequences for marine ecosystem functioning [14].

Fishing effort concentrated on large-bodied fish can cause the decline of top predators stocks such as groupers, sharks and tunas [15, 16, 17, 18, 19], and even drive species to local extinction [3, 20]. These large-bodied species tend to occupy higher levels in the food chain and play a key role in maintaining the trophic structure and functioning of communities [21, 22]. Thus, the removal of top predators can trigger cascading effects on lower levels of the food web [23, 22, 24]. Declining trends of higher trophic level stocks lead to a systematic replacement by lower trophic level and smaller-bodied target species, a process identified as "fishing down the food web" [25, 26, 27]. This process has been measured through a decline in fisheries' mean trophic level [28, 29, 30, 31]. In an attempt to understand how fishing impacts marine trophic webs, studies highlight that there are other ways in which fishing may be affecting the ecosystem. In "fishing through the food web" process a decline in MTL is caused due to the sequential addition of low trophic level rather than to a decline of high trophic level catches [23]. There is also "fishing up the food

web’, which corresponds to a shift from low to high trophic level species over time, consequently increasing the MTL of catches [32, 33]. Thus, multiple hypotheses can explain how fishing affects marine food webs [34].

The assessment of temporal changes in MTL and replacement of target species requires long-term fisheries monitoring. However, most low and lower-middle income countries lack fisheries monitoring programs [35]. In Brazil, such programs are rare and fragmented, and since 2008, there is no governmental fisheries monitoring program established at the national level. In addition, lack of financial resources, a large (~8000 km) and heterogeneous coast, a multigear fishing scenario, differential efforts and target species, pose a challenge to the implementation of fisheries management strategies in Brazil [36, 37]. To circumvent the difficulties caused by the lack of fishing data, non-conventional approaches can be useful to estimate the impacts of fishing over time [38, 39, 40]. For instance, local ecological knowledge (LEK) may provide a relevant source of historical information, in which marine resource users (e.g. fishers) experienced different scenarios in the past [41, 39, 42].

A robust assessment of the magnitude and effect of anthropogenic impacts (e.g. fishing) on ecosystems requires knowledge of the unexplored state of these environments [43]. However, throughout human generations, information on composition, size and abundance of species caught on fisheries may be lost, compromising the environmental perception adopted by human populations [44]. Such cross-generational change in baselines has implications to environmental issues, as it results in increased tolerance for progressive environmental degradation because the younger generations consider a more degraded environment as the norm [45, 46]. Pauly et al. [45] described this phenomenon as the “shifting baseline syndrome”, a psychological tendency to assume that "natural" conditions of an ecosystem are those first observed by users, researchers and/or contemporary

managers. When data on the conservation status of the environment is scarce, historical data from alternative sources (e.g. works of art, reports of naturalists, logbooks, photographs, grey literature, and anecdotal information) have been used to adjust references [47].

Fisher's LEK has been widely used to investigate the past of fisheries, mainly in countries that lack long-term monitoring. The empirical knowledge of natural resource users is a cost-effective alternative approach that offers relevant data to inform management. For instance, fishers LEK have provided information on temporal and spatial patterns of catches [48, 49, 50], behavior of targeted species [51, 52, 53, 54], changes in abundance and body size of targeted species [55, 18], and conflicts with other resource users and management interventions [56, 57]. In certain cases, LEK has been also associated with other types of data, such as landing data and underwater visual census, to reveal temporal trends in fish populations [49, 58, 59, 60, 61]. These combined approaches offer an opportunity to understand the effect of small-scale fisheries in data poor scenarios – when temporal and systematically collected data are missing. Combining anecdotal evidence from fisher's LEK and fisheries landing data, our study aims to identify temporal changes in (i) small-scale fisher's perception regarding overexploited and new target fish species; (ii) catches of overexploited and new target fish species. Specifically, using the MTL approach, we evaluated changes in local trophic structure caused by fishing. We also discuss the main causes of fish overexploitation and management strategies in a subtropical fisheries hotspot.

Materials and methods

Study site

The study was conducted in Arraial do Cabo, southeastern Brazil (Fig. 1). Arraial do Cabo is an important and ancient Brazilian fishing area, where a variety of artisanal fishing gear is employed such as hook and line, gillnet, spearfishing and beach seine. In Arraial do Cabo, artisanal fisheries occur with small boats such as canoes, which make trips near the shore, or using hook and line or small nets like cast nets in the rocky shore or on the beach [62]. The beach seine is a secular practice that was central to the establishment of the local partially protected marine protected area (MPA) – the Arraial do Cabo Marine Extractive Reserve. In this MPA, only local fishers are allowed to fish, with 1600 families relying on fishing activities as the main source of income [62]. Arraial do Cabo holds a diverse marine community, gathering tropical and warm temperate components, which is favored by the coastal morphology and local upwelling events [63, 64].

Fig 1. Map of Arraial do Cabo region (state of Rio de Janeiro, Brazil), showing interview sites.

Data collection

Fisher's local ecological knowledge

Between July and August 2018 and 2019, we interviewed 155 artisanal fishers on the fishing communities of Figueira, Monte Alto, Praia Grande, Praia dos Anjos, Prainha, and Pontal (Fig. 1). Interviewed fishers correspond to 10.3% of local artisanal fishers (n = 1500). Interviews were conducted following previous consent and individually, so that there was no influence from others on fishers' answers. Semi-structured questionnaires included questions on (i) which fish species were previously discarded but are now fishing targets; (ii) which species are overexploited in the region; and (iii) what are the main causes of species' overexploitation. The fishers answered spontaneously which species they

consider overexploited and new targets of fisheries. When completing the questionnaire (S1 File), fishers were asked to indicate the next interviewee through snowball sampling [65].

We also asked fishers their age and years of fishing experience. For data analysis, we chose to use the fisher's experience (time period) rather than age, as these two variables are correlated ($P < 0.001$, $r^2 = 0.53$, cor = 0.74, Pearson's correlation test). Fishers were categorized according to their fishing experience into four groups: beginner (<20 years, n = 24), intermediate (21-35 years, n = 55), experienced (36-40 years, n = 44) and very experienced (>40 years, n = 32). Under this classification, we consider only their time of experience (years of practice), with no qualitative judgment of the real experience of each fisher [66]. We assume that within a given category of experience, all fishers spend the same time fishing, and all gain experience at the same rate [66]. Still, this set of different categories can help estimate changes in fish composition as well as differences in perceptions of trends across generations of fishers. In addition, we chose these experience categories so that they would accommodate similar sample sizes. The small sample size of the beginners category compared to the very experienced is related to the lack of engagement with fishing by the younger generations. Younger fishers are switching to alternative sources of income such as tourism, often encouraged by their families to leave the fishing. The interviews were approved by the Ethics Committee of Universidade Federal de Santa Maria, Brazil (CAAAE 29157919.6.0000.5346) and by Sistema de Autorização e Informação em Biodiversidade (Sisbio-ICMBio/IBAMA/Brazil nr. 55911-6).

Fisheries landing data

We searched for landing data on the main species reported by the interviewees. Landing data (e.g. catch in kg) has been incorporated into the study to complement the trends identified by local fishers. Such data corresponds to local annual catches (kg) of

artisanal fisheries registered between 199[illegible]nd 2008 in the port of Arraial do Cabo (Fisheries landing monitoring report[illegible]PAC).

Mean trophic level

We compiled the trophic level (TL) of 81 species landed in Arraial do Cabo between 1992 [illegible] 2008 (S1 Table). The trophic levels were obtained from FishBase, which provides TL estimates from food items for many fish species [67]. For the TL of invertebrates, we used the equivalent database, SeaLifeBase [68].

To assess MTL trends in catches over time, we compared the MTL of species in four groups: all landed species, species with TL > 4, TL ≥ 3.5, and TL < 3.5. These categories allow us to assess changes in MTL taking into account the temporal dynamics of higher and lower trophic level species. Top predators are more sensitive to overfishing, thus the dynamic of species stocks with a threshold value of TL > 4 was examined in previous studies [23]. Species with TL > 3.5 (e.g. tunas, cod and groupers) feed on a mixture of low and high-TL organisms [69]. Species with TL < 3.5 were assessed in order to observe changes in the contribution of low trophic level species [69]. We used these threshold values to eliminate the masking effect and to emphasize changes in the relative abundance of fish from certain trophic groups. The MTL was estimated as follows [25]:

$$MTL = \frac{\sum TL_{ij} Y_{ij}}{\sum Y_{ij}}$$

where MTL is the mean trophic level of landing in year j, Yij the landing of species i in year j and TLi is the trophic level of species i.

We used a null model approach to test whether citations of overexploited and new target species (by fishers), within a given trophic level, are more frequent than expected at random. We generated 1000 random samples for the number of citations of each species

within the overexploited and new target species groups. For each random sample, we calculated the MTL weighted by the citation number, using the following equation:

$$MTLc = \frac{\sum TLi\ Ci}{\sum Ci}$$

where MTL_c is the mean trophic level of overexploited and new target species and Ci is the total number of citations of species i.

We then plotted the confidence intervals (C.I. 95%) of the null MTL obtained for overexploited and new target species and the observed MTL_c, to verify if the citation frequency differs than expected at random.

Data analysis

To estimate changes in fishers' perception of target species, we applied a Generalized Linear Model (GLM) between the (i) number of overexploited species and (ii) number of new target species vs. experience of fishers (years of practice). The number of cited species was set as the response variable. Because numbers of species are countable data, we assumed a Poisson distribution. The experience represents the explanatory variable. Under these specifications, the equation of model was:

$$log(\mu i) = \beta 0 + \beta 1\ xi$$

Where μi is the conditional expectation of yi, E(y|x), $\beta 0$ is the coefficient marked (Intercept) and $\beta 1$ the coefficient marked x. We assumed the model of the mean number of species μi using a log link.

We measured the mean percent of increase in citations (P) of overexploited fish species and new target species according to fishers' experience (F) using the GLM model coefficients (*coef1* and *coef2*):

$$P = \left(\frac{e^{(coef1+coef2*F)}}{e^{(coef1+coef2*0)}} - 1 \right) * 100$$

To test for differences between the proportion of overexploited and new target species (previously discarded) mentioned by fishers in different experience categories, we applied a Z-test. We then verified differences between groups through a pairwise comparison of proportions. Regression analysis were used to measure temporal catch trends of fish species by plotting the landed weight (in tons) over the years. All analyzes were performed on R software v. 3.6.1 [70].

Results

Fishers with more years of practice recognized a significantly greater number of overexploited fish species than those with fewer years of practice (β = 0.009, df = 153, P < 0.001) (Fig. 2a). This relationship can also be observed by an increase in the mean percent in the citation, which relates with years of practice (Fig. 2b). We observed the same pattern for the number of species recognized as new target species. As time of practice increases, fishers mentioned a significantly greater number of species as new targets of local fisheries (β = 0.010, df = 153, P = 0.01) (Fig. 2c). In addition, there is an increase in the percent of citations of new target species by fishers' with more years of practice (Fig. 2d).

Fig 2. (a) Number of species mentioned as overexploited according to fisher's experience (years of practice); (b) Probability of citations of overexploited species according to fisher's experience; (c) Number of species mentioned as new target species according to fisher's

experience; and (d) Probability of citations of new target species according to fisher's experience.

In total, 37 species were identified as overexploited by local fishers (Table 1). The bluefish (*Pomatomus saltatrix*) was the most frequently cited species, across all experience categories. The proportion of citations of *P. saltatrix* were similar between categories, with at least 45% of fishers in different experience categories recognizing bluefish as overexploited: 56% of very experienced (n = 18), 55% of experienced (n = 21), 47% of intermediate (n = 18), and 45% of beginner fishers (n = 21) (Fig. 3a). The second most cited species was the largehead hairtail (*Trichiurus lepturus*), although there are no significant differences between the experience categories, the species was more mentioned by intermediates (26%, n = 10) and beginners (30%, n = 14) fishers. The dusky grouper (*Epinephelus marginatus*) and the crevalle jack (*Caranx hippos*) were most frequently cited by very experienced fishers (> 40 years of practice) and had a significant difference in the proportion of citations between very experienced and beginners (P = 0.03 and P = 0.001, respectively). In addition to *C. hippos,* the lesser amberjack (*Seriola fasciata*) was another carangid referred to as overexploited (Fig. 3a).

Table 1. Species reported as overexploited by fishers, in descending order of number of citations (N). Percentage of citation (%) of species by fishers (n = 155). Trophic levels were obtained from FishBase where a higher value means higher trophic levels (Froese and Pauly, 2019). Trophic categories were obtained from the Southwestern Atlantic reef fish database (Pinheiro et al., 2018). The conservation status at global level derives from the IUCN Red List of Threatened Species (IUCN, 2019) and at national level from the Brazilian Red List of Threatened Species (MMA, 2018).

OVEREXPLOITED SPECIES

Order	Species	N	%	Trophic level	Trophic category	Global status	Regional status
1	*Pomatomus saltratix*	78	50.3	4.5	MCAR	VU	NT
2	*Trichiurus lepturus*	37	23.8	4.4	MCAR	LC	LC
3	*Epinephelus marginatus*	23	14.8	4.4	MCAR	VU	VU
4	*Seriola fasciata*	22	14.1	4.5	MCAR	LC	DD
5	*Caranx hippos*	20	12.9	3.6	MCAR	LC	LC
6	*Pseudopercis numida*	20	12.9	3.9	MCAR	LC	NT
7	*Hyporthodus niveatus*	16	10.3	4	MCAR	VU	VU
8	*Seriola lalandi*	14	9	4.2	MCAR	LC	LC
9	*Mugil liza*	11	7	2	HERB	DD	NT
10	*Scomberomorus cavalla*	11	7	4.4	MCAR	LC	LC
11	*Sardinella brasiliensis*	10	6.4	3.1	PLANK	DD	DD
12	*Conger orbignyanus*	10	6.4	3.7	MCAR	LC	DD
13	*Sarda sarda*	8	5	4.5	MCAR	LC	LC
14	*Cynoscion striatus*	7	5	3.9	MCAR	NE	NE
15	*Mycteroperca microlepis*	6	3.8	3.7	MCAR	VU	DD
16	*Coryphaena hippurus*	6	3.8	4.4	MCAR	LC	LC
17	*Seriola dumerili*	6	3.8	4.5	MCAR	LC	LC
18	*Priacanthus arenatus*	5	3.2	4	MINV	LC	LC

19	*Katsuwonus pelamis*	5	3.2	4.4	MCAR	LC	LC
20	*Caranx crysos*	4	2.5	4.1	MCAR	LC	LC
21	*Caranx latus*	4	2.5	4.2	MCAR	LC	LC
22	*Pagrus pagrus*	3	1.9	3.9	MINV	LC	DD
23	*Trachinotus carolinus*	3	1.9	3.5	MINV	LC	LC
24	*Micropongonias furnieri*	3	1.9	3.1	MINV	LC	LC
25	*Cynoscion acoupa*	3	1.9	4.1	MCAR	LC	NT
26	*Lopholatilus villarii*	2	1.2	3.8	MCAR	NE	VU
27	*Balistes vetula*	2	1.2	3.8	MINV	NT	NT
28	*Diplodus argenteus*	2	1.2	3.1	OMNI	LC	LC
29	*Peprilus paru*	2	1.2	4.5	MCAR	LC	LC
30	*Centropomus undecimalis*	2	1.2	4.2	MCAR	LC	LC
31	*Scomber colias*	1	0.6	3.4	MCAR	LC	LC
32	*Scomberomorus brasiliensis*	1	0.6	3.3	MCAR	LC	LC
33	*Aluterus monoceros*	1	0.6	3.8	OMNI	LC	NT
34	*Lagocephalus laevigatus*	1	0.6	4	MCAR	LC	LC
35	*Carcharhinus leucas*	1	0.6	4.3	MCAR	NT	NT
36	*Isurus oxyrinchus*	1	0.6	4.5	MCAR	NT	NT
37	*Pseudobatos horkelii*	1	0.6	3.8	MINV	CR	CR

Trophic categories: Macrocarnivore (MCAR); Mobile Invertivore (MINV); Omnivore (OMNI); Planktivore (PLANK); Herbivore (HERB). IUCN categories: Critically endangered (CR); Vulnerable (VU); Near threatened (NT); Least Concern (LC); Data deficient (DD); Not evaluated (NE).

Fig 3. Fish species most frequently cited as overexploited (a) and new target species (b) according to fisher's experience (years of practice). Letters indicate significance ($P < 0.05$) between categories of experience of each species.

Fishers identified 36 new target species that were previously discarded by local fisheries (Table 2). *T. lepturus* was cited by 63% of very experienced fishers, 76% of experienced, 71% of intermediates, and 66% of beginners (Fig. 3b). The second most cited species was the grey triggerfish (*Balistes capriscus*), followed by the Argentine conger (*Conger orbignyanus*), the unicorn leatherjacket filefish (*Aluterus monoceros*) and the Atlantic bigeye (*Priacanthus arenatus*) (Fig. 3b). For *T. lepturus* and *C. orbignyanus* the proportion of citations was similar across experience categories (Fig. 3b). The citations for *P. arenatus* decreased with fisher's experience, therefore being greater for beginner fishers. Only the citations of *B. capriscus* had significant differences between experience categories, being proportionally higher for experienced compared to intermediate fishers ($P = 0.01$) (Fig. 3b).

Table 2. Species cited as new targets by fishers, in descending order of reports (N). Percentage of citation (%) of species by fishers (n = 155). Trophic levels were obtained from FishBase where a higher value means higher trophic levels (Froese and Pauly, 2019). Trophic categories were obtained from the Southwestern Atlantic reef fish database (Pinheiro et al., 2018). The conservation status at global level derives from the IUCN Red List of Threatened Species (IUCN, 2019) and at national status from the Brazilian Red List of Threatened Species (MMA, 2018).

**NEW TARGET SPECIES**

Order	Species	N	%	Trophic level	Trophic category	Global status	Regional status
1	*Trichiurus lepturus*	107	69	4.4	MCAR	LC	LC
2	*Balistes capriscus*	27	17.4	4.1	MINV	VU	NT
3	*Conger orbignyanus*	26	16.7	3.7	MCAR	LC	DD
4	*Aluterus monoceros*	24	15.4	3.8	OMNI	LC	NT
5	*Priacanthus arenatus*	23	14.8	4	MINV	LC	LC
6	*Scomber colias*	13	8.3	3.4	MCAR	LC	LC
7	*Sardinella brasiliensis*	12	7.7	3.1	PLANK	DD	DD
8	*Katsuwonus pelamis*	11	7	4.4	MCAR	LC	LC
9	*Lagocephalus laevigatus*	9	5.8	4	MCAR	LC	LC
10	*Diplodus argenteus*	8	5.1	3.1	OMNI	LC	LC
11	*Porichthys porosissimus*	5	3.2	3.7	MINV	NE	LC
12	*Mugil liza*	4	2.5	2	HERB	DD	NT
13	*Pomatomus saltratix*	4	2.5	4.5	MCAR	VU	NT
14	*Micropogonias furnieri*	4	2.5	3.1	MINV	LC	LC
15	*Cynoscion striatus*	3	1.9	3.9	MCAR	NE	NE
16	*Umbrina canosai*	3	1.9	3.9	MINV	NE	LC
17	*Selene setapinnis*	3	1.9	3.7	MCAR	LC	LC
18	*Dactylopterus volitans*	3	1.9	3.7	MINV	LC	LC
19	*Trachinotus carolinus*	2	1.2	3.5	MINV	LC	LC
20	*Caranx latus*	2	1.2	4.2	MCAR	LC	LC
21	*Cynoscion acoupa*	2	1.2	4.1	MCAR	LC	NT
22	*Chloroscombrus chrysurus*	2	1.2	3.5	PLANK	LC	LC
23	*Seriola fasciata*	2	1.2	4.5	MCAR	LC	DD
24	*Pseudopercis numida*	2	1.2	3.9	MCAR	LC	NT
25	*Carcharhinus plumbeus*	1	0.6	4.5	MCAR	VU	CR
26	*Isurus oxyrinchus*	1	0.6	4.5	MCAR	NT	NT
27	*Squatina argentina*	1	0.6	4.1	MCAR	EN	CR
28	*Sparisoma axillare*	1	0.6	2	HERB	DD	VU
29	*Epinephelus marginatus*	1	0.6	4.4	MCAR	VU	VU
30	*Carcharhinus brevipinna*	1	0.6	4.2	MCAR	VU	DD

31	*Cynoscion jamaicensis*	1	0.6	3.8	MCAR	LC	LC
32	*Pseudobatos horkelii*	1	0.6	3.8	MINV	CR	CR
33	*Lopholatilus villarii*	1	0.6	3.8	MCAR	NE	VU
34	*Cynoscion guatucupa*	1	0.6	3.7	MCAR	NE	LC
35	*Pagrus pagrus*	1	0.6	3.9	MINV	LC	DD
36	*Sarda sarda*	1	0.6	4.5	MCAR	LC	LC

Trophic categories: Macrocarnivore (MCAR); Mobile Invertivore (MINV); Omnivore (OMNI); Planktivore (PLANK); Herbivore (HERB). IUCN categories: Critically endangered (CR); Vulnerable (VU); Near threatened (NT); Least concern (LC); Data deficient (DD); Not evaluated (NE).

The same declining trend reported by fishers for *P. saltatrix*, *T. lepturus, E. marginatus, C. hippos,* and *S. fasciata* (Fig. 3a) were revealed by fisheries landing data (Fig. 4). Since the 1990s, *P. saltatrix* stocks declined significantly (i.e. approximately 47%) ($P < 0.001$) (Fig. 4a), this status being perceived by fishers across all experience categories. For *T. lepturus*, the catches declined significantly after the 2000s ($P < 0.001$) (Fig. 4b), which has been identified by the majority of fishers in less experienced categories. This species has been considered overexploited and also identified as a new target species in the region. Landing data show that since the 1990s, catches of *E. marginatus* declined 61%, *C. hippos* catches diminished by 88%, and *S. fasciata* by 89%, the latter showed significant declining trends ($P < 0.001$) (Fig. 4c, e, g). This overexploitation scenario was observed by very experienced fishers, which cited the first two species as overexploited in a higher proportion (Fig. 3a).

Fig 4. Artisanal fisheries (tons) landings in Arraial do Cabo from 1992 to 2008. Species on the left (a, c, e, g) correspond to those most frequently cited as overexploited in interviews and on the right (d, f, h), new target species reported by local fishers. (b) *Trichiurus lepturus* corresponds to both an

overexploited and a new target species. a, b, d, f, g and h, are second-order polynomial regressions; c and e, an exponential regression.

The catches of the new target species exhibited an increase from the 2000s onwards, followed by a reduction in the amount landed catch over the years (Fig. 4b, d, f, h). From the 1990s to 2000s, new target species as *T. lepturus*, *C. orbignyanus*, and *A. monoceros* had an increase in landed catches (Fig. 4b, d, h). By the mid-2000s, catches of these species declined 69%, 55% and 85%, respectively (Fig. 4b, d, h). Fishers also reported this trend, when respondents mentioned these species as overexploited (Fig. 3b; Table 1). Landings of *P. arenatus* had a significant increase from 2000 onwards ($P < 0.001$) (Fig. 4f), mainly reported by beginner fishers (Fig. 3b).

Catch MTL of TL ≥ 3.5 and TL > 4 species declined significantly from 1992 to 2008 ($r^2 = 0.26$; $P = 0.03$ and $r^2 = 0.43$; $P < 0.001$, respectively) (Figure 5a, b). When MTL of all landed species are represented, there was a non-significant increase in MTL over time ($r^2 = 0.06$; $P = 0.32$). The same increasing trend was observed with species with TL < 3.5 ($r^2 = 0.07$; $P = 0.14$) (Fig. 5a, b). In the years of 1995 and 1999, there were peaks in landings of all species in Arraial do Cabo (Fig. 5c). However, between 2000 and 2004, there were sharp declines in landings (TL > 4, TL ≥ 3.5, all species, and TL < 3.5) (Fig. 5c).

Fig 5. Trends in MTL of species landed in Arraial do Cabo from 1992 to 2018. (a) Linear regression of catch MTL; (b) moving average of MTL catch; and (c) Landing catch in tons. The colors represent MTL calculated after excluding all species below trophic level 4 (blue), calculated after excluding all species below trophic level 3.5 (yellow), calculated with all species landed (red), and only species with trophic level below 3.5 (green).

When contrasting the MTL of species mentioned by fishers as overexploited and as new target species, we observed that the citations of overexploited species are more frequently within higher TL species relative to new target ones (S1 Fig.). In addition, the MTL observed for overexploited species was higher than expected by chance (S1 Fig.).

Fishers mentioned 35 different possible causes of fisheries overexploitation in Arraial do Cabo (1.58 ± 1.08, mean ± standard deviation). The five most mentioned causes were the increased fishing effo[illegible]22%), which is recognized by fishers as an increase in the number of fishers and boats; the presence of industrial fishing vessels (21%), the presence of purse seine fisheries (19%), gillnet fishing (16%), and trawling (12%).

Discussion

Our results show that fisher's perception of overexploited and new target species is shaped by their fishing experience. As experience increases with years of fishing practice, fishers are able to recognize a greater number of overexploited and new target species. These findings indicate the occurrence of a shifted baseline among fishers' generations, which in this case, is the loss in fisher perception regarding changes in fisheries' composition and the decline of fish species throughout generations [45]. At the time young and less experienced fishers started fishing, several species were already overexploited, compromising their current perception of fish stocks. The small number of species reported by less experienced fishers compared with experienced ones may reflect the lack of familiarity and/or a reduced interaction of fishers with the natural environment [44]. Such disconnection with past conditions has effects on the willingness and motivation of new generations to accept management restrictions aimed to restore resources and even mislead recovery perceptions, the "placebo effect" [71]. In Arraial do Cabo, the overexploitation of fishing resources [49, 19] has made less experienced fishers look for alternative income sources. Several young

fishers have mentioned being involved in any type of tourism activities, using their boats for sightseeing, as a parallel activity to fishing. This reduces the engagement of young people with fishing and possibly impairs their perception of the status of fishing resources.

Our results revealed declining trends in catches of the macrocarnivores *P. saltatrix, E. marginatus, C. hippos,* and *S. fasciata* in Arraial do Cabo. A previous survey showed a decline of 80% in *P. saltatrix* catches over the past six decades, and reductions in the abundance and body size of *E. marginatus* for the same period [49]. These two species, one being pelagic and one reef-associated, are important resources in Arraial do Cabo, as well as along the south and southeastern Brazilian coast [72]. Our findings highlight that very experienced fishers have identified the overexploited status of *E. marginatus* and *C. hippos* populations in a greater proportion. Previous studies show that more experienced fishers have reported higher catches of groupers than less experienced ones [55, 15, 48, 73, 18, 66], this being a common scenario of overexploitation in small-scale fisheries. For a long time, groupers were abundant in Arraial do Cabo, and generations of more experienced fishers witnessed large catches of the species in the past. Today, older fishers perceive this decline, and catches of groupers have become rare events in the region. Due to life history traits such as high longevity, slow growth, late maturation, and low reproductive rates, groupers are highly sensitive to fishing effects [74, 75]. Such characteristics have contributed to the threatened status of many grouper species worldwide [14, 76]. The decline of other predator species as *C. hippos* and *S. fasciata* is concerning, since these species are important regional fishing resources [77], and as expected for large carnivores, depict a combination of biological traits that can enhance their extinction risk [78].

Large catches of *T. lepturus* in Arraial do Cabo occurred incidentally in the past, i.e. when fishers targeted other species with high commercial value such as *P. saltatrix* and *E. marginatus*. The largehead hairtail was identified both as overexploited and new target

species, mostly by younger fisher. Fishing of this species is more recent than of other fishes mentioned as overexploited. Thus, knowledge about the exploitation status of this species is more widespread among young generations. Due to its low commercial value and lack of consumer market, large quantities of *T. lepturus* were previously discarded. Later on, the decline of large predators in the region [49, 19] led fishers to replace their target species. This has made *T. lepturus* economically valuable and a target of local fisheries, which was also observed for *B. capriscus, C. orbignyanus* and *A. monoceros*. Fishing directed at other less valuable species has become an alternative to maintain local fishing activities. The collapse of higher trophic level species caused by overfishing led fishers to target less valuable and lower trophic level species, a case of fishing down marine food webs [25].

The fishing down the food web process predicts continuous declines in catches of high trophic level and increases in catches of lower trophic level species [25]. This ubiquitous phenomenon has been identified in several ecosystems, both at local and global scales [79, 28, 80, 67, 81]. Here, we demonstrate the occurrence of this phenomenon in Arraial do Cabo, combining fishers' LEK, landing data and the MTL index ($TL \geq 3.5$ and $TL > 4$) [25]. In particular, fishing is an ancient activity in the region, and marine resources have been exploited for a long time. Sharp declines in the catches of top predators, as previously reported by fishers, are one of the consequences of the overfishing scenario in this region. It is therefore essential to understand how fishing affects food webs for efficient fisheries management in Arraial do Cabo. In contrast, MTL of all landed species shows an increasing trend in MTL over time. This effect, known as false "fishing up", was identified for fisheries in the Mediterranean Sea and in some upwelling systems [82, 83]. Catch MTL assessments considering all landed species can induce a "masking effect" [84, 81]. Several factors are responsible for masking the prevalence of the fishing down process [85, 86], including taxonomic over-aggregation, the body size of species and geographical expansion

of fisheries [31]. In contrast, disregarding expansion leads to a misinterpretation of the increase in MTL, as reported in previous studies [87]. Ultimately, studies encourage the use of MTL as a complementary tool for the assessment of the effects of fishing on ecosystems [88].

Fishers LEK revealed that, in the past, large catches of tunas, sharks and groupers were common in Arraial do Cabo (Fig. 6). Shark species such as the spinner shark (*Carcharhinus brevipinna*) were caught in large quantities (Fig. 6b), as well as the and currently threatened species the Brazilian guitarfish (*Pseudobatos horkelii*) and the sandbar shark (*Carcharhinus plumbeus*) [49]. Despite never being a major fishing target, these shark species are still caught in the region, but in smaller quantities. The current fishing scenario revealed in the present study is quite different from that reported by the old fishers. Despite its low trophic level, the large body size of parrotfishes such as the endemic greenbeak parrotfish (*Scarus trispinosus*) has made this species a major target of artisanal fisheries in the Southwestern Atlantic [89, 49, 90]. In Arraial do Cabo, a past study identified reductions in the abundance and body size of *S. trispinosus* individuals [49]. In addition, spearfishers commonly catch other large parrotfishes as those of the *Sparisoma* genus, indicating shifts in the composition of target species [49].

Fig 6. Photographs showing past and present captures in Arraial do Cabo. (a) Catch of the *Epinephelus marginatus* in 1982; (b) *Carcharhinus brevipinna* caught in Praia Grande in 2005; (c) Catch of *Balistes capriscus* and (d) beach seine fishing at Praia Grande recorded in 2020.

The cause more mentioned by fishers as responsible for fisheries overexploitation was increase in the fishing effort. While local reports have revealed a declining trend in the number of boats (S2 Table, S2 Fig. a), fishing effort in the number of fishing days has

increased over time (S2 Table, S2 Fig. b). Due to the overexploited state of fish stocks, fishers have to spend more time fishing to obtain the same yields as in the past. Another cause of overexploitation reported by fishers was purse seine. This fishing gear has a high catch potential, and it is less selective when compared to other artisanal fishing gear (e.g. line and hook). For example, in 2019, purse seine catches represented 59% (231.16 kg) of the total landed catch in the region (S2 Fig.). In addition, purse seine effort in fishing days corresponded to only 6% of the total fishing days (S2 Fig.). Therefore, despite changes in fishing effort, the scenario of species overexploitation is worrisome and can be even greater than reported in our study.

Our results provide evidence that management initiatives have been ineffective to change the decline trends of fishing resources in Arraial do Cabo. Classical management strategies as fishing quotas with quantities determined after stock assessments should be implemented based on species' conservation status. Threatened species as *E. marginatus*, *Hyporthodus niveatus*, *Lopholatilus villarii,* and *Sparisoma axillare*, which were mentioned by fishers as overexploited and new target species (Table 1 and 2), should not be targeted until stocks are recovered. As an alternative to minimize illegal fishing off the season and the incidental catch of juveniles, resource users should be oriented to target healthy fish stocks or to search for alternative income sources such as community-based ecotourism initiatives. A previous study has compared the effects of partially protected areas vs. unprotected areas in the abundance and body size of reef fish, including a site in Arraial do Cabo [91]. Groupers had higher density and relative abundance in a partially protected area, indicating that even small no-take areas can function to recovery some fish populations [91]. The case of Arraial do Cabo is not different from many reef sites around the world, where the fishing effort has surpassed the recovery potential of natural resources [92]. The good news is that even at relatively small areas groupers can increase in density and size if

fishing ceases [91]. Additional ongoing tagging experiments in the region indicate relatively short home ranges for parrotfishes (authors, per. com.). Small MPAs appear as a critical tool to preserve local resources, but more importantly, to show local users their efficiency in rebuilding stocks. We also highlight the need for greater inspection by the authorities to prevent illegal fishing by industrial vessels such as trawl and gillnet fisheries that occur even within the MPA. Environmental education programs aimed at local fishers could minimize the lack of knowledge on the exploitation state of resources, and the influence of the shifting baseline syndrome among fishers.

Here we demonstrated that experienced fishers better recognize changes in fisheries and in marine ecosystems when compared to less experienced ~~ones.~~ In addition, we identified changes in composition and abundance of pelagic and demersal, reef-associated fish species caught in a ~~secular~~ fishing village using fishers' LEK and fisheries landing data. Despite the short time series, fishing monitoring data reveals changes in the MTL over time, indicating the occurrence of fishing down the food web. The combination of multiple sources of information can help prevent biases as underestimated assessments of the impact of fishing [93]. By incorporating past data, it is possible to identify changes in catches over time, as well as to adjust conservation goals and strategies for marine ecosystems.

Acknowledgments

C.O.F. acknowledges Coordenação de Aperfeiçoamento de Pessoal de Nível Superior (CAPES) for financial support. V.J.G. received a post-doctoral grant #2017/22273-0, São Paulo Research Foundation (FAPESP). CELF is supported by grants from FAPERJ and CNPq. Costão Rochoso Project (FUNBIO Grant Pesquisa Marinha 020/2017) has provided financial, and logistic support during field sampling in Arraial do Cabo. This work is part of a research program supported by Serrapilheira Institute (grant number Serra-1708-15364)

awarded to Guilherme Ortigara Longo (PI). In addition, we thank fishers for their collaboration and shared knowledge. ICMBio has ever been supportiveness for our projects.

References

1. Worm B, Barbier EB, Beaumont N, Duffy JE, Folke C, Halpern BS, et al. Impacts of biodiversity loss on ocean ecosystem services. Science. 2006; 314 (5800): 787-790.
2. Harnik PG, Lotze HK, Anderson SC, Finkel ZV, Finnegan S, Lindberg DR et al. Extinctions in ancient and modern seas. Trends Ecol Evol. 2012; 27 (11): 608-617.
3. Jackson JBC, Kirby MX, Berger WH, Bjorndal KA, Botsford LW, Bourque BJ, et al. Historical overfishing and the recent collapse of coastal ecosystems. Science. 2001; 293 (5530): 629-637.
4. Jennings S, Kaiser MJ. The effects of fishing on marine ecosystems. In Advances in Marine Biology. Academic Press. 1998; 34, pp. 201-352.
5. Pauly D, Zeller D. Catch reconstructions reveal that global marine fisheries catches are higher than reported and declining. Nat Commun. 2016; 7, 10244.
6. Knowlton N. Ocean Optimism: Moving Beyond the Obituaries in Marine Conservation. Annu Rev Mar Sci. 2020; 13.
7. Hilborn R, Amoroso RO, Anderson CM, Baum JK, Branch TA, Costello C, et al. Effective fisheries management instrumental in improving fish stock status. PNAS. 2020; 117 (4): 2218-2224.
8. Duarte CM, Agusti S, Barbier E, Britten GL, Castilla JC, Gattuso JP et al. Rebuilding marine life. Nature. 2020; 580 (7801): 39-51.

9. Stevens JD, Bonfil R, Dulvy NK, Walker PA. The effects of fishing on sharks, rays, and chimaeras (chondrichthyans), and the implications for marine ecosystems. ICES J Mar Sci. 2000; 57 (3): 476-494.
10. Shin YJ, Rochet MJ, Jennings S, Field JG, Gislason H. Using size-based indicators to evaluate the ecosystem effects of fishing. ICES J Mar Sci. 2005; 62 (3): 384-396.
11. Coll M, Carreras M, Ciércoles C, Cornax MJ, Gorelli G, Morote E, et al. Assessing fishing and marine biodiversity changes using fishers' perceptions: the Spanish Mediterranean and Gulf of Cadiz case study. PLoS One. 2014; 9 (1): e85670.
12. Peñaherrera-Palma C, Putten IV, Karpievitch YV, Frusher S, Llerena-Martillo Y, Hearn AR, et al. Evaluating abundance trends of iconic species using local ecological knowledge. Biol Conserv. 2018; 225, 197-207.
13. Shackell NL, Frank KT, Fisher JA, Petrie B, Leggett WC. Decline in top predator body size and changing climate alter trophic structure in an oceanic ecosystem. Proc Royal Soc B. 2010; 277 (1686): 1353-1360.
14. Heithaus MR, Frid A, Wirsing AJ, Worm B. Predicting ecological consequences of marine top predator declines. Trends Ecol Evol. 2008; 23 (4): 202-210.
15. McClenachan L. Historical declines in goliath grouper in south Florida. Endang Species Res. 2009; 7 (3): 175-181.
16. Venkatachalam AJ, Price ARG, Chandrasekara S, Sellamuttu SS, Kaler J. Changes in frigate tuna populations on the south coast of Sri Lanka: evidence of the shifting baseline syndrome from analysis of fisher observations. Aquat Conserv Mar Freshwater Ecosyst. 2010; 20 (2): 167-176.
17. Luiz OJ, Edwards AJ. Extinction of a shark population in the Archipelago of Saint Paul’s Rocks (equatorial Atlantic) inferred from the historical record. Biol Conserv. 2011; 144 (12): 2873-2881.

18. Giglio VJ, Luiz OJ, Gerhardinger LC. Depletion of marine megafauna and shifting baselines among artisanal fishers in eastern Brazil. Anim Conserv. 2015; 18 (4): 348-358.
19. Giglio VJ, Bender MG, Zapelini C, Ferreira CEL. The end of the line? Rapid depletion of a large-sized grouper through spearfishing in a subtropical marginal reef. Perspect Ecol Conserv. 2017; 15 (2): 115-118.
20. Lewison RL, Crowder LB, Read AJ, Freeman SA. Understanding impacts of fisheries bycatch on marine megafauna. Trends Ecol Evol. 2004; 19 (11): 598-604.
21. Myers RA, Baum JK, Shepherd TD, Powers SP, Peterson CH. Cascading Effects of the Loss of Apex Predatory Sharks from a Coastal Ocean. Science. 2007; 315 (5820): 1846-1850.
22. Baum JK, Worm B. Cascading top down effects of changing oceanic predator abundances. J Animal Ecol. 2009; 78 (4): 699-714.
23. Essington TE, Beaudreau AH, Wiedenmann J. Fishing through marine food webs. Proc Natl Acad Sci USA. 2006; 103 (9): 3171-3175.
24. Heithaus MR, Frid A, Vaudo JJ, Worm B, Wirsing AJ 2010. Unraveling the ecological importance of elasmobranchs. In: Carrier JC, Musick J, Heithaus MR (eds) Biology of Sharks and their relatives II. CRC Press, Boca Raton. 611-637.
25. Pauly D, Christensen V, Dalsgaard J, Froese R, Torres Jr F. Fishing down marine food webs. Science. 1998; 279 (5352): 860-863.
26. Rogers SI, Ellis JR. Changes in the demersal fish assemblages of British coastal waters during the 20th century. ICES J Mar Sci. 2000; 57 (4): 866-881.
27. Molfese C, Beare D, Hall-Spencer JM. Overfishing and the Replacement of Demersal Finfish by Shellfish: An Example from the English Channel. PLoS One. 2014; 9 (7): e101506.

28. Pauly D, Palomares ML, Froese R, Sa-a P, Vakily M, Preikshot D, et al. Fishing down Canadian aquatic food webs. Can J Fish Aquat Sci. 2001; 58 (1): 51-62.
29. Pauly D, Watson R. Background and interpretation of the 'Marine Trophic Index' as a measure of biodiversity. Philos Trans R Soc B. 2005; 360 (1454): 415-423.
30. Freire KMF, Pauly D. Fishing down Brazilian marine food webs, with emphasis on the east Brazil large marine ecosystem. Fish Res. 2010; 105 (1): 57-62.
31. Liang C, Pauly D. Fisheries impacts on China's coastal ecosystems: Unmasking a pervasive 'fishing down' effect. PLoS One. 2017; 12 (3): e0173296.
32. Erlandson JM, Rick TC, Braje TJ. Fishing up the Food Web?: 12,000 Years of Maritime Subsistence and Adaptive Adjustments on California's Channel Islands. Pac Sci. 2009; 63 (4): 711-724.
33. Litzow MA, Urban D. Fishing through (and up) Alaskan food webs. Can J Fish Aquat Sci. 2009; 66 (2): 201-211.
34. Branch TA. Fishing Impacts on Food Webs: Multiple Working Hypotheses. Fisheries. 2015; 40 (8): 373-375.
35. Salas S, Chuenpagdee R, Seijo JC, Charles A. Challenges in the assessment and management of small-scale fisheries in Latin America and the Caribbean. Fish Res. 2007; 87 (1): 5-16.
36. Begossi A, Silvano RAM. Ecology and ethnoecology of dusky grouper [garoupa, *Epinephelus marginatus* (Lowe, 1834)] along the coast of Brazil. J Ethnobiol Ethnomed. 2008; 4 (1): 20.
37. Lima EG, Begossi A, Hallwass G, Silvano RAM. Fishers' knowledge indicates short-term temporal changes in the amount and composition of catches in the southwestern Atlantic. Mar Policy. 2016; 71, 111-120.

38. Sáenz-Arroyo A, Roberts CM, Torre J, Cariño-Olvera M, Hawkins JP. The value of evidence about past abundance: marine fauna of the Gulf of California through the eyes of 16th to 19th century travellers. Fish Fish. 2006; 7 (2): 128-146.
39. Martins IM, Medeiros RP, Domenico MD, Hanazaki N. What fishers' local ecological knowledge can reveal about the changes in exploited fish catches. Fish Res. 2018; 198, 109-116.
40. Giglio VJ, Suhett AC, Zapelini CS, Ramiro AS, Quimbayo JP. Assessing captures of recreational spearfishing in Abrolhos reefs, Brazil, through social media. Reg Stud Mar Sci. 2020; 34, 100995.
41. Braga HO, Azeiteiro UM, Oliveira HMF, Pardal MA. Conserving Brazilian Sardine: Fisher's attitudes and knowledge in the Marine Extractive Reserve of Arraial do Cabo, Rio de Janeiro State, Brazil. Fish Res. 2018; 204, 402-411.
42. Taylor SM, Braccini JM, Bruce BD, McAuley RB. Reconstructing Western Australian white shark (*Carcharodon carcharias*) catches based on interviews with fishers. Mar Freshwater Res. 2018; 69 (3): 366-375.
43. Baum JK, Myers RA. Shifting baselines and the decline of pelagic sharks in the Gulf of Mexico. Ecol Lett. 2004; 7 (2): 135-145.
44. Soga M, Gaston KJ. Shifting baseline syndrome: causes, consequences, and implications. Front Ecol Environ. 2018; 16 (4): 222-230.
45. Pauly D. Anecdotes and the shifting baseline syndrome of fisheries. Trends Ecol Evol. 1995; 10 (10): 430.
46. Papworth SK, Coad JRL, Milner-Gulland EJ. Evidence for shifting baseline syndrome in conservation. Conserv Lett. 2009; 2 (2): 93-100.

47. Thurstan RH, McClenachan L, Crowder LB, Drew JA, Kittinger JN, Levin PS. Filling historical data gaps to foster solutions in marine conservation. Ocean Coastal Manage. 2015; 115, 31-40.

48. Bender MG, Floeter SR, Hanazaki N. Do traditional fishers recognise reef fish species declines? Shifting environmental baselines in Eastern Brazil. Fish Manag Ecol. 2013; 20 (1): 58-67.

49. Bender MG, Machado GR, Azevedo PJS, Floeter SR, Monteiro-Netto C, Luiz OJ, et al. Local Ecological Knowledge and Scientific Data Reveal Overexploitation by Multigear Artisanal Fisheries in the Southwestern Atlantic. PloS One. 2014; 9 (10): e110332.

50. Reis-Filho JA, Freitas RHA, Loiola M, Leite L, Soeiro G, Oliveira HHQ, et al. Traditional fisher perceptions on the regional disappearance of the largetooth sawfish *Pristis pristis* from the central coast of Brazil. Endang Species Res. 2016; 29 (3): 189-200.

51. Silvano RAM, MacCord PFL, Lima RV, Begossi A. When Does this Fish Spawn? Fishermen’s Local Knowledge of Migration and Reproduction of Brazilian Coastal Fishes. Environ Biol Fishes. 2006; 76 (2-4): 371-386.

52. Silvano RAM, Begossi A. Fishermen's local ecological knowledge on Southeastern Brazilian coastal fishes: contributions to research, conservation, and management. Neotr Ichthyol. 2012; 10 (1): 133-147.

53. Barbosa-Filho MLV, Schiavetti A, Alarcon DT, Costa-Neto EM. “Shark is the man!”: ethnoknowledge of Brazil’s South Bahia fishermen regarding shark behaviors. J Ethnobiol Ethnomed. 2014; 10 (1): 54.

54. Herbst DF, Hanazaki N. Local ecological knowledge of fishers about the life cycle and temporal patterns in the migration of mullet (Mugil liza) in Southern Brazil. Neotr Ichthyol. 2014; 12 (4): 879-890.

55. Sáenz-Arroyo A, Roberts CM, Torres J, Cariño-Olvera M. Using fishers' anecdotes, naturalists' observations and grey literature to reassess marine species at risk: the case of the Gulf grouper in the Gulf of California, Mexico. Fish Fish. 2005; 6 (2): 121-133.

56. Gerhardinger LC, Godoy EAS, Jones PJS. Local ecological knowledge and the management of marine protected areas in Brazil. Ocean Coast Manage. 2009; 52 (3-4): 154-165.

57. Wiber MG, Young S, Wilson L. Impact of Aquaculture on Commercial Fisheries: Fishermen's Local Ecological Knowledge. Hum Ecol Interdiscip J. 2012; 40 (1): 29-40.

58. Damasio LDMA, Lopes PFM, Guariento RD, Carvalho AR. Matching Fishers' Knowledge and Landing Data to Overcome Data Missing in Small-Scale Fisheries. PLoS One. 2015; 10 (7): e0133122.

59. Bevilacqua AHV, Carvalho AR, Angelini R, Christensen V. More than Anecdotes: Fishers' Ecological Knowledge Can Fill Gaps for Ecosystem Modeling. PLoS One. 2016; 11 (5): e0155655.

60. Aylesworth L, Phoonsawat R, Suvanachai P, Vincent ACJ. Gaenerating spatial data for marine conservation and management. Biodivers Conserv. 2017; 26 (2): 383-399.

61. Santos RO, Rehage JS, Kroloff EKN, Heinen JE, Adams AJ. Combining data sources to elucidate spatial patterns in recreational catch and effort: fisheries-

dependent data and local ecological knowledge applied to the South Florida bonefish fishery. Environ Biol Fishes. 2019; 102 (2): 299-317.

62. Melo JCF, Kruel VSF, Hanazaki N. Árvores e madeiras na cultura naval tradicional Bailey, KD. 1994. Methods of Social Research. New York: The Free Press/Macmillan Publishers. 588; 2019.

63. Carvalho WFD, Gonzalez Rodriguez E. Development of primary and bacterial productivity in upwelling waters of Arraial do Cabo region, RJ (Brazil). Braz J Oceanogr. 2004; 52 (1): 35-45.

64. Cordeiro CAMM, Mendes TC, Harborne AR, Ferreira CEL. Spatial distribution of nominally herbivorous fishes across environmental gradients on Brazilian rocky reefs. J Fish Biol. 2016; 89 (1): 939-958.

65. Bailey, KD, 1994. Methods of Social Research. New York: The Free Press/Macmillan Publishers. 588.

66. Maia HA, Morais RA, Siqueira AC, Hanazaki N, Floeter SR, Bender MG. Shifting baselines among traditional fishers in São Tomé and Príncipe islands. Ocean Coast Manage. 2018; 154, 133-142.

67. Pauly D, Froese R. Comments on FAO's State of Fisheries and Aquaculture, or ‘SOFIA 2010’. Mar Policy. 2012; 36 (3): 746-752.

68. Palomares MLD, Pauly D. Editors. 2020. SeaLifeBase. World Wide Web electronic publication. Available from: www.sealifebase.org, version (07/2020).

69. Pauly D, Christensen V, Guénette S, Pitcher TJ, Sumaila UR, Walters CJ, et al. Towards sustainability in world fisheries. Nature. 2002; 418 (6898): 689-695.

70. R Development Core Team. 2019. R: A language and environment for statistical computing. 418 Vienna, Austria: R Foundation for statistical Computing.

71. Lovell S, Johnson AE, Ramdeen R, McClenachan L. Shifted baselines and the policy placebo effect in conservation. Oryx. 2018; 54 (3): 383-391.

72. Begossi A, Salivonchyk SV, Barreto T, Nora V, Silvano RAM. Small-scale Fisheries and Conservation of Dusky Grouper (Garoupa), *Epinephelus marginatus* (Lowe, 1834) in the Southeastern Brazilian Coast. Sci J Agric Res Manag. 2012; 2012:4.

73. Ferreira HM, Reuss-Strenzel GM, Alves JA, Schiavetti A. Local ecological knowledge of the artisanal fishers on *Epinephelus itajara* (Lichtenstein, 1822) (Teleostei: Epinephelidae) on Ilhéus coast-Bahia State, Brazil. J Ethnobiol Ethnomed. 2014; 10 (1): 51.

74. Seyboth E, Condini MV, Albuquerque CQ, Varela Jr AS, Velasco G, Vieira JP, et al. Age, growth, and reproductive aspects of the dusky grouper *Mycteroperca marginata* (Actinopterygii: Epinephelidae) in a man-made rocky habitat in southern Brazil. Neotrop Ichthyol. 2011; 9 (4): 849-856.

75. Condini MV, García-Charton JA, Garcia AM. A review of the biology, ecology, behavior and conservation status of the dusky grouper, *Epinephelus marginatus* (Lowe 1834). Rev Fish Biol Fish. 2018; 28 (2): 301-330.

76. Sadovy de Mitcheson Y, Craig MT, Bertoncini AA, Carpenter KE, Cheung WWL, Choat JH, et al. Fishing groupers towards extinction: a global assessment of threats and extinction risks in a billion dollar fishery. Fish Fish. 2013; 14(2): 119-136.

77. Duarte MR, Tubino RA, Monteiro-Neto C, Martins RRM, Vieira FC, Andrade-Tubino MF et al. Genetic and morphometric evidence that the jacks (Carangidae) fished off the coast of Rio de Janeiro (Brazil) comprise four different species. Biochem Syst Ecol. 2017; 71, 78-86.

78. Ceretta BF, Fogliarini CO, Giglio VJ, Maxwell MF, Waechter LS, Bender MG. Testing the accuracy of biological attributes in predicting extinction risk. Perspect Ecol Conserv. 2020; 18 (1): 12-18.
79. Stergiou KI, Koulouris M. "Fishing down the marine food webs in the Hellenic seas." Fishing down the Mediterranean food webs. 2000; 26, 30.
80. Bhathal B, Pauly D. ‘Fishing down marine food webs’ and spatial expansion of coastal fisheries in India, 1950-2000. Fish Res. 2008; 91 (1): 26-34.
81. Ding Q, Chen X, Yu W, Tian S, Chen Y. An evaluation of underlying mechanisms for “fishing down marine food webs”. Acta Oceanol Sin. 2016; 35 (8): 32-38.
82. Blanchard JL, Coll M, Trenkel VM, Vergnon R, Yemane D, Jouffre D, et al. Trend analysis of indicators: a comparison of recent changes in the status of marine ecosystems around the world. ICES J Mar Sci. 2010; 67 (4): 732-744.
83. Coll M, Shannon LJ, Yemane D, Link JS, Ojaveer H, Neira S, et al. Ranking the ecological relative status of exploited marine ecosystems. ICES J Mar Sci. 2010; 67 (4): 769-786.
84. Alleway HK, Connell SD, Ward TM, Gillanders BM. Historical changes in mean trophic level of southern Australian fisheries. Mar Freshwater Res. 2014; 65 (10): 884-893.
85. Stergiou KI, Tsikliras AC. Fishing down, fishing through and fishing up: fundamental process versus technical details. Mar Ecol Prog Ser. 2011; 441, 295-301.
86. Liang C, Pauly D. Masking and unmasking fishing down effects: The Bohai Sea (China) as a case study. Ocean Coast Manag 2020; 184, 105033.
87. Branch TA, Watson R, Fulton EA, Jennings S, McGilliard CR, Pablico GT, et al. The trophic fingerprint of marine fisheries. Nature, 2010; 468 (7322): 431-435.

88. Foley CMR. Management implications of fishing up, down, or through the marine food web. Mar Policy. 2013; 37: 176–182.
89. Francini-Filho RB, De Moura RL. Dynamics of fish assemblages on coral reefs subjected to different management regimes in the Abrolhos Bank, eastern Brazil. Aquat Conserv Mar Freshwater Ecosyst. 2008; 18 (7): 1166.
90. Roos NC, Pennino MG, De Macedo Lopes PF, Carvalho AR. Multiple management strategies to control selectivity on parrotfishes harvesting. Ocean Coast Manage. 2016; 134, 20-29.
91. Floeter SR, Halpern BS, Ferreira CEL. Effects of fishing and protection on Brazilian reef fishes. Biol Conserv. 2006; 128 (3): 391-402.
92. Fouzai N, Coll M, Palomera I, Santojanni A, Arneri E, Christensen V. Fishing management scenarios to rebuild exploited resources and ecosystems of the Northern-Central Adriatic (Mediterranean Sea). J Mar Syst. 2012; 102, 39-51.
93. Paterson B. Integrating fisher knowledge and scientific assessments. Anim Conserv. 2010; 13 (6): 536-537.

Supporting Information

S1 Table. Trophic level (TL) of species landed Arraial do Cabo, from 1992 to 2008. Trophic levels were obtained from FishBase where a higher value means higher trophic levels (Froese and Pauly, 2019).

S2 Table. Fishing effort of the artisanal fishing fleet of Arraial do Cabo, Rio de Janeiro, Brazil.

S1 Fig. Differences in mean trophic level (MTL) of overexploited and new target species cited by local fishers. Observed MTL (red diamond) are contrasted against null

MTL values (gray circles), generated through randomization of citation frequency of species within each category.

S2 Fig. Fishing effort in number of boats (a) and fishing days (b) over the years for Arraial do Cabo, Rio de Janeiro, Brazil.

S1 File. Questionnaire applied in interviews with fishers in Arraial do Cabo.

 Click here to access/download;Figure;Fig 1.tiff.tif

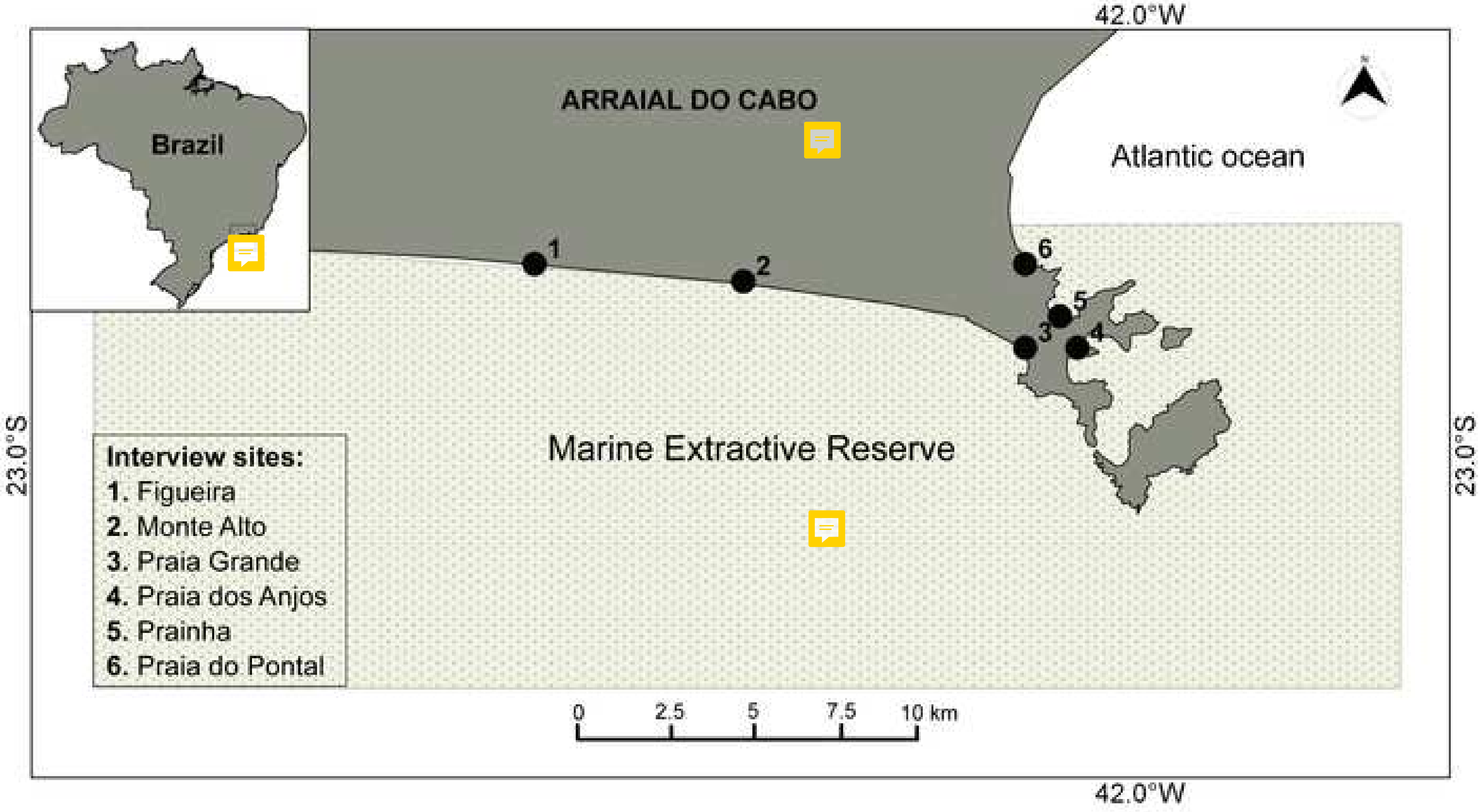

Figure

Click here to access/download;Figure;Fig 2.tiff.tif

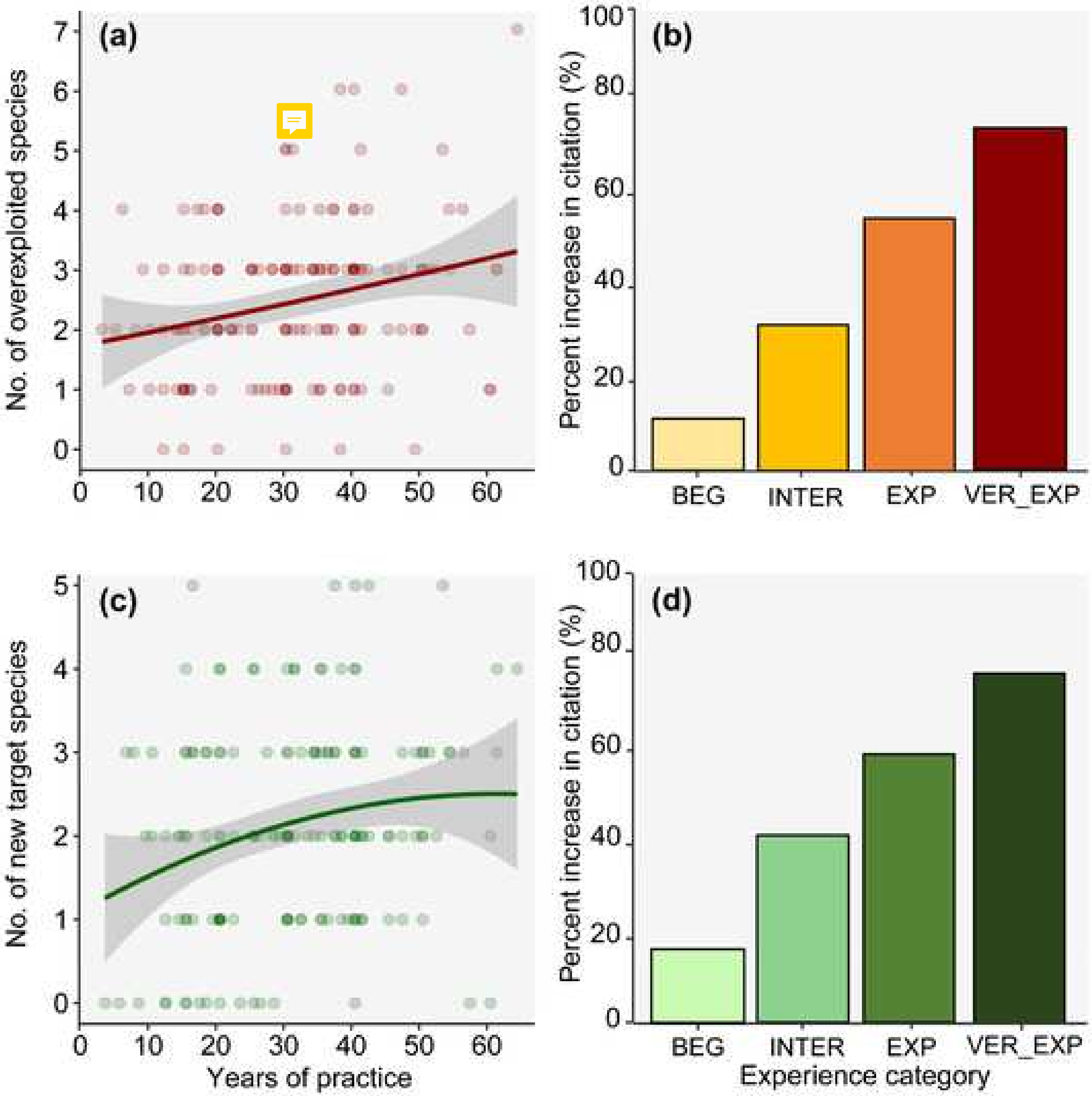

Figure

Click here to access/download;Figure;Fig 3.tiff.tif

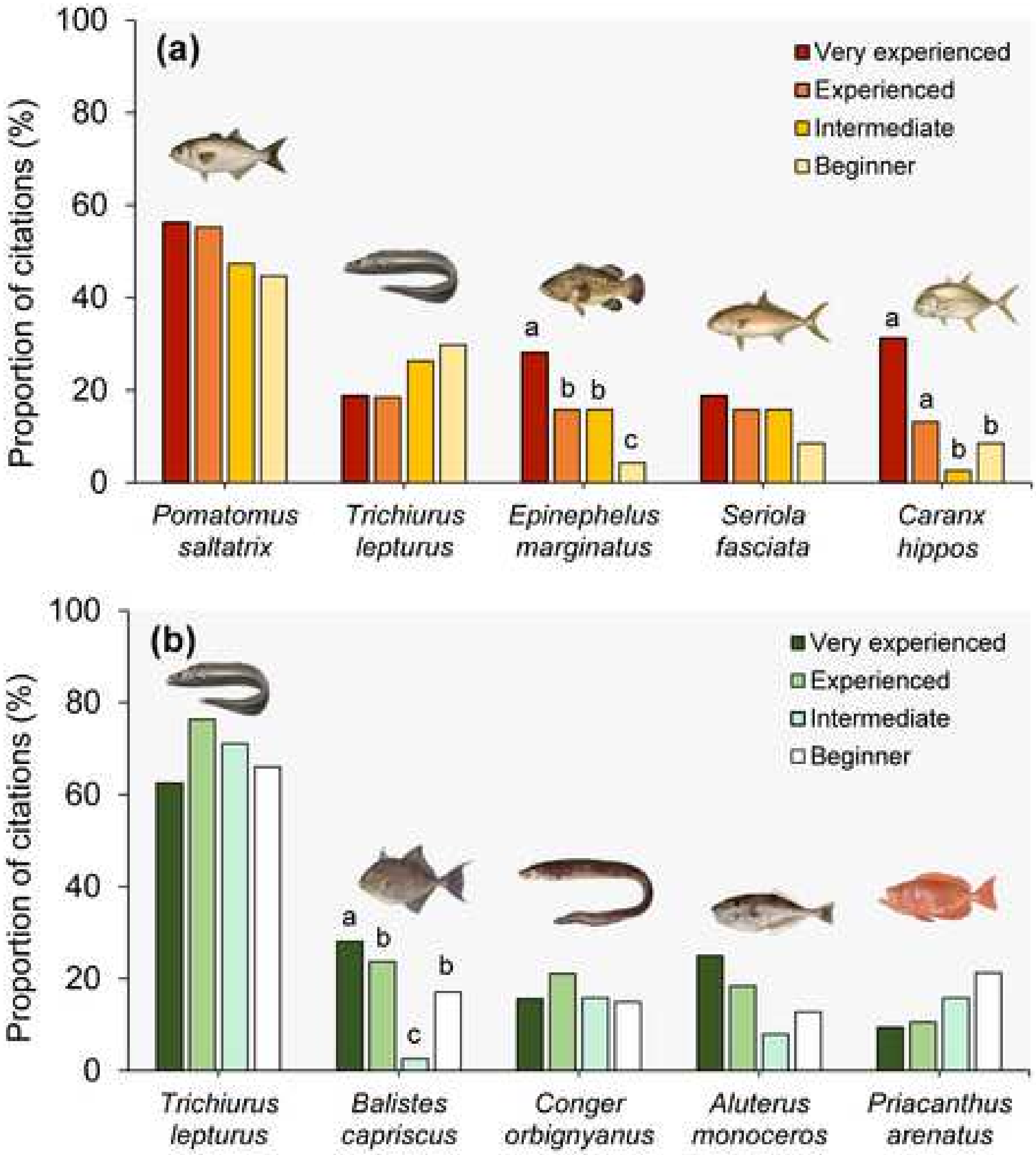

Click here to access/download;Figure;Fig 4.tiff.tif

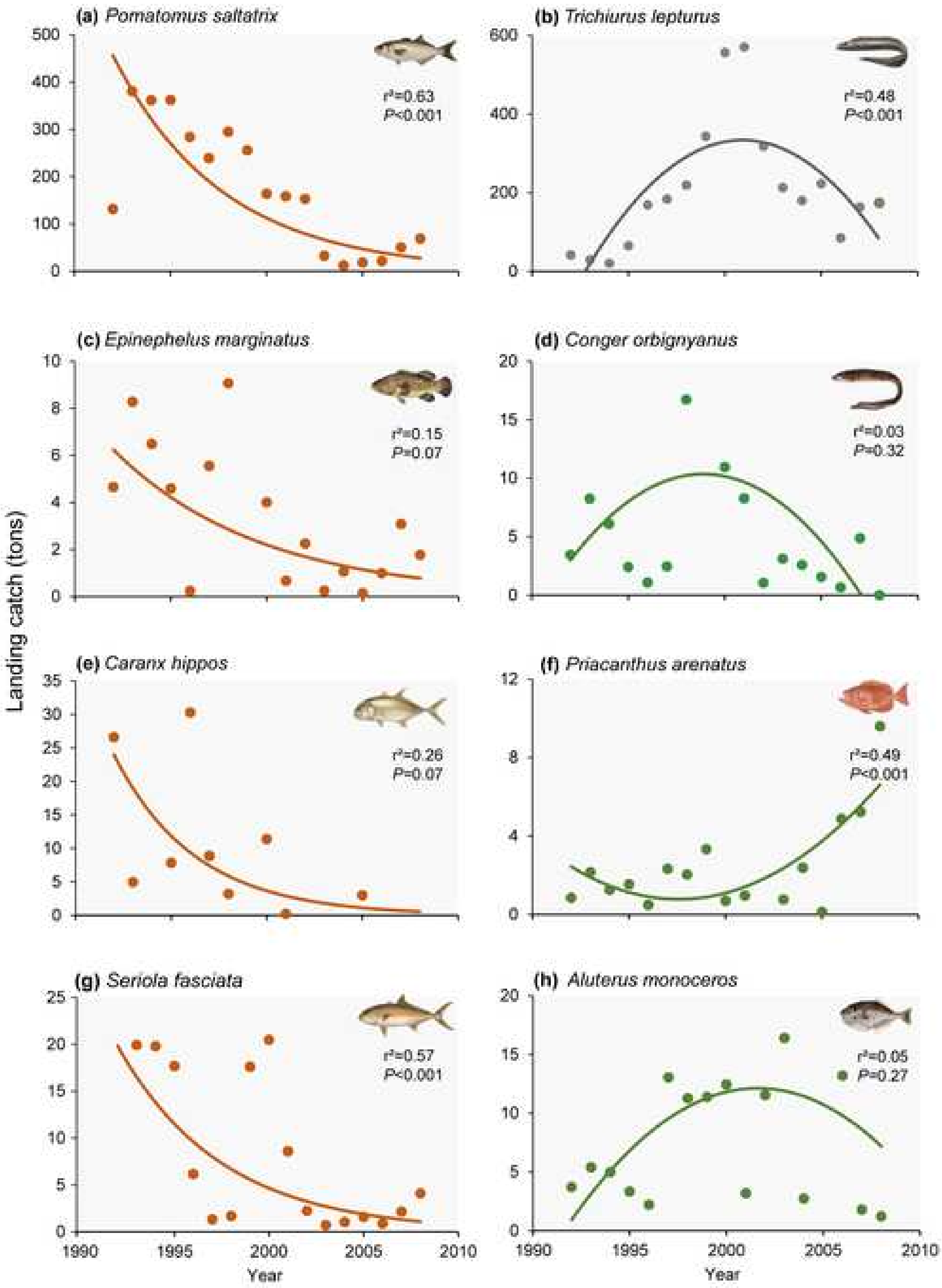

Click here to access/download;Figure;Fig 5.tiff.tif

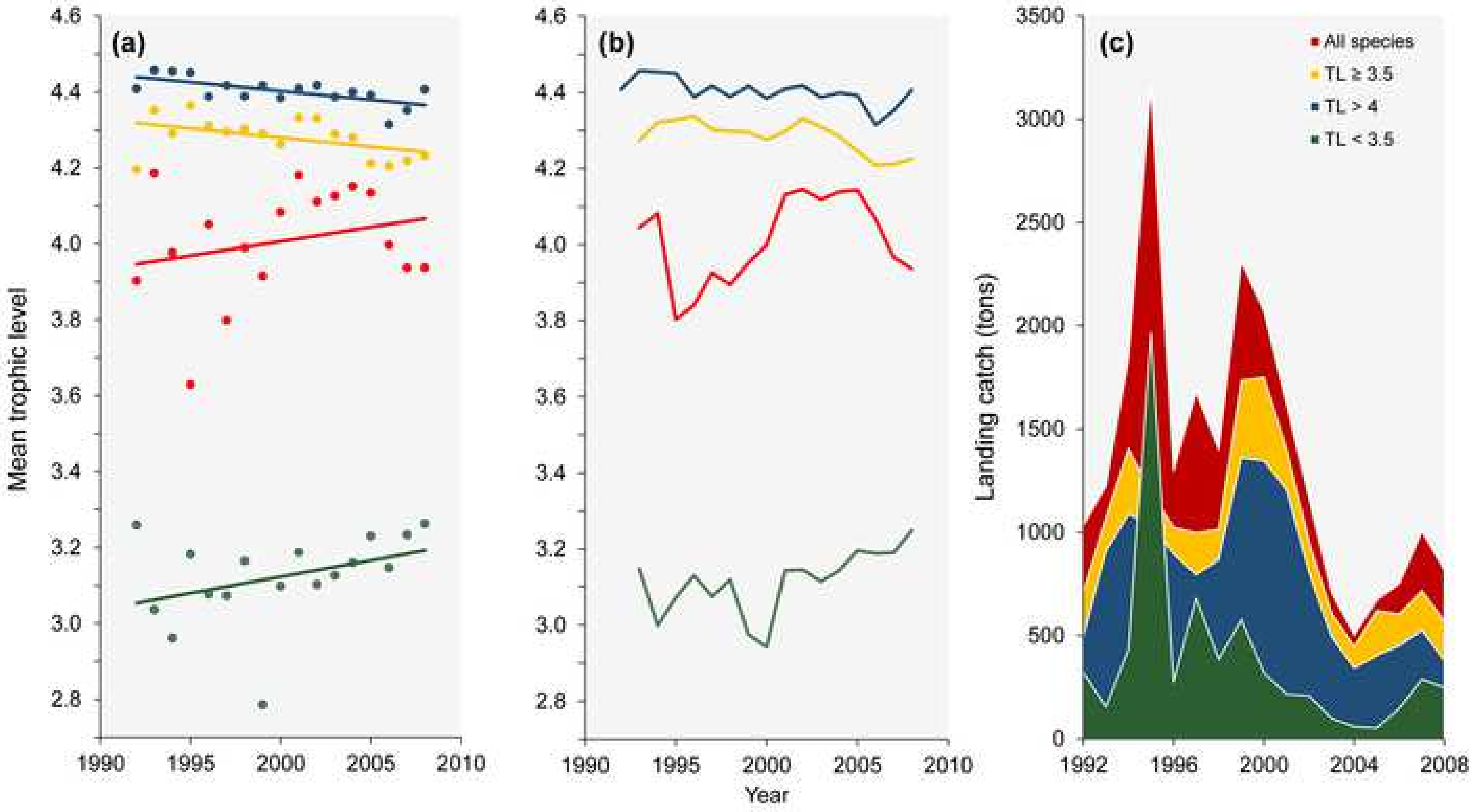

Figure Click here to access/download;Figure;Fig 6.tiff.tif

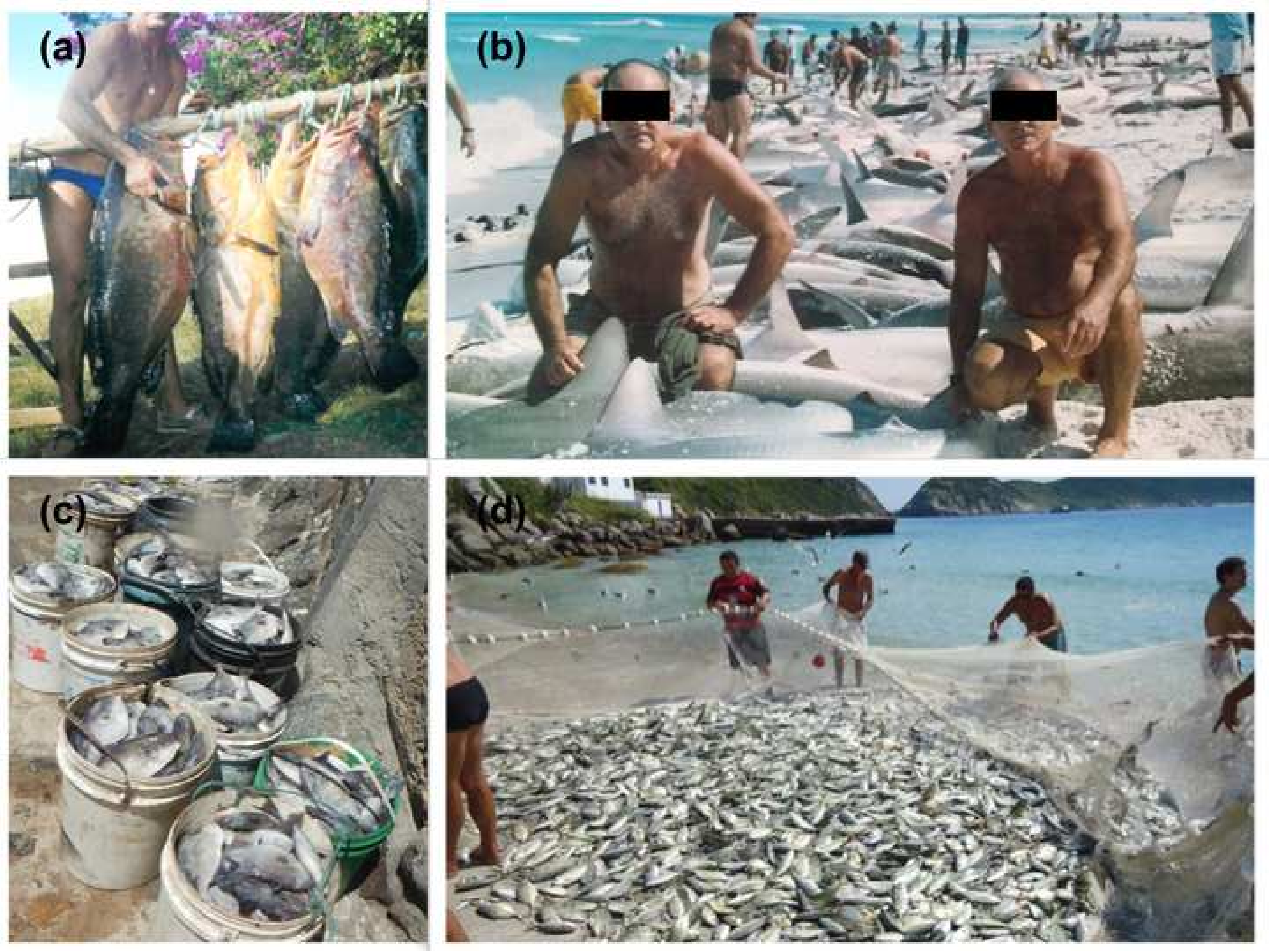

Click here to access/download
Supporting Information
Supporting information_Plos One.docx
