# Supplementary material for: Telling the same story: Fishers and landing data reveal changes in fisheries on the Southeastern Brazilian Coast
Source: PLoS One. 2021 Jun 1;16(6):e0252391. doi: 10.1371/journal.pone.0252391 (PMC8168859; doi:10.1371/journal.pone.0252391)
Supplement: Attachment — Submitted filename: PONE-D-20-30952_R1 - final.pdf [file pone.0252391.s004.pdf]

PLOS ONE

Telling the same story: fishers and landing data reveal changes in fisheries on the Southeastern Brazilian coast

--Manuscript Draft--

**Manuscript Number:**	PONE-D-20-30952R1
**Article Type:**	Research Article
**Full Title:**	Telling the same story: fishers and landing data reveal changes in fisheries on the Southeastern Brazilian coast
**Short Title:**	Fishers and landing data reveal changes in fisheries on the Southeastern Brazilian coast
**Corresponding Author:**	Carine de Oliveira, M.D<br>Universidade Federal de Santa Maria<br>Santa Maria, Rio Grande do Sul BRAZIL
**Keywords:**	Shifting baseline; marine resources; overfishing; local ecological knowledge; Fisheries monitoring; Fisheries Management
**Abstract:**	An understanding of the effects of fishing on marine ecosystems relies on information about the conserved state of these environments. Non-conventional approaches such as the use of historical data and local ecological knowledge can provide information and help adjust our references of changes in the environment. Also, the combination of different types of data can indicate a fisheries trend that would be undetectable when evaluated separately. Here we investigated changes in fisher’s perceptions regarding overexploited and new target species in artisanal fisheries in a secular fishing village of the subtropical, southeastern Brazilian coast. We identified temporal changes in landings and in the mean trophic level (MTL) of high trophic level species (≥ 3.5 and >4) over 16 years. Fishers' knowledge revealed shifts in perception associated with years of fishing practice. More experienced fishers recognized a greater number of overexploited and new target species than fishers in the beginning of their careers. Landing data has revealed declining trends of 72% for five mesopredators species. Due to the overfishing of mesopredators, there was a shift in target species, towards fish that were previously discarded. Temporal changes in landings and in the MTL metric are concordant with previous reports on the overexploitation of species caught by local fishers. Our work reveals that multiple sources of information can be combined to establish historical baselines and improve the detection of change in marine ecosystems.
**Order of Authors:**	Carine O. Fogliarini, M.D
	Carlos E.L. Ferreira
	Jéssica Bornholdt
	Moysés. C. Barbosa
	Vinicius J. Giglio
	Mariana G. Bender
**Response to Reviewers:**	Response to Reviewers<br><br>Reviewer #1: The manuscript presents a careful analysis and data, although several of them do not present relations as convincing as suggested in the text. However, the manuscript presents so much results and approaches without any novelty for an international audience. It is shown by data from formal science and traditional knowledge that fisheries initially reach the highest trophic levels, that perceptions change over time, that fishing targets are being changed. This is the description of a historical process of any artisanal fisheries in the world. It is not clear what are the innovative aspects in the article that would justify its publication. Considering these aspects, I cannot recommend the publication of the article in a journal such as PLOS ONE<br>R. We respect Reviewer #1 for his opinion and understand that this is a common

Powered by Editorial Manager® and ProduXion Manager® from Aries Systems Corporation

pattern reported for artisanal fisheries across the world. Yet there are few studies in which Local Ecological Knowledge, from traditional communities, has been contrasted with landing data to reveal matching patterns in local fisheries (Silvano et al. 2009, Ferreira et al. 2014, Herbst & Hanazaki, 2014, Silva et al. 2020). Considering this aspect, at the best of our knowledge, the present study is the first to assess exploitation status of fish species caught by artisanal fishing and changes in target species over time in the southwestern Atlantic. We highlight that the study area is a partially-protected marine area, whose main resource managers are the extractive community. Here, we combine the knowledge of fishers with landings data, to understand the dynamics of fish stocks from the past to the present. Thus, we generate information that can be used to propose conservation and management measures. We point out that there is no information of temporal changes in fishery targets along Brazilian coast. Such information is not only of ecological importance, but also cultural and economic. Also, there are several examples of local scale studies published in PLOS ONE, that are not necessarily of broad interest
(ttps://journals.plos.org/plosone/article?id=10.1371/journal.pone.0236146)
(https://journals.plos.org/plosone/article?id=10.1371/journal.pone.0228976)

Reviewer #2: Review of de Oliveira et al. Fishing down the food web inferred from local ecological knowledge and landing data in the Southeastern Brazilian coast
De Oliveira et al used traditional ecological knowledge from fishers in a coastal community of subtropical Brazil (Arraial do Cabo) and historical landing statistics to identify species overexploitation patterns, changes in target fishery resources and trophic levels. Using information from 155 interviews made to fishers and the landing statistics from that area during 1992 and 2008, the authors tried to identify which species according to the perception of fishers were overexploited and which species were new components of the fishery. Additionally, the authors calculated the mean trophic level of the annual landings in that area to verify if the fishing down the food web hypothesis would hold true for that area.
There is a vast amount of literature that has combined TEK with conventional fisheries information to complement our knowledge of a specific fishery. The present study is one of those studies and is a welcome contribution to this relatively new field. I am more critical about how the authors have tried to show their results especially with respect to the fishing down the food web hypothesis and in a lesser degree to the shifting baseline paradigm.
My impression from the data shown is that there is no reason to believe that there is a fishing down the food web pattern in the study system, at least with the information (MTL) that the authors are analyzing. Despite there are a lot of studies that have pointed to a shifting baseline situation in fisheries, where younger fishers won’t recognize easily the degraded state of fishery resources, the present study in my opinion does not show that clear pattern (by inspecting Fig 2a,c). This is totally fine as a result and there is no reason to try to adjust the argument to the prevailing paradigm.In my opinion, the most interesting finding of the paper is the change in target species pointed out by fishers. This is something that could be highlighted much more in the manuscript. Discussion of the implications of this with similar results in other parts of the world would make the manuscript much stronger.
Having said this, I welcome this contribution to the field, but I disagree with the main interpretation of the data presented in the manuscript. I therefore recommend the authors to reconsider many of the interpretations of their findings in light of what their actual data show. After these major criticisms are addressed I think that this manuscript is not suitable for publication in a PloS ONE.
See specific comments below.
R: We agree that the fishing down the food web process was not detected when we analyzed the MTL of all landed species. However, this process may not be easily detected due to the influence of factors that may mask its occurrence. Some factors that may mask fishing down are taxonomic over aggregation of catch statistics and the spatial expansion of fishing, but that can be clarified by local experts. Also, the masking effect can occur when both the proportion of high TL species and pelagic species of low TL are declining. In our case, we identified declines in landings of the two main landed species, largehead hairtail (Trichiurus lepturus) and the sardine (Sardinella brasiliensis) (see figures below). Landings of T. lepturus, a high TL species, had an increase from the 1990s to 2000s, followed by a decrease in landings after the 2000s. The same decline trend was identified for the sardine, the main species landed in the region. Declining trends in landings of these species are possibly causing an increase

in the MTL of all species, as demonstrated in other studies (Blanchard et al. 2010, Coll et al. 2010). In this case, the masking effect is named as “false fishing up” and we discussed the possible occurrence of this process in our study (lines 440 - 447). Another important result that suggests the overexploitation scenario was showed through landings of high TL species (TL> 4) that decreased significantly over time (Fig. 5c, d). For some stocks, the reduction exceeded 80% (Fig. 4a, c, e, g). In addition, we asked fishers about the status of fishing stocks, and the answers to this question were not added to our manuscript, but it is important to highlight that 92% of fishers reported a decline in fishing stocks, and most of them reported that fish stocks decrease from 50 to 80% (see figure below). Finally, the overexploitation scenario of marine resources has been supported by another local study (Bender et al. 2014) based on fisher’s knowledge, underwater visual census and scientific data. The authors also identified declines in high and low TL species caught by artisanal fisheries.

With respect to the shifting baseline results, we understand the reason for his argument. The distribution of the number of citations does not vary so much between years of fishing practice (fig. 2 a, c). However, we showed that there were different percent increase in citations as fishers’ experience increase (Fig. 2 b, d). Another evidence of the shifting baseline occurs when there are significant differences in the proportion of citations between experience categories (Fig. 3). Very experienced fishers identified Epinephelus marginatus, Caranx hippos, and Balistes capriscus as overexploited and new target species more frequently than the less experienced ones (Fig. 3).

Abstract
L36. Clarify is you are referring to landings or to catches. These are two different concepts. Look at differences here: http://www.fao.org/3/bt981t/bt981t.pdf
R: Thank you for your comment. We changed this expression along to manuscript. In our case, the correct term is "landings" because it represents the quantities landed as recorded at the time of landing as whole or eviscerated fish, fillets, livers, roes, etc.

L55. Provide a more specific sentence on the problem of sustainability in fisheries. The recent FAO SOFIA reports clearly define and provide a historical perspective on the percentage of fish stocks that are fished at biologically sustainable levels since the 70s.
R: We added the following sentence: “The status assessment of fishery resources revealed that the fraction of fish stocks that are within biologically sustainable levels decreased from 90% in 1974 to 65.8% in 2017 (FAO, 2020).”

L61-62. Not all fisheries are directed toward top predators. This is a misleading statement. Modify sentence or bring clarification to what you actually mean.
R: We agree with your statement and modified the sentence (Lines 62 - 65).

L71. The “fishing down the food web“ paradigm has been continuously revised since it was first proposed. I would suggest that the authors consider at least mentioning in the introduction the critics of the paradigm. For this, I would recommend to revise Branch et al. (2010) The trophic fingerprint of marine fisheries. Nature 468, 431–435 and Sethi et al (2010) Global fishery development patterns are driven by profit but not trophic level. PNAS 107, 12163-12167.
R: We have included the following explanation: “In addition, studies criticize the exclusive use of MTL as an indicator to track changes in the ecosystem (Branch et al. 2010). As well as there may be multiple hypotheses (Branch et al. 2015), which explain how fishing affects marine food webs, such as profit-driven fishing (Sethi et al. 2010).”

L73. Change “trophic” for “food”.
R: The term was replaced. (Line 74).

L72-79. See comment from L71. Maybe rephrasing the whole paragraph reflecting that there are many ways how fishing can affect marine ecosystems (i.e. not only by reducing trophic level), would be the way to go.
R: We agreed and added an explanation in lines 73 - 84.

L93. Do you mean unexplored or unexploited?

R: We mean "unexplored state". We clarified it in the text.

L109. Change "have" for "has"
R: Replaced accordingly.

L109-114. A number of recent studies have used LEK to link to trophic models. This application may be worth including here and probably somewhere in the discussion. Just a few examples:
Bevilacqua AHV, Carvalho AR, Angelini R, Christensen V (2016) More than Anecdotes: Fishers' Ecological Knowledge Can Fill Gaps for Ecosystem Modeling. PLoS ONE 11(5): e0155655. https://doi.org/10.1371/journal.pone.0155655
Sánchez-Jiménez A, Fujitani M, MacMillan D, Schlüter A and Wolff M (2019) Connecting a Trophic Model and Local Ecological Knowledge to Improve Fisheries Management: The Case of Gulf of Nicoya, Costa Rica. Front. Mar. Sci. 6:126. doi: 10.3389/fmars.2019.00126
R: We added these studies in lines 112 - 116.

L120. Can you specify what is the MTL approach?
R. Done. Lines 123 - 124.

Also, I am not sure if you can link possible changes in landings trophic structure exclusively to fishing impacts. There may be other reasons associated these to changes. Provide some clarification on how are you able to assign the possible changes just to fishing.
R: First, we observed through information from fishers that a large part of the resources identified as overexploited were high trophic level species. This finding motivated us to try to understand how fishing is affecting the local ecosystem. Second, the temporal replacement of target species caught our attention –as the case of largehead hairtail (T. lepturus)– which became a new resource, and posteriorly, revealed declining trends. Finally, to track changes in the trophic structure, we used landing data to calculate the mean trophic level index of landed species. We verified MTL trends in four groups: all landed species, species with TL > 4, TL ≥ 3.5 and TL < 3.5. We identified significant decline trends in MTL of species with TL > 4 and TL ≥ 3.5, while the MTL of all landed species and TL < 3.5 showed non-significant increase trends. Although the MTL of all species had no decline, we noticed that when the sardine and largehead hairtail are removed, there is a decline over time. The sardine is the main species landed in the period, followed by P. saltatrix and T. lepturus. The approach of excluding the sardine (low TL) and T. lepturus (high TL) from the is based on previous studies (Alleway et al. 2014). Studies have shown that fishing down can be masked due to declines in small pelagic species with low TL and marked by declines of large predators in the past (Blanchard et al. 2010, Coll et al. 2010). We suggest that this masking effect is a result from the overexploitation of sardine and T. lepturus.
Also, we checked the landing trends for species reported by fishers as overexploited and new targets. This approach helped us to identify significant declines in landings of species with high trophic levels (TL = 4 and TL ≥ 3.5) (figure 5a, b). In addition, the declining trends for high trophic level species were also observed through landings data (Kg) (figure 5c), and separately for the main species landed (figure 5d).

Materials and methods
L129-130. It is not the artisanal fisheries the one making trips. It is the fishermen those making the trips. Correct wording there.
R: Corrected accordingly (Lines 133 - 135).

L131. Clarify the meaning of the word secular in this context.
R: Secular means that this fishing activity has been developed for over a century in the region. Studies show that beach seine fishing is practiced with old fishing canoes, which were built by the first generations of fishers in Arraial do Cabo, since 1880. This information is a book entitled "Arraial 500" and was clarified in the text (Line 135).

L151. Snowball sampling has been suggested to sometime bias results of interviews. See:
Kirchherr J, Charles K (2018) Enhancing the sample diversity of snowball samples: Recommendations from a research projecton anti-dam movement in Southeast Asia. Plos ONE 13(8): e0201710.https://doi.org/10.1371/journal.pone.0201710

Powered by Editorial Manager® and ProduXion Manager® from Aries Systems Corporation

Explain how do you overcome these limitations or acknowledge the limitations of your strategy.
R: In fact, we used snowball sampling to complete our sample number. At the fishing pier, we approached fishers at random, and they were invited to participate in our research. In addition, we had the help of an experienced fisher known to the community. With this contact, it was possible to access a large number of interviewees and it mainly facilitated the process of interviews. Kirchherr & Charles (2018) recommend strategies to overcome snowball sampling limitations, as having previous personal contacts before starting snowball sampling. We therefore did not rely exclusively on snowball sampling.

L155. I would not call a beginner a fisher that has been fishing 15 years. Maybe change the name of this first category.
R: Thank you for the suggestion. We modified that term into "less experienced".

L156-157. What is the reasoning to differentiate fishers with 36-40yr of experience from those with >40 yr.? I am not arguing for a different categorization, but the reasoning behind this choice needs to be clear.
R: We chose these experience categories to accommodate similar sample sizes. Thus, there is a better distribution of the number of interviewees in each experience category. In addition, experience categories were based on previous studies (e.g. Maia et al. 2018). We have included an explanation in lines 164 – 173.

L173. Landings and catch are two different terms. Be clear in what data you actually obtained. See comment from above.
R: We have replaced "catches" by "landings".

L186-188. Revise this sentence. It is not clear what you are trying to say.
R: We have rephrased this fragment, see lines 196 – 198.

L196. Is "citations" the right word here?
R: Yes, we consider that the correct form is "citations" because it is the frequency at which a species was mentioned by the fishers as overexploited and new target species.

L212. The normal term is "count data"
R: Done. Line 224.

Results
L232-239. By looking at the distribution of your data points in Fig2a and 2c it is difficult to find a good fit for any model there. Can you provide a measure of the goodness of fit of your model? Something like AIC or a pseudo-R? include a table with the results of your GLM in the ms or in the Supplement.
R: As suggested by the reviewer, we added a table with the results of GLM (Please, see table 1).

L300-302. Fisheries landings data does not necessarily reflect the stock status. Work of Trevor Branch and co-workers among others has repeatedly shown the weaknesses of using catch data to determine stock status. This needs appropriate acknowledgement in the manuscript.
R: We agree with your comment, the study by Branch and collaborators was included in lines 80 - 83, this point was also argued in lines 439 – 448, in which we discussed that MTL index may not always reveal the true status of stocks. However, our work uses as a prior knowledge the information from local fishers, who reported that overexploited species such as Pomatomus saltatrix and Trichiurus lepturus, among many others, species whose declines have also been evidenced through landing data. In general, when fishers were asked about the status of fishing stocks, 92% of fishers reported a decline in stocks of target species. In addition, fishers were asked about the percentage of declines, and the highest percentages are between 50% and 80% (see figure in the answer above). This finding, added to the declining trends in landings of high trophic level species suggest the overexploitation scenario of fisheries in Arraial do Cabo. Old photographs help fishers' remember the past composition and abundance of landed species, and visualize change in fisheries (Fig. 6). These photographs reveal catches with large predators such as groupers and sharks, which

have become rare events in the region. Photographs can be valuable historical information sources. In previous studies, old photographs were used to estimate decreases in the maximum individual fish size and the proportion of large groupers caught in South Florida, USA (McClenachan, 2009).

L323-324. This statement is problematic. To my understanding your interviews did not ask about temporal trends to be able to compare those directly with the landings data that you are presenting. Please clarify how you are making these comparisons.
R: Thank you for your comment. Indeed our interviews did not ask fishers on the past vs. present day catches of each species, but solely on which fish species they considered overexploited, and which were new target species, based on their accumulated experiences. Our questionnaire was organized to detect overexploited and new target species, which were then compared to temporal trends in local landings. We understand that landing trends are similar to the species identified as overexploited by fishers, where we can see declines in landings, and also for new target species, for which landings increase (as they become new targets) and are followed by a decrease.

Discussion
L352-353. By looking at your Fig2a and 2c, I would argue that there is no clear pattern to make such a statement.
R: This sentence refers to the increase in the number of species reported by fishers as years of practice increase. We identified this pattern in figure 2a, c and through the GLM results, which show significant increases in the number of species cited as overexploited ($P = 0.003$) as well as for the number of species identified as new target species ($P = 0.014$) across years of practice. We understand that the variance around the number of species mentioned by fishers across experience categories gives a sense of no clear pattern, yet our model has revealed there are slight increases across each category.

L396-398. Again, your Fig 5a, does not fully support your statement.
R: We agree that figure 5a does not fully support our argument for the occurrence of this phenomenon in Arraial do Cabo. For clarification, we have corrected this statement in the fragment (lines 429 - 432) and throughout the manuscript. We also included a case study that showed the change in target species from demersal finfish to shellfish, which identified the fishing down process through a long series of landing data (1920 - 2010) and MTL. In our study, we used a short series of landing data (1992 - 2008) in combination with fisher's knowledge. Despite the short series of landing data, we interviewed 32 very experienced fishers (with practice time between 40 - 60 years) who also reported declines in fish stocks. In addition, landing data show significant declines in species with TL > 4 (Fig. 5c, d). Finally, we are cautious in affirming the occurrence of fishing down in Arraial do Cabo, although there is the possibility that it may be masked by other issues.

L402-404. From what I got from your methods, you used the landings data to derive mean trophic level of the landings per year. Less clear to me is how you are incorporating LEK into that analysis. Almost all the most important new fishery species identified by the fishers are high trophic level (>4). And the most important, T. lepturus is 4.4.
R: We agree that most of the new target species reported by fishers are of a high trophic level, such as T. lepturus (TL = 4.4) and B. capriscus (TL = 4.1). The fisher's knowledge helped us to identify the declines in top predators such as Pomatomus saltatrix (TL = 4.5), Trichiurus lepturus (TL = 4.4), Epinephelus marginatus (TL = 4.4), Seriola fasciata (TL = 4.5), and Caranx hippos (TL = 3.6). Yet, fishers reported a scenario of overfishing in the region, which 92% of them noticed alarming declines in stocks of the main target species of fishing. Another way to incorporate the fisher's knowledge in our analysis was comparing the MTL of overexploited with MTL of new target species mentioned by fishers (see Supporting information, Fig. S1). We observed that the citations of overexploited species are more frequently within higher TL species than new target species (see Supporting information, Fig. S1). In addition, the MTL observed for overexploited species was higher than expected at random.

L409-416. This sounds as if you would like to prove that fishing down the food webs is occurring in your study site, but your data does not tell that is actually happening. You

Powered by Editorial Manager® and ProduXion Manager® from Aries Systems Corporation

provide alternative explanations from other areas but don't provide an explanation or analysis for your case.
R: Thank you for your comment. We have rewritten this paragraph (Lines 439 - 448).

L418-419. Fig 6 show very impressive pictures, but your LEK study did not ask questions about tunas or sharks and their historical catches.
R: The shark species of the Carcharhinus genus have been cited by fishers as overexploited and new target species (see tables 2 and 3), as it is the case for C. brevipinna (Figure 6). Our mention of shark and tuna catches merely intended to offer the reader an insight into catches' composition at different time periods, as previous studies have identified declines in large predators in the region (Bender et al. 2014).

L425-430. Why Scarus trispinosus did not appear in your LEK results? Fishers did not recognize this species as overexploited. Also this whole section does not discuss anything related to your study.
R: Thank you for your comment. S. trispinosus did not appear in our results because this species is mainly a target of spearfishers. For this fishing gear, we interviewed two local fishers, and one of them mentioned the species Sparisoma axillare as a new target species. Another point that explains why this species was not mentioned by fishers is that, unlike in our study, Bender et al. (2014) asked questions directed towards parrotfish and grouper species, using photographs. Indeed, the collapse of parrotfishes in the same region is pretty well covered in Bender et al. (2014).

L436-440. By looking at your supplement data, the number of fishing boats has been reduced to around half of those reported in 1995. Fishing days in contrast have more than doubled. The average number of fishing days per boat per year in 1995 was only 5 days. In 2019 this value is 25 days per year. The 25 days value is still very low for a year. What are the explanation that you have for this? Are the methodologies to calculate these numbers comparable among reports?
R: We agree with your comment and removed that sentence (lines 468 - 471). In figure and table S2, the increase in fishing days corresponds to the most recent reports from PMAP-BS, both accounting for the fishing days of all fishing gear operating in Arraial do Cabo. These recent reports also consider gear that does not use boats, e.g. hand line, manual collection and some types of fishing nets, as beach seine. Although these reports used different methodologies to estimate the number of fishing days, we can still consider the number of boats as the fishing effort to compare the causes reported by fishers.

L448. In the manuscript you do not present any information about those management initiatives and how you relate those to your actual work
R: The only management initiatives existent in the local marine extractive reserve is regarding the spawning period for some species and few species prohibition. Indeed, there are no initiatives for stock recovering based on data produced locally. Additionally, local managers are poorly skilled to absorb state and national management rules to be locally applied and enforced.

L450. What is the likelihood that fishing quotas are going to be enforced in such areas? Is this a realistic strategy?
R: Yes, it is a realistic strategy since fishing is practiced in a relatively small partially protected MPA. Also, this MPA has financial and logistic resources to implement a collaborative co-management framework involving stakeholders and being informed by the small-scale fishing monitoring conducted by the state fishery agency. We clarified in the text (lines 480 - 485).

L469-471. This argument is counter-intuitive. Your manuscript is about using LEK to show the exploitation status of fisheries resources. And your recommendation now is to educate those fishers because they do not know about the status of their resources.
R: We agree with your suggestion and have rewritten this sentence. (Lines 507 - 512).

Useful reference missing in the ms:
Silvano, R.A.M. and Begossi, A., 2010. What can be learned from fishers? An integrated survey of fishers' local ecological knowledge and bluefish (Pomatomus saltatrix) biology on the Brazilian coast. Hydrobiologia, 637(1), p.3.
R: We added this reference in the introduction section.

Powered by Editorial Manager® and ProduXion Manager® from Aries Systems Corporation

Reviewer #3: Fogliarini et al., is an interesting ms dealing with the fishing down the food web process inferred from local ecological knowledge and landing data. The ms is pleasant and the ideas are organized and written with high quality. I have included a series of comments throughout the ms that I would like to see addressed before publication.
One important concern is regarding the different time scale from LEK data and from landing data. Interviews were performed from 2018 to 2019 and fishing land data from 1992 to 2008. there is at least a decade temporal difference on this data. Authors are confident that this do not imply results bias on the present study? I would like to see the development of this controversial time different in the ms discussion.
Results are well-presented and consistent. However, figures are very low quality and must be redone. See comments on the text. Fig 1 (map) is extremely low quality and unsuitable for publication.
Discussion is organized but lack of details in some important information. Comments are in the attached PDF.
I would be keen to review the ms once again before publication.
R. We thank Reviewer #3 for his/her comments and suggestions. We have tried to include all of the proposed alterations in this new MS version. We understand there is a time lag from landing data and the time when interviews were conducted but we are also certain that this does not affect our MS main conclusions. If the current time difference already reveals concordant patterns between landings trends and species mentioned as overexploited (declining trends) and new target species (increasing), we believe that if a longer landings data-series was available, that would make the patterns even clearer. If fishers were interviewed back in 2008, they would have possibly given the same answers on the current new target species and overexploited ones (see Bender et al. 2014 for overexploited taxa). We tried to have access to a longer landing data-series but did not receive any answers from local authorities that are responsible for fisheries monitoring. Data exists but won't be made available. We must also point out that targeted species have remained the same for the last 20 years (CEL Ferreira).

Major comments reviewer #3:
L46. ecological conclusion?? It is not a methodological paper...
The study adds extremely relevant information on the overexploitation status of many different species…etc.
R: We agree and modified this sentence into: "Our work reveals a depletion scenario of fishing stocks and changes in composition of fish species caught in the Arraial do Cabo."

L55. Fishing is not only an impact. Several fishing communities live from those resources and cope with the human X ocean relationship. Important to point out that unregulated and predatory fishing is the problem addressed herein.
R: Thank you for your comment. We agreed and modified this sentence. See line 54.

L62. Briefly mention the consequences please.
R: Among the consequences of unsustainable removal of top predators are the release of prey. In other words, the loss of top predators can cause numerical increases in meso-consumers and alter community structure. We mention other consequences in the following paragraph. Please see line 65.

L79."....have been proposed so far and our study aims to add information on..."
Complete your sentence as suggested so it would be more useful.
R: This sentence was rewritten (Lines 79 - 84).

L126. Poor figure quality. Difficult to identify where is study area in Brazilian coast.
I would like to suggest a new map with better quality.
R: We adjust the quality of figures.

L132. Not mentioned in the Fig where is the MPA.
R: The figure 1 was substitute by an adapted map due copyright for publication, in this new version there is no mention of the MPA.

L165. This seems like a discussion. Any reference for this idea?

Powered by Editorial Manager® and ProduXion Manager® from Aries Systems Corporation

R: We added the reference Mendonça et al. (2013).

L175. Interviews were acquired from 2018 to 2019 and fishing land data from 1992 to 2008. there is at least a decade temporal difference on this data. Authors are confident that this do not imply results bias on the present study? I would like to see the development of this controversial time different in the ms discussion.
R: Please see answer above (L180). We have also included that in the discussion.

L176. In the reference list? How this data was acquired?
R: It is not in the list of references because these data were acquired directly from the Fundação de Pesca de Arraial do Cabo - FIPAC. These is the first fisheries monitoring reports in Arraial do Cabo, and are available on the FIPAC website (see https://www.fipac.rj.gov.br).

L180. Again. As previously mentioned, this is a quite old data. Fishing data from 1992 (almost 30 years old) represents the MTL current scenario? Probably trophic levels are even worst nowadays. This must be discussed.
R: We agree with reviewer's comment that there are temporal differences in the data sources. Fisheries statistics were obtained through Fundação Instituto da Pesca de Arraial do Cabo (FIPAC) which shared only a short monitoring time series (1992 – 2008). We agree that the current scenario for trophic levels can be even worse, as our results showed declines in high-trophic species.

L346. This result came out of nowhere. Please, describe in the aims, methods etc.
R: We describe this result in all sections of our manuscript. Please see how we described in each section:
Introduction (Objectives): "We also discuss the main causes of fish overexploitation and management strategies in a subtropical fisheries hotspot."
Materials and methods (Data collection): "(iii) what are the main causes of species' overexploitation".
Results: "Fishers mentioned 35 different possible causes of fisheries overexploitation in Arraial do Cabo (1.58 ± 1.08, mean ± standard deviation). The five most mentioned causes were the increased fishing effort (22%), which is recognized by fishers as an increase in the number of fishers and boats; presence of industrial fishing vessels (21%), presence of purse seine fisheries (19%), gillnet fishing (16%), and trawling (12%)."
In the following paragraph of the discussion: "The cause more mentioned by fishers as responsible for fisheries overexploitation was increase in the fishing effort…"

L366. where are the ecological implications? this paragraph needs to end up with a broad view of your main results.
R: We agree with your suggestion and incorporated more explanations in this paragraph. Please see lines 396-400.

L465. how small? Is a no-take zone implementation process been currently discussed with local community?
R: We correct the term "small MPAs" to "small no-take areas". The implementation process had been discussed with the local community recently. In 2021 a management plan with the contribution of researchers, community members, and fishers, will suggest the implementation of 3 to 4 small no-take areas within the extractive marine reserve. It is a participatory process, which will establish specific rules for the use of each of these areas.

L465. how short? More info is necessary.
R: We add more explanation in lines 497 - 500.

L480. I would like to see more discussion on the conservation strategies along the ms discussion.
R: We added more details on the management strategies, following the reviewers' suggestion. Now, we describe, minimum landing size of capture, closure periods how fishing quotas can be implemented in a participatory manner the importance of no-take MPAs, a improve of surveillance for banning the illegal fisheries and the importance of young generations acknowledging the past abundance of marine resources to be more willing to accept management and conservation initiatives (lines 478 - 504).

Powered by Editorial Manager® and ProduXion Manager® from Aries Systems Corporation

	References<br>Alleway HK, Connell SD, Ward TM, & Gillanders BM. (2014). Historical changes in mean trophic level of southern Australian fisheries. Marine and Freshwater Research, 65(10), 884-893.<br>Bender MG, Machado GR, de Azevedo Silva PJ, Floeter SR, Monteiro-Netto C, Luiz OJ, & Ferreira CE. (2014). Local ecological knowledge and scientific data reveal overexploitation by multigear artisanal fisheries in the Southwestern Atlantic. PLoS One, 9(10), e110332.<br>Blanchard JL, Coll M, Trenkel VM, Vergnon R, Yemane D, Jouffre D, et al. (2010). Trend analysis of indicators: a comparison of recent changes in the status of marine ecosystems around the world. ICES J Mar Sci. 67 (4): 732-744.<br>Branch et al. (2010). The trophic fingerprint of marine fisheries. Nature 468, 431–435.<br>Branch TA. (2015). Fishing Impacts on Food Webs: Multiple Working Hypotheses. 576 Fisheries. 40 (8): 373-375.<br>Coll M, Shannon LJ, Yemane D, Link JS, Ojaveer H, Neira S, et al. (2010). Ranking the ecological relative status of exploited marine ecosystems. ICES J Mar Sci. 67 (4): 769-786.<br>Da Silva, L. D. M. C., Machado, I. C., dos Santos Tutui, S. L., & Tomás, A. R. G. (2020). Local ecological knowledge (LEK) concerning snook fishers on estuarine waters: Insights into scientific knowledge and fisheries management. Ocean & Coastal Management, 186, 105088.<br>FAO. 2020. The State of World Fisheries and Aquaculture 2020. Sustainability in action. Rome. https://doi.org/10.4060/ca9229en<br>Ferreira, H. M., Reuss-Strenzel, G. M., Alves, J. A., & Schiavetti, A. (2014). Local ecological knowledge of the artisanal fishers on Epinephelus itajara (Lichtenstein, 1822) (Teleostei: Epinephelidae) on Ilhéus coast–Bahia State, Brazil. Journal of ethnobiology and ethnomedicine, 10(1), 51.<br>Herbst, D. F., & Hanazaki, N. (2014). Local ecological knowledge of fishers about the life cycle and temporal patterns in the migration of mullet (Mugil liza) in Southern Brazil. Neotropical Ichthyology, 12(4), 879-890.<br>Kirchherr, J., Charles, K. (2018). Enhancing the sample diversity of snowball samples: Recommendations from a research projecton anti-dam movement in Southeast Asia. Plos ONE 13(8): e02017<br>McClenachan, L. (2009). Historical declines of goliath grouper populations in South Florida, USA. Endangered Species Research, 7(3), 175-181.<br>Sethi et al. (2010). Global fishery development patterns are driven by profit but not trophic level. PNAS 107, 12163-12167.<br>Silvano, R. A. M., & Begossi, A. (2010). What can be learned from fishers? An integrated survey of fishers' local ecological knowledge and bluefish (Pomatomus saltatrix) biology on the Brazilian coast. Hydrobiologia, 637(1), 3.
**Additional Information:**	
**Question**	**Response**
**Financial Disclosure**<br><br>Enter a financial disclosure statement that describes the sources of funding for the work included in this submission. Review the submission guidelines for detailed requirements. View published research articles from *PLOS ONE* for specific examples.<br><br>This statement is required for submission and **will appear in the published article** if the submission is accepted. Please make sure it is accurate.	C.O.F. acknowledges Coordenação de Aperfeiçoamento de Pessoal de Nível Superior (CAPES) for financial support. V.J.G. received a post-doctoral grant #2017/22273-0, São Paulo Research Foundation (FAPESP). CELF is supported by grants from FAPERJ and CNPq. Costão Rochoso Project (FUNBIO Grant Pesquisa Marinha 020/2017) has provided financial, and logistic support during field sampling in Arraial do Cabo. This work is part of a research program supported by Serrapilheira Institute (grant number Serra-1708-15364) awarded to Guilherme Ortigara Longo (PI).

**Unfunded studies**<br>Enter: *The author(s) received no specific funding for this work.*<br><br>**Funded studies**<br>Enter a statement with the following details:<br>• Initials of the authors who received each award<br>• Grant numbers awarded to each author<br>• The full name of each funder<br>• URL of each funder website<br>• Did the sponsors or funders play any role in the study design, data collection and analysis, decision to publish, or preparation of the manuscript?<br>• **NO** - Include this sentence at the end of your statement: *The funders had no role in study design, data collection and analysis, decision to publish, or preparation of the manuscript.*<br>• **YES** - Specify the role(s) played.<br><br>* typeset	
**Competing Interests**<br><br>Use the instructions below to enter a competing interest statement for this submission. On behalf of all authors, disclose any competing interests that could be perceived to bias this work—acknowledging all financial support and any other relevant financial or non-financial competing interests.<br><br>This statement **will appear in the published article** if the submission is accepted. Please make sure it is accurate. View published research articles from *PLOS ONE* for specific examples.	NO authors have competing interests

**NO authors have competing interests**<br><br>Enter: *The authors have declared that no competing interests exist.*<br><br>**Authors with competing interests**<br><br>Enter competing interest details beginning with this statement:<br><br>*I have read the journal's policy and the authors of this manuscript have the following competing interests: [insert competing interests here]*<br><br>* typeset	
**Ethics Statement**<br><br>Enter an ethics statement for this submission. This statement is required if the study involved:<br><br>• Human participants<br>• Human specimens or tissue<br>• Vertebrate animals or cephalopods<br>• Vertebrate embryos or tissues<br>• Field research<br><br>Write "N/A" if the submission does not require an ethics statement.<br><br>General guidance is provided below. Consult the submission guidelines for detailed instructions. **Make sure that all information entered here is included in the Methods section of the manuscript.**	The interviews were approved by the Ethics Committee of Universidade Federal de Santa Maria, Brazil (CAAAE 29157919.6.0000.5346). The form of consent obtained was written.

**Format for specific study types**<br><br>**Human Subject Research (involving human participants and/or tissue)**<br>• Give the name of the institutional review board or ethics committee that approved the study<br>• Include the approval number and/or a statement indicating approval of this research<br>• Indicate the form of consent obtained (written/oral) or the reason that consent was not obtained (e.g. the data were analyzed anonymously)<br><br>**Animal Research (involving vertebrate animals, embryos or tissues)**<br>• Provide the name of the Institutional Animal Care and Use Committee (IACUC) or other relevant ethics board that reviewed the study protocol, and indicate whether they approved this research or granted a formal waiver of ethical approval<br>• Include an approval number if one was obtained<br>• If the study involved *non-human primates*, add *additional details* about animal welfare and steps taken to ameliorate suffering<br>• If anesthesia, euthanasia, or any kind of animal sacrifice is part of the study, include briefly which substances and/or methods were applied<br><br>**Field Research**<br><br>Include the following details if this study involves the collection of plant, animal, or other materials from a natural setting:<br>• Field permit number<br>• Name of the institution or relevant body that granted permission	
**Data Availability**<br><br>Authors are required to make all data underlying the findings described fully available, without restriction, and from the time of publication. PLOS allows rare exceptions to address legal and ethical concerns. See the PLOS Data Policy and FAQ for detailed information.	Yes - all data are fully available without restriction

A Data Availability Statement describing where the data can be found is required at submission. Your answers to this question constitute the Data Availability Statement and **will be published in the article**, if accepted.<br><br>**Important:** Stating 'data available on request from the author' is not sufficient. If your data are only available upon request, select 'No' for the first question and explain your exceptional situation in the text box.<br><br>Do the authors confirm that all data underlying the findings described in their manuscript are fully available without restriction?	
**Describe where the data may be found in full sentences. If you are copying our sample text, replace any instances of XXX with the appropriate details.**<br><br>• If the data are **held or will be held in a public repository**, include URLs, accession numbers or DOIs. If this information will only be available after acceptance, indicate this by ticking the box below. For example: *All XXX files are available from the XXX database (accession number(s) XXX, XXX.*).<br>• If the data are all contained **within the manuscript and/or Supporting Information files**, enter the following: *All relevant data are within the manuscript and its Supporting Information files.*<br>• If neither of these applies but you are able to provide **details of access elsewhere**, with or without limitations, please do so. For example:<br><br>*Data cannot be shared publicly because of [XXX]. Data are available from the XXX Institutional Data Access / Ethics Committee (contact via XXX) for researchers who meet the criteria for access to confidential data.*<br><br>*The data underlying the results presented in the study are available from (include the name of the third party*	This information will only be available after acceptance.

and contact information or URL).<br>• This text is appropriate if the data are owned by a third party and authors do not have permission to share the data.<br><br>* typeset	
Additional data availability information:	Tick here if the URLs/accession numbers/DOIs will be available only after acceptance of the manuscript for publication so that we can ensure their inclusion before publication.

Powered by Editorial Manager® and ProduXion Manager® from Aries Systems Corporation

Manuscript Click here to access/download;Manuscript;Manuscript.docx

Telling the same story: fishers and landing data reveal changes in fisheries on the Southeastern Brazilian Coast

Carine O. Fogliarini[a*], Carlos E.L. Ferreira[b], Jéssica Bornholdt[a], Moysés. C. Barbosa[b], Vinicius J. Giglio[c], Mariana G. Bender[a]

[a]Marine Macroecology and Conservation Lab, Departamento de Ecologia e Evolução, Universidade Federal de Santa Maria. Avenida Roraima, 1000, prédio 17 sala 1140, Bairro Camobi, Santa Maria, Rio Grande do Sul, Brazil.

[b]Reef Systems Ecology and Conservation Lab, Departamento de Biologia Marinha, Universidade Federal Fluminense, Niterói, RJ, Brazil.

[c]Marine Conservation and Ecology Lab, Instituto do Mar, Universidade Federal de São Paulo, Santos, São Paulo, Brazil.

* Corresponding author
E-mail: carine_fogliarini@hotmail.com

Abstract

An understanding of the effects of fishing on marine ecosystems relies on information about the conserved state of these environments. Non-conventional approaches such as the use of historical data and local ecological knowledge can provide information and help adjust our references of changes in the environment. Also, the combination of different types of data can indicate a fisheries trend that would be undetectable when evaluated separately. Here we investigated changes in fisher's perceptions regarding overexploited and new target species in artisanal fisheries in a secular fishing village of the subtropical, southeastern Brazilian coast. We identified temporal changes in landings and in the mean trophic level (MTL) of high trophic level species (≥ 3.5 and >4) over 16 years. Fishers' knowledge revealed shifts in perception associated with years of fishing practice. More experienced fishers recognized a greater number of overexploited and new target species than fishers in the beginning of their careers. Landing data has revealed declining trends of 72% for five mesopredators species. Due to the overfishing of mesopredators, there was a shift in target species, towards fish that were previously discarded. Temporal changes in landings and in the MTL metric are concordant with previous reports on the overexploitation of species caught by local fishers. Our work reveals that multiple sources of information can be combined to establish historical baselines and improve the detection of change in marine ecosystems.

Keywords: Shifting baseline, marine resources, overfishing, local ecological knowledge, fisheries monitoring, fisheries management

Introduction

Marine and coastal ecosystems are globally threatened due to human impacts such as overfishing, pollution, habitat destruction and climate change [1,2]. Indeed, anthropogenic activities have been changing the oceans for millennia, with fishing being the most ancient activity performed by humans [3]. Some types of fishing have been unsustainable [4], surpassing the recovery limits of several fish stocks across the globe [5]. The status assessment of fishery resources revealed that the fraction of fish stocks that are within biologically sustainable levels decreased from 90%, in 1974, to 65.8% in 2017 [6]. In contrast, recent studies argue that some fishing stocks are gradually recovering in different parts of the world [7], especially in regions where there are effective fisheries management [8]. Although these recent findings show positive global scenarios [9], overfishing is responsible for substantially modifying the abundance and biomass patterns of species and decreasing the body size of individuals in a population [10–13]. Some fishing gears such as longline are usually directed towards top predators (e.g. tunas and sharks) and select individuals with larger body sizes [14], a common pattern with known consequences for marine ecosystem functioning, including the prey release effect [15,16].

Fishing effort concentrated on large-bodied fish can cause the decline of top predators stocks such as groupers, sharks and tunas [17–21], and even drive species to local extinction [3,22]. These large-bodied species tend to occupy higher levels in the food chain and play a key role in maintaining the trophic structure and functioning of communities [23,24]. Thus, the removal of top predators can trigger cascading effects on lower levels of the food web [24–26]. Declining trends of higher trophic level stocks lead to a systematic replacement by lower trophic level and smaller-bodied target species, a process identified as "fishing down the food web" [27–29]. This process has been measured through a decline in fisheries' mean trophic level (MTL) [30–33]. In an attempt to understand how fishing impacts marine food

webs, studies highlight that there are other ways in which fishing may be affecting the ecosystem. In “fishing through the food web” a decline in MTL is caused due to the sequential addition of low trophic level rather than to a decline of high trophic level catches [25]. The “fishing up the food web” process corresponds to a shift from low to high trophic level species over time, causing an increase in the MTL of catches [34,35]. Multiple hypotheses may explain how fishing affects marine food webs [36], such as profit-driven fishing [37]. The use of catch MTL as an indicator to measure the magnitude of fishing impacts on marine ecosystems may not be reliable in some scenarios, and should be interpreted cautiously [38]. The use MTL in combination with other measures may offer a better picture of the ecosystem [39].

The assessment of temporal changes in MTL and replacement of target species requires long-term fisheries monitoring. However, most low and lower-middle income countries lack fisheries monitoring programs [40]. In Brazil, such programs are rare and fragmented, and since 2008, there is no governmental fisheries monitoring program established at the national level. In addition, lack of financial resources, a large (~8000 km) and heterogeneous coast, a multigear fishing scenario, differential efforts and target species, pose a challenge to the implementation of fisheries management strategies in Brazil [41,42]. To circumvent the difficulties caused by the lack of fishing data, non-conventional approaches can be useful to estimate the impacts of fishing over time [43–45]. For instance, local ecological knowledge (LEK) may provide a relevant source of historical information, in which marine resource users (e.g. fishers) experienced different scenarios in the past [44,46,47].

A robust assessment of the magnitude and effect of anthropogenic impacts (e.g. fishing) on ecosystems requires knowledge of the unexplored state of these environments [48]. However, throughout human generations, information on composition, size and

abundance of species caught on fisheries may be lost, compromising the environmental perception adopted by human populations [49]. Such cross-generational change in baselines has implications to environmental issues, as it results in increased tolerance for progressive environmental degradation because the younger generations consider a more degraded environment as the norm [50,51]. Pauly et al. [50] described this phenomenon as the “shifting baseline syndrome”, a socio-psychological tendency to assume that "natural" conditions of an ecosystem are those first observed by users, researchers and contemporary managers. When data on the conservation status of the environment is scarce, historical data from alternative sources (e.g. works of art, reports of naturalists, logbooks, photographs, grey literature, and anecdotal information) have been used to help adjust references [52].

Fisher’s LEK has been widely used to investigate the past of fisheries, mainly in data poor countries. The empirical knowledge of natural resource users is a cost-effective alternative approach that offers relevant data to inform management. For instance, fishers LEK has provided information on temporal and spatial patterns of catches [53–55], behavior of targeted species [42,56–59], changes in abundance and body size of targeted species [20,60], trophic and ecosystem models [61,62], and conflicts with other resource users and management interventions [63,64]. In certain cases, LEK has been also associated with other types of data, such as landing data and underwater visual census, to reveal temporal trends in fish populations [54,61,65–67]. These combined approaches offer an opportunity to understand the effect of small-scale fisheries in data poor scenarios – when temporal and systematically collected data are missing. Combining anecdotal evidence from fisher’s LEK and fisheries landing data, we aim to identify temporal changes in (i) small-scale fisher’s perception regarding overexploited and new target fish species; (ii) and landings of overexploited and new target fish species. Specifically, we combined the MTL metric with fishers' perceptions to reveal temporal changes in local trophic structure caused by fishing.

We also discuss the main causes of fish overexploitation and management strategies in a subtropical fisheries hotspot.

Materials and methods

Study site

The study was conducted in Arraial do Cabo, southeastern Brazil (Fig 1). Arraial do Cabo is an ancient Brazilian fishing area, where a variety of artisanal fishing gear is employed such as hook and line, gillnet, spearfishing and beach seine. In Arraial do Cabo, artisanal fisheries still occur with small boats such as canoes some without any motors. Fishers make trips near the shore, or using hook and line or small nets like cast nets in the rocky shore or on the beach [68]. The beach seine is an old/secular practice that was central to the establishment of the local partially protected marine protected area (MPA) – the Arraial do Cabo Marine Extractive Reserve. In this MPA, only local fishers are allowed to fish, with 1600 families relying on fishing activities as the main source of income [68]. Arraial do Cabo holds a diverse marine community, gathering tropical and warm temperate components, which is favored by the coastal morphology and local upwelling events [69,70].

Fig 1. Map of Arraial do Cabo region (state of Rio de Janeiro, Brazil), showing interview sites. Map reprinted from [54] under a CC BY license, with permission from Bender et al (2014), original copyright 2014.

Data collection

Fisher’s local ecological knowledge

Between July and August 2018 and 2019, we interviewed 155 artisanal fishers on the fishing communities of Figueira, Monte Alto, Praia Grande, Praia dos Anjos, Prainha, and Pontal (Fig 1). Interviewed fishers correspond to 10.3% of local artisanal fishers (n = 1500) [71]. Interviews were conducted following previous consent and individually, so that there was no influence from others on fishers' answers. Semi-structured questionnaires included questions on (i) which fish species were previously discarded but are now fishing targets; (ii) which species are overexploited in the region; and (iii) what are the main causes of species' overexploitation (S1 File). The fishers answered spontaneously which species they consider overexploited and new targets of fisheries. Fishers were randomly approached at Praia dos Anjos pier, and invited to participate in the interviews. This procedure was conducted until reaching an appropriate number of interviews (n). When completing the questionnaire (S1 File), fishers were asked to indicate another interviewee [72].

We also asked fishers their age and years of fishing experience. For data analysis, we chose to use the fisher's experience (time period) rather than age, as these two variables are correlated ($r^2 = 0.53$, cor = 0.74, $P < 0.001$, Pearson's correlation test). Fishers were categorized according to their fishing experience into four groups: less experienced (<20 years, n = 24), intermediate (21-35 years, n = 55), experienced (36-40 years, n = 44) and very experienced (>40 years, n = 32). Under this classification, we consider only their time of experience (years of practice), with no qualitative judgment of the real experience of each fisher [73]. We assume that within a given category of experience, all fishers spend the same time fishing, and all gain experience at the same rate [73]. Still, this set of different categories can help estimate changes in fish composition as well as differences in perceptions of trends across generations of fishers. In addition, we chose these experience categories so that they would accommodate similar sample sizes. The small sample size of the less experienced category compared to the very experienced is related to the lack of engagement with fishing

by the younger generations. Younger fishers are switching to alternative sources of income such as tourism, often encouraged by their families to leave the fishing [71]. The interviews were approved by the Ethics Committee of Universidade Federal de Santa Maria, Brazil (CAAAE 29157919.6.0000.5346) and by Sistema de Autorização e Informação em Biodiversidade (Sisbio-ICMBio/IBAMA/Brazil nr. 55911-6).

Fisheries landing data

We searched for landing data on the main species reported by the interviewees. Landing data (e.g. in kg) has been incorporated into the study to complement the trends identified by local fishers. Such data corresponds to local annual landings of artisanal fisheries registered between 1992 and 2008 in the port of Arraial do Cabo (Fisheries landing monitoring report, FIPAC).

Mean trophic level

We compiled the trophic level (TL) of 81 species landed in Arraial do Cabo between 1992 and 2008 (S1 Table). The trophic levels were obtained from FishBase, which provides TL estimates from food items for many fish species [74]. For the TL of invertebrates, we used the equivalent database, SeaLifeBase [75].

To assess MTL trends landings over time, we compared the MTL of species in five groups: all landed species, species with TL > 4, TL ≥ 3.5, and TL < 3.5. These categories allow us to assess changes in MTL taking into account the temporal dynamics of higher and lower trophic level species, and visualize potential masking effects [31]. MTL was also calculated excluding low TL species (TL < 3.5), because of possible fisheries expansion in direction to small pelagic species, which can represent an alternative scenario such as ‘fishing through’ food webs rather than ‘fishing down’ [25]. High trophic level species (TL > 4) are

more sensitive to overfishing, as they have life history traits such as late maturation, low fertility rates, and slow growth [76]. Therefore, we examined the dynamics of species' stocks with a threshold value of TL > 4, as done previously [25]. Species with TL > 3.5 (e.g. tunas, cod and groupers) are considered as mesopredators that feed on a mixture of low and high-TL organisms [77]. Also, species with TL < 3.5 were assessed to observe changes in the contribution of low trophic level species [77]. We used these threshold values to eliminate the masking effect and to emphasize changes in the relative abundance of fish from certain trophic groups. The MTL was estimated as follows [27]:

$$MTL = \frac{\sum TL_{ij} Y_{ij}}{\sum Y_{ij}}$$

Where MTL is the mean trophic level of landing in year *j*, *Yij* the landing of species *i* in year *j* and *TLi* is the trophic level of species *i*.

We used a null model approach to test whether citations of overexploited and new target species (by fishers), within a given trophic level, are more frequent than expected at random. We generated 1000 random samples for the number of citations of each species within the overexploited and new target species groups. For each random sample, we calculated the MTL weighted by the citation number, using the following equation:

$$MTLc = \frac{\sum TLi\ Ci}{\sum Ci}$$

Where MTL_c is the mean trophic level of overexploited and new target species and *Ci* is the total number of citations of species *i*.

We then plotted the confidence intervals (C.I. 95%) of the null MTL obtained for overexploited and new target species and the observed MTL_c, to verify if the citation frequency differs than expected at random.

Data analysis

To estimate changes in fishers' perception of target species, we applied a Generalized Linear Model (GLM) between the (i) number of overexploited species and (ii) number of new target species vs. experience of fishers (years of practice). The number of cited species was set as the response variable. Because numbers of species are count data, we assumed a Poisson distribution. The experience represents the explanatory variable. Under these specifications, the equation of model was:

$$log(\mu i) = \beta 0 + \beta 1\ xi$$

Where μi is the conditional expectation of yi, E(y|x), $\beta 0$ is the coefficient marked (Intercept) and $\beta 1$ the coefficient marked x. We assumed the model of the mean number of species μi using a log link.

We measured the mean percent of increase in citations (P) of overexploited fish species and new target species according to fishers' experience (F) using the GLM model coefficients (*coef1* and *coef2*):

$$P = \left(\frac{e^{(coef1 + coef2 * F)}}{e^{(coef1 + coef2 * 0)}} - 1 \right) * 100$$

To test for differences between the proportion of overexploited and new target species (previously discarded) mentioned by fishers in different experience categories, we applied a Z-test. We then verified differences between groups through a pairwise comparison of proportions. Regression analyses were used to measure temporal trends in landings of fish species by plotting the landed weight (in tons) over the years. All analyzes were performed on R software v. 3.6.1 [78].

Results

Fishers with more years of practice recognized a significantly greater number of overexploited fish species than those with fewer years of practice (β = 0.009, df = 153, P = 0.008) (Fig 2a, S2 Table). This relationship is clearly detected by an increase in the mean percent in the citation, related with years of practice (Fig 2b). We observed the same pattern for the number of species recognized as new target species. As time of practice increases, fishers mentioned a significantly greater number of species as new targets of local fisheries (β = 0.010, df = 153, P = 0.01) (Fig 2c, S2 Table). In addition, there is an increase in the percent of citations of new target species by fishers' with more years of practice (Fig 2d).

Fig 2. (a) Number of species mentioned as overexploited according to fisher's experience (years of practice); (b) Probability of citations of overexploited species according to fisher's experience; (c) Number of species mentioned as new target species according to fisher's experience; and (d) Probability of citations of new target species according to fisher's experience. Experience categories: LESS_EXP = less experienced; INTER = intermediate; EXP = experienced; and VER_EXP = very experienced.

In total, 37 species were identified as overexploited by local fishers (Table 1). The bluefish (*Pomatomus saltatrix*) was the most frequently cited species, across all experience categories. The proportion of citations of *P. saltatrix* were similar between categories, with at least 45% of fishers in different experience categories recognizing bluefish as overexploited: 56% of very experienced (n = 18), 55% of experienced (n = 21), 47% of intermediate (n = 18), and 45% of less experienced fishers (n = 21) (Fig 3a). The second most cited species was the largehead hairtail (*Trichiurus lepturus*), although there are no significant differences between the experience categories, the species was mentioned more by intermediates (26%, n = 10) and less experienced (30%, n = 14) fishers. The dusky grouper (*Epinephelus*

marginatus) and the crevalle jack (*Caranx hippos*) were most frequently cited by very experienced fishers (> 40 years of practice) and had a significant difference in the proportion of citations between very experienced and beginners (P = 0.03 and P = 0.001, respectively). In addition to *C. hippos*, the lesser amberjack (*Seriola fasciata*) was another carangid referred to as overexploited (Fig 3a).

Table 1. Species reported as overexploited by fishers in descending order of number of citations (N). Percentage of citation (%) of species by fishers (n = 155). Trophic levels were obtained from FishBase where a higher value means higher trophic levels (Froese and Pauly, 2019). Trophic categories were obtained from the Southwestern Atlantic reef fish database (Pinheiro et al., 2018). The conservation status at global level derives from the IUCN Red List of Threatened Species (IUCN, 2019) and at national level from the Brazilian Red List of Threatened Species (MMA, 2018).

OVEREXPLOITED SPECIES							
**Order**	**Species**	**N**	**%**	**Trophic level**	**Trophic category**	**Global status**	**Regional status**
1	*Pomatomus saltatrix*	78	50.3	4.5	MCAR	VU	NT
2	*Trichiurus lepturus*	37	23.8	4.4	MCAR	LC	LC
3	*Epinephelus marginatus*	23	14.8	4.4	MCAR	VU	VU
4	*Seriola fasciata*	22	14.1	4.5	MCAR	LC	DD
5	*Caranx hippos*	20	12.9	3.6	MCAR	LC	LC
6	*Pseudopercis numida*	20	12.9	3.9	MCAR	LC	NT
7	*Hyporthodus niveatus*	16	10.3	4	MCAR	VU	VU
8	*Seriola lalandi*	14	9	4.2	MCAR	LC	LC

9	*Mugil liza*	11	7	2	HERB	DD	NT
10	*Scomberomorus cavalla*	11	7	4.4	MCAR	LC	LC
11	*Sardinella brasiliensis*	10	6.4	3.1	PLANK	DD	DD
12	*Conger orbignyanus*	10	6.4	3.7	MCAR	LC	DD
13	*Sarda sarda*	8	5	4.5	MCAR	LC	LC
14	*Cynoscion striatus*	7	5	3.9	MCAR	NE	NE
15	*Mycteroperca microlepis*	6	3.8	3.7	MCAR	VU	DD
16	*Coryphaena hippurus*	6	3.8	4.4	MCAR	LC	LC
17	*Seriola dumerili*	6	3.8	4.5	MCAR	LC	LC
18	*Priacanthus arenatus*	5	3.2	4	MINV	LC	LC
19	*Katsuwonus pelamis*	5	3.2	4.4	MCAR	LC	LC
20	*Caranx crysos*	4	2.5	4.1	MCAR	LC	LC
21	*Caranx latus*	4	2.5	4.2	MCAR	LC	LC
22	*Pagrus pagrus*	3	1.9	3.9	MINV	LC	DD
23	*Trachinotus carolinus*	3	1.9	3.5	MINV	LC	LC
24	*Micropongonias furnieri*	3	1.9	3.1	MINV	LC	LC
25	*Cynoscion acoupa*	3	1.9	4.1	MCAR	LC	NT
26	*Lopholatilus villarii*	2	1.2	3.8	MCAR	NE	VU
27	*Balistes vetula*	2	1.2	3.8	MINV	NT	NT
28	*Diplodus argenteus*	2	1.2	3.1	OMNI	LC	LC

29	*Peprilus paru*	2	1.2	4.5	MCAR	LC	LC
30	*Centropomus undecimalis*	2	1.2	4.2	MCAR	LC	LC
31	*Scomber colias*	1	0.6	3.4	MCAR	LC	LC
32	*Scomberomorus brasiliensis*	1	0.6	3.3	MCAR	LC	LC
33	*Aluterus monoceros*	1	0.6	3.8	OMNI	LC	NT
34	*Lagocephalus laevigatus*	1	0.6	4	MCAR	LC	LC
35	*Carcharhinus leucas*	1	0.6	4.3	MCAR	NT	NT
36	*Isurus oxyrinchus*	1	0.6	4.5	MCAR	NT	NT
37	*Pseudobatos horkelii*	1	0.6	3.8	MINV	CR	CR

Trophic categories: Macrocarnivore (MCAR); Mobile Invertivore (MINV); Omnivore (OMNI); Planktivore (PLANK); Herbivore (HERB). IUCN categories: Critically endangered (CR); Vulnerable (VU); Near threatened (NT); Least Concern (LC); Data deficient (DD); Not evaluated (NE).

Fig 3. Fish species most frequently cited as overexploited (a) and new target species (b) according to fisher's experience (years of practice). Letters indicate significance ($P < 0.05$) between categories of experience of each species.

Fishers identified 36 new target species that were previously discarded by local fisheries (Table 2). *T. lepturus* was cited by 63% of very experienced fishers, 76% of experienced, 71% of intermediates, and 66% of less experienced fishers (Fig 3b). The second most cited species was the grey triggerfish (*Balistes capriscus*), followed by the Argentine conger (*Conger orbignyanus*), the unicorn leatherjacket filefish (*Aluterus monoceros*) and the Atlantic bigeye (*Priacanthus arenatus*) (Fig 3b). For *T. lepturus* and *C. orbignyanus* the proportion of citations was similar across experience categories (Fig 3b). The citations for *P.*

arenatus decreased with fisher's experience, therefore being greater for less experienced fishers. Only the citations of *B. capriscus* had significant differences between experience categories, being proportionally higher for experienced compared to intermediate fishers (P = 0.01) (Fig 3b).

Table 2. Species cited as new targets by fishers, in descending order of reports (N). Percentage of citation (%) of species by fishers (n = 155). Trophic levels were obtained from FishBase where a higher value means higher trophic levels (Froese and Pauly, 2019). Trophic categories were obtained from the Southwestern Atlantic reef fish database (Pinheiro et al., 2018). The conservation status at global level derives from the IUCN Red List of Threatened Species (IUCN, 2019) and at national status from the Brazilian Red List of Threatened Species (MMA, 2018).

NEW TARGET SPECIES							
**Order**	**Species**	**N**	**%**	**Trophic level**	**Trophic category**	**Global status**	**Regional status**
1	*Trichiurus lepturus*	107	69	4.4	MCAR	LC	LC
2	*Balistes capriscus*	27	17.4	4.1	MINV	VU	NT
3	*Conger orbignyanus*	26	16.7	3.7	MCAR	LC	DD
4	*Aluterus monoceros*	24	15.4	3.8	OMNI	LC	NT
5	*Priacanthus arenatus*	23	14.8	4	MINV	LC	LC
6	*Scomber colias*	13	8.3	3.4	MCAR	LC	LC
7	*Sardinella brasiliensis*	12	7.7	3.1	PLANK	DD	DD
8	*Katsuwonus pelamis*	11	7	4.4	MCAR	LC	LC
9	*Lagocephalus laevigatus*	9	5.8	4	MCAR	LC	LC
10	*Diplodus argenteus*	8	5.1	3.1	OMNI	LC	LC
11	*Porichthys porosissimus*	5	3.2	3.7	MINV	NE	LC
12	*Mugil liza*	4	2.5	2	HERB	DD	NT
13	*Pomatomus saltatrix*	4	2.5	4.5	MCAR	VU	NT
14	*Micropogonias furnieri*	4	2.5	3.1	MINV	LC	LC

15	*Cynoscion striatus*	3	1.9	3.9	MCAR	NE	NE
16	*Umbrina canosai*	3	1.9	3.9	MINV	NE	LC
17	*Selene setapinnis*	3	1.9	3.7	MCAR	LC	LC
18	*Dactylopterus volitans*	3	1.9	3.7	MINV	LC	LC
19	*Trachinotus carolinus*	2	1.2	3.5	MINV	LC	LC
20	*Caranx latus*	2	1.2	4.2	MCAR	LC	LC
21	*Cynoscion acoupa*	2	1.2	4.1	MCAR	LC	NT
22	*Chloroscombrus chrysurus*	2	1.2	3.5	PLANK	LC	LC
23	*Seriola fasciata*	2	1.2	4.5	MCAR	LC	DD
24	*Pseudopercis numida*	2	1.2	3.9	MCAR	LC	NT
25	*Carcharhinus plumbeus*	1	0.6	4.5	MCAR	VU	CR
26	*Isurus oxyrinchus*	1	0.6	4.5	MCAR	NT	NT
27	*Squatina argentina*	1	0.6	4.1	MCAR	EN	CR
28	*Sparisoma axillare*	1	0.6	2	HERB	DD	VU
29	*Epinephelus marginatus*	1	0.6	4.4	MCAR	VU	VU
30	*Carcharhinus brevipinna*	1	0.6	4.2	MCAR	VU	DD
31	*Cynoscion jamaicensis*	1	0.6	3.8	MCAR	LC	LC
32	*Pseudobatos horkelii*	1	0.6	3.8	MINV	CR	CR
33	*Lopholatilus villarii*	1	0.6	3.8	MCAR	NE	VU
34	*Cynoscion guatucupa*	1	0.6	3.7	MCAR	NE	LC
35	*Pagrus pagrus*	1	0.6	3.9	MINV	LC	DD
36	*Sarda sarda*	1	0.6	4.5	MCAR	LC	LC

Trophic categories: Macrocarnivore (MCAR); Mobile Invertivore (MINV); Omnivore (OMNI); Planktivore (PLANK); Herbivore (HERB). IUCN categories: Critically endangered (CR); Vulnerable (VU); Near threatened (NT); Least concern (LC); Data deficient (DD); Not evaluated (NE).

The same declining trend reported by fishers for *P. saltatrix*, *T. lepturus*, *E. marginatus*, *C. hippos*, and *S. fasciata* (Fig 3a) were revealed by fisheries landing data (Fig 4). Since the 1990s, *P. saltatrix* stocks declined significantly (i.e. approximately 47%) (Fig 4a), this status being perceived by fishers across all experience categories. For *T. lepturus*, the

landing declined significantly after the 2000s (Fig 4b), pattern promptly identified by the majority of fishers in less experienced categories. This species has been considered overexploited and also identified as a new target species in the region. Landing data show that since the 1990s, *E. marginatus* declined 61%, *C. hippos* diminished by 88%, and *S. fasciata* by 89%, the latter showed significant declining trends (Fig 4c, e, g). This overexploitation scenario was observed by very experienced fishers, which cited the first two species as overexploited in a higher proportion (Fig 3a).

Fig 4. Artisanal fisheries (tons) landings in Arraial do Cabo from 1992 to 2008. Species on the left (a, c, e, g) correspond to those most frequently cited as overexploited in interviews and on the right (d, f, h), new target species reported by local fishers. (b) *Trichiurus lepturus* corresponds to both an overexploited and a new target species. a, b, d, f, g and h, are second-order polynomial regressions; c and e, an exponential regression.

The landings data of the new target species exhibited an increase from the 2000s onwards, followed by a reduction in the quantities of landed fish over the years (Fig 4b, d, f, h). From the 1990s to 2000s, the landings of new target species as *T. lepturus*, *C. orbignyanus*, and *A. monoceros* had an increase (Fig 4b, d, h). By the mid-2000s, landings of these species declined 69%, 55% and 85%, respectively (Fig 4b, d, h). Fishers also reported this trend, when respondents mentioned these species as overexploited (Fig 3b; Table 1). Landings of *P. arenatus* had a significant increase from 2000 onwards (Fig 4f), mainly reported by less experienced fishers (Fig 3b).

Catch MTL of TL ≥ 3.5 and TL > 4 species declined significantly from 1992 to 2008 ($r^2 = 0.26$; $P = 0.03$ and $r^2 = 0.43$; $P < 0.001$, respectively) (Fig 5a, b). When MTL of all landed species are represented, there were non-significant trends in MTL over time ($r^2 = 0.06$; $P = 0.32$). For species with TL < 3.5, the increasing trend was non-significant ($r^2 = 0.07$; $P = 0.14$) (Fig 5a, b).

In the years of 1995 and 1999, there were peaks in landings of all species in Arraial do Cabo (Fig 5c). However, between 2001 and 2004, there were sharp declines in landings for all groups (Fig 5b). Declining trends were also identified for species with TL > 4 through landing data. For the top 10 species, we observed a decrease in landings between 1992 and 2008 (Fig 5d).

Fig 5. Trends in MTL of species landed in Arraial do Cabo from 1992 to 2018. (a) Linear regression of catch MTL; (b) Landing data in tons; (c) Third - order polynomial regression of landings (in tons) of species with TL > 4; and (d) Landings of the top 10 landed species with TL> 4. The colors represent MTL calculated after excluding all species below trophic level 4 (blue), calculated after excluding all species below trophic level 3.5 (yellow), calculated with all species landed (red), and for species with trophic level below 3.5 (green).

When contrasting the MTL of species mentioned by fishers as overexploited and as new target species, we observed that the citations of overexploited species were more frequently within higher TL species relative to new target ones (S1 Fig). In addition, the MTL observed for overexploited species was higher than expected by chance (S1 Fig).

Fishers mentioned 35 different causes of fisheries overexploitation in Arraial do Cabo (1.58 ± 1.08, mean ± standard deviation). The five most mentioned causes were the increased fishing effort (22%), which was recognized by fishers as an increase in the number of fishers and boats; presence of industrial fishing vessels (21%), presence of purse seine fisheries (19%), gillnet fishing (16%), and trawling (12%).

Discussion

Our study revealed a replacement of target species identified by fisher's knowledge and landing data in a Brazilian secular fishing village. Here, we showed an overexploitation scenario of large and valuable fish species and their replacement by new target species. The decline of species stocks such as *P. saltatrix, E. marginatus, C. hippos* and *S. fasciata* were followed by sequential substitutions by less commercially valuable but more abundant species (e.g., *T. lepturus, B. capriscus, A. monocerus* and *P. arenatus*). Temporal changes in the landing composition of fish species have been reported around the world [29,79–81]. In general, changes are from large-bodied species belonging to high trophic levels to smaller and lower trophic level species [10], and/or replacement of fish to invertebrate species [29]. In southern Brazil, a study used fishing landings, market values and multispecies indicators including MTL to evaluate changes and fishing impacts over time [82]. As in our study, authors did not identify declines in MTL of landings. However, they report the decline of most high trophic level species, as sharks, indicated from market data and landing volumes. This study also suggests that changes based on a single indicator such as MTL can be masked, and the use of multiple approaches can make these changes more explicit [82]. In this context, our study is the first to indicate temporal changes in the composition of fishing resources by combining fisher's knowledge and landing data in Southeastern Brazilian Coast.

Our results show that fisher's perception of overexploited and new target species is shaped by their fishing experience. As experience increases with years of fishing practice, fishers are able to recognize a greater number of overexploited and new target species. These findings indicate the occurrence of a shifted baseline among fishers' generations, which in this case, is the loss in fisher perception regarding changes in fisheries' composition and the decline of fish species throughout generations [50]. At the time young and less experienced fishers started fishing, several species were already overexploited, compromising their current perception of fish stocks. The small number of species reported by less experienced fishers compared with experienced ones may reflect the lack of familiarity and/or a reduced interaction of fishers with the natural environment [49]. Such

disconnection with past conditions has effects on the willingness and motivation of new generations to accept management restrictions aimed to restore resources and even mislead recovery perceptions, the "placebo effect" [83]. In Arraial do Cabo, the overexploitation of fishing resources [21,54] has made less experienced fishers look for alternative income sources. Several less experienced fishers have mentioned being involved in tourism related activities, using their boats for sightseeing, as a parallel activity to fishing. This reduces the engagement of young people with fishing and possibly impairs their perception of the status of fishing resources. The loss of familiarity with the natural environment of future generations can result in the absence of past information or experience with historical conditions [49], and members of each new generation accept the scenario in which they were raised as being normal.

Our results revealed declining trends in landings of commercially important mesopredators in Arraial do Cabo. Declines of high trophic species like groupers and bluefish have been reported over the last decades [54] . *P. saltatrix* and *E. marginatus*, one being pelagic and one a reef-associated, are important resources in Arraial do Cabo, as well as along the south and southeastern Brazilian coast [84]. Our findings highlight that very experienced fishers have identified the overexploited status of *E. marginatus* and *C. hippos* populations in a greater proportion. More experienced fishers have reported higher catches of target species than less experienced ones [17,20,53,60,73,85], this being a common scenario of overexploitation in small-scale fisheries since fish resources are decreasing. For instance, decades ago, groupers were abundant in Arraial do Cabo, and generations of more experienced fishers witnessed large catches of the species in the past. Today, older fishers perceive this decline, and catches of groupers have become rare events in the region. Due to life history traits such as high longevity, slow growth, late maturation, and low reproductive rates, groupers are highly sensitive to fishing effects [86,87]. Such characteristics have contributed to the threatened status of many grouper species worldwide [15,88]. The decline

of other predator species as *C. hippos* and *S. fasciata* is concerning, since these species are important regional fishing resources [89], and as expected for large carnivores, depict a combination of biological traits that can enhance their extinction risk [90].

Large catches of *T. lepturus* in Arraial do Cabo occurred incidentally in the past, i.e. when fishers targeted other species with high commercial value such as *P. saltatrix* and *E. marginatus*. The largehead hairtail was identified both as overexploited and new target species, mostly by less experienced fishers. Fishing of this species is more recent than of other fishes mentioned as overexploited. Thus, knowledge about the exploitation status of this species is more widespread among young generations. Due to its low commercial value and lack of consumer market, large quantities of *T. lepturus* were previously discarded. Later on, the decline of large predators in the region [21,54], led fishers to replace their target species. This has made *T. lepturus* economically valuable and a target of local fisheries, which was also observed for *B. capriscus, C. orbignyanus* and *A. monoceros*. Fishing directed at other less valuable species has become an alternative to maintain local fishing activities. The collapse of higher trophic level species caused by overfishing led fishers to target less valuable and lower trophic level species, similar trends are found in case studies that report the occurrence of fishing down marine food webs [29].

The fishing down the food web process predicts continuous declines in catches of high trophic level and a shift to small, low-trophic level species [27]. This ubiquitous phenomenon has been identified in several ecosystems, both at local and global scales [30,39,67,91,92, see more case studies in www.fishingdown.org]. Here, we demonstrate the scenario of overexploitation of high trophic species caught in Arraial do Cabo [54], combining alternative data sources from fishers' LEK and landing data (from 1992 to 2008), supported by other local studies [54]. We observed a declining trend in the mean trophic level and landings of species with $TL > 4$ and $TL \geq 3.5$. When considering the MTL of all species landed, there is

no clear pattern in trends, probably due to the decrease in landings of species of low trophic level, such as sardines, and the significant declines in landings of large predators. The same case has been identified in studies in upwelling systems in the Mediterranean Sea [93,94]. Studies point out that in some cases fishing down may not be identified [39], due to several factors that are responsible for masking the prevalence of this process [39,95], including taxonomic over-aggregation, the body size of species and geographical expansion of fisheries [33]. Thus, studies suggest the use of MTL as a complementary tool for the assessment of the effects of fishing on ecosystems [39,96].

Fishers LEK revealed that, in the past, large catches of tunas, sharks and groupers were common in Arraial do Cabo (Fig 6). Shark species such as the spinner shark (*Carcharhinus brevipinna*) were caught in large quantities (Fig 6b), as well as the currently threatened Brazilian guitarfish (*Pseudobatos horkelii*) and the sandbar shark (*Carcharhinus plumbeus*) [54]. Despite never being a major fishing target, these shark species are still caught in the region, but in smaller quantities. The current fishing scenario revealed in the present study is quite different from that reported by the old fishers. Despite its low trophic level, the large body size of parrotfishes such as the endemic greenbeak parrotfish (*Scarus trispinosus*) has made this species a major target of spearfishing in the Southwestern Atlantic [54,97,98]. In Arraial do Cabo, a past study had identified reductions in the abundance and body size of *S. trispinosus* individuals [54]. In addition, spearfishers commonly catch other large parrotfishes as those of the *Sparisoma* genus, indicating shifts in the composition of target species [54].

Fig 6. Photographs showing past and present catches of species mentioned by fishers as overexploited and new target species in Arraial do Cabo: (a) *Epinephelus marginatus* in 1982; (b) *Carcharhinus*

brevipinna caught in Praia Grande in 2005; (c) *Balistes capriscus* and (d) beach seine fishing at Praia Grande recorded in 2020.

As we expected, increase in fishing effort was described by fishers as the main cause of overexploitation. While local reports have revealed a declining trend in the number of boats (S2 Fig a, S3 Table). Due to the overexploited state of fish stocks, fishers have to spend more time fishing to obtain the same yields as in the past. Another cause of overexploitation reported by fishers was purse seine fisheries. This fishing gear has a high catch potential, and it is less selective when compared to other artisanal fishing gear (e.g. line and hook). For example, in 2019, purse seine catches represented 59% (231 kg) of the total landed catch in the region (S2 Fig). In addition, purse seine effort in fishing days corresponded to only 6% of the total fishing days (S2 Fig). Therefore, despite changes in fishing effort, the scenario of species overexploitation is worrisome and can be even greater than reported in our study.

The few existent management initiatives in the region, which rule over spawning periods of few species, have been ineffective to change the decline trends of fishing resources in Arraial do Cabo. Classical management strategies as minimum landing size of capture, closure periods, fishing quotas with quantities determined after stock assessments should be implemented based on national conservation status of species. We believe that such strategy may be viable through an adaptive comanagement framework involving stakeholders in all steps of the process. Management may be real-time informed by the small-scale fisheries monitoring that occur in that marine extractive reserve. Threatened species as *E. marginatus, Hyporthodus niveatus, Lopholatilus villarii,* and *Sparisoma axillare*, which were mentioned by fishers as overexploited and new target species (Table 1 and 2), should not be targeted until stocks are recovered. As an alternative to minimize illegal fishing off the season and the incidental catch of juveniles, resource users should be oriented to target healthy fish stocks or

to search for alternative income sources such as community-based tourism initiatives. A previous study has compared the effects of partially protected areas vs. unprotected areas in the abundance and body size of reef fish, including a site in Arraial do Cabo [99]. Groupers had higher density and relative abundance in a partially protected area, indicating that even small no-take areas can help recover fish populations [99]. The case of Arraial do Cabo is not different from many reef sites around the world, where the fishing effort has surpassed the recovery potential of natural resources [100]. The good news is that even at relatively small areas groupers can increase in density and size if fishing ceases [99]. Additional ongoing acoustic telemetry study in the region indicate relatively small home ranges (0.05 km^2) for two vulnerable parrotfishes (*Sparisoma frondosum* and *S. axillare*) [101], which have implications for establishing the minimum size of no-takes areas (authors, per. com.). Small no-take areas appear as a critical tool to preserve local resources, but more importantly, to show local users their efficiency in rebuilding stocks. Additionally, local fishers can be empowered to explore future established no-take areas through tourism initiatives, having exclusivity over local tourism agencies.

We also highlight the need for greater inspection by the authorities to banning illegal fishing by industrial vessels such as trawl and gillnet fisheries that occur even within the MPA. Encourage dialogue between fishers, especially older and younger generations, so that the elders transmit their knowledge on the past of local fisheries, helping young generations to understand the abundance trends of fish resources. The knowledge of how marine resources were more abundant in the past is linked with higher willingness to collaborate with management and conservation initiatives. This may also influence the overcome of the shifting baseline syndrome among fishers.

Here we demonstrated that experienced fishers better recognize changes in fisheries and in marine ecosystems when compared to less experienced ones. In addition, we identified

changes in composition and abundance of pelagic and demersal, reef-associated fish species caught in a secular fishing village using fishers' local ecological knowledge and fisheries landing data. Despite the short time series, fishing monitoring data reveals declining trends in stocks of mesopredators species overtime. The combination of multiple sources of information can help prevent biases as underestimated assessments of the impact of fishing [102]. By incorporating past data, it is possible to identify changes in catches over time, as well as to adjust conservation goals and strategies for marine ecosystems.

Acknowledgments

COF. acknowledges Coordenação de Aperfeiçoamento de Pessoal de Nível Superior (CAPES) for financial support. VJG. received a post-doctoral grant #2017/22273-0, São Paulo Research Foundation (FAPESP). CELF is supported by grants from FAPERJ and CNPq. Costão Rochoso Project (FUNBIO Grant Pesquisa Marinha 020/2017) has provided financial, and logistic support during field sampling in Arraial do Cabo. This work is part of a research program supported by Serrapilheira Institute (grant number Serra-1708-15364) awarded to GO Longo (PI). In addition, we thank fishers for their collaboration and shared knowledge. ICMBio has ever been supportiveness for our projects.

References

1. Worm B, Barbier EB, Beaumont N, Duffy JE, Folke C, Halpern BS, et al. Impacts of biodiversity loss on ocean ecosystem services. Science. 2006; 314 (5800): 787-790.

2. Harnik PG, Lotze HK, Anderson SC, Finkel ZV, Finnegan S, Lindberg DR et al. Extinctions in ancient and modern seas. Trends Ecol Evol. 2012; 27 (11): 608-617.

3. Jackson JBC, Kirby MX, Berger WH, Bjorndal KA, Botsford LW, Bourque BJ, et al. Historical overfishing and the recent collapse of coastal ecosystems. Science. 2001; 293 (5530): 629-637.

4. Jennings S, Kaiser MJ. The effects of fishing on marine ecosystems. In Advances in Marine Biology. Academic Press. 1998; 34, pp. 201-352.

5. Pauly D, Zeller D. Catch reconstructions reveal that global marine fisheries catches are higher than reported and declining. Nat Commun. 2016; 7, 10244.

6. FAO. The State of World Fisheries and Aquaculture 2020. Sustainability in action. Rome. 2020.

7. Knowlton N. Ocean Optimism: Moving Beyond the Obituaries in Marine Conservation. Annu Rev Mar Sci. 2020; 13.

8. Hilborn R, Amoroso RO, Anderson CM, Baum JK, Branch TA, Costello C, et al. Effective fisheries management instrumental in improving fish stock status. PNAS. 2020; 117 (4): 2218-2224.

9. Duarte CM, Agusti S, Barbier E, Britten GL, Castilla JC, Gattuso JP et al. Rebuilding marine life. Nature. 2020; 580 (7801): 39-51.

10. Stevens JD, Bonfil R, Dulvy NK, Walker PA. The effects of fishing on sharks, rays, and chimaeras (chondrichthyans), and the implications for marine ecosystems. ICES J Mar Sci. 2000; 57 (3): 476-494.

11. Shin YJ, Rochet MJ, Jennings S, Field JG, Gislason H. Using size-based indicators to evaluate the ecosystem effects of fishing. ICES J Mar Sci. 2005; 62 (3): 384-396.

12. Coll M, Carreras M, Ciércoles C, Cornax MJ, Gorelli G, Morote E, et al. Assessing fishing and marine biodiversity changes using fishers' perceptions: the Spanish Mediterranean and Gulf of Cadiz case study. PLoS One. 2014; 9 (1): e85670.

13. Peñaherrera-Palma C, Putten IV, Karpievitch YV, Frusher S, Llerena-Martillo Y, Hearn AR, et al. Evaluating abundance trends of iconic species using local ecological knowledge. Biol Conserv. 2018; 225, 197-207.

14. Shackell NL, Frank KT, Fisher JA, Petrie B, Leggett WC. Decline in top predator body size and changing climate alter trophic structure in an oceanic ecosystem. Proc Royal Soc B. 2010; 277 (1686): 1353-1360.

15. Heithaus MR, Frid A, Wirsing AJ, Worm B. Predicting ecological consequences of marine top predator declines. Trends Ecol Evol. 2008; 23 (4): 202-210.

16. Sandin SA, Walsh SM, Jackson JB. Prey release, trophic cascades, and phase shifts in tropical nearshore ecosystems. Trophic Cascades: predators, prey, and the changing dynamics of nature. 2010; 71-90.

17. McClenachan L. Historical declines in goliath grouper in south Florida. Endang Species Res. 2009; 7 (3): 175-181.

18. Venkatachalam AJ, Price ARG, Chandrasekara S, Sellamuttu SS, Kaler J. Changes in frigate tuna populations on the south coast of Sri Lanka: evidence of the shifting baseline syndrome from analysis of fisher observations. Aquat Conserv Mar Freshwater Ecosyst. 2010; 20 (2): 167-176.

19. Luiz OJ, Edwards AJ. Extinction of a shark population in the Archipelago of Saint Paul’s Rocks (equatorial Atlantic) inferred from the historical record. Biol Conserv. 2011; 144 (12): 2873-2881.

20. Giglio VJ, Luiz OJ, Gerhardinger LC. Depletion of marine megafauna and shifting baselines among artisanal fishers in eastern Brazil. Anim Conserv. 2015; 18 (4): 348-358.

21. Giglio VJ, Bender MG, Zapelini C, Ferreira CEL. The end of the line? Rapid depletion of a large-sized grouper through spearfishing in a subtropical marginal reef. Perspect Ecol Conserv. 2017; 15 (2): 115-118.

22. Lewison RL, Crowder LB, Read AJ, Freeman SA. Understanding impacts of fisheries bycatch on marine megafauna. Trends Ecol Evol. 2004; 19 (11): 598-604.

23. Myers RA, Baum JK, Shepherd TD, Powers SP, Peterson CH. Cascading Effects of the Loss of Apex Predatory Sharks from a Coastal Ocean. Science. 2007; 315 (5820): 1846-1850.

24. Baum JK, Worm B. Cascading top down effects of changing oceanic predator abundances. J Animal Ecol. 2009; 78 (4): 699-714.

25. Essington TE, Beaudreau AH, Wiedenmann J. Fishing through marine food webs. Proc Natl Acad Sci USA. 2006; 103 (9): 3171-3175.

26. Heithaus MR, Frid A, Vaudo JJ, Worm B, Wirsing AJ 2010. Unraveling the ecological importance of elasmobranchs. In: Carrier JC, Musick J, Heithaus MR (eds) Biology of Sharks and their relatives II. CRC Press, Boca Raton. 611-637.

27. Pauly D, Christensen V, Dalsgaard J, Froese R, Torres Jr F. Fishing down marine food webs. Science. 1998; 279 (5352): 860-863.

28. Rogers SI, Ellis JR. Changes in the demersal fish assemblages of British coastal waters during the 20th century. ICES J Mar Sci. 2000; 57 (4): 866-881.

29. Molfese C, Beare D, Hall-Spencer JM. Overfishing and the Replacement of Demersal Finfish by Shellfish: An Example from the English Channel. PLoS One. 2014; 9 (7): e101506.

30. Pauly D, Palomares ML, Froese R, Sa-a P, Vakily M, Preikshot D, et al. Fishing down Canadian aquatic food webs. Can J Fish Aquat Sci. 2001; 58 (1): 51-62.

31. Pauly D, Watson R. Background and interpretation of the ‘Marine Trophic Index’ as a measure of biodiversity. Philos Trans R Soc B. 2005; 360 (1454): 415-423.

32. Freire KMF, Pauly D. Fishing down Brazilian marine food webs, with emphasis on the east Brazil large marine ecosystem. Fish Res. 2010; 105 (1): 57-62.

33. Liang C, Pauly D. Fisheries impacts on China's coastal ecosystems: Unmasking a pervasive ‘fishing down’ effect. PLoS One. 2017; 12 (3): e0173296.

34. Erlandson JM, Rick TC, Braje TJ. Fishing up the Food Web?: 12,000 Years of Maritime Subsistence and Adaptive Adjustments on California’s Channel Islands. Pac Sci. 2009; 63 (4): 711-724.

35. Litzow MA, Urban D. Fishing through (and up) Alaskan food webs. Can J Fish Aquat Sci. 2009; 66 (2): 201-211.

36. Branch TA. Fishing Impacts on Food Webs: Multiple Working Hypotheses. Fisheries. 2015; 40: 373-375.

37. Sethi SA, Branch TA, Watson R. Global fishery development patterns are driven by profit but not trophic level. PNAS. 2010; 107, 12163-12167.

38. Branch TA, Watson R, Fulton EA, Jennings S, McGilliard CR, Pablico GT, et al. The trophic fingerprint of marine fisheries. Nature, 2010; 468 (7322): 431-435.

39. Stergiou K, Tsikliras A. Fishing down, fishing through and fishing up: fundamental process versus technical details. Mar Ecol Prog Ser. 2011; 441: 295-301.

40. Salas S, Chuenpagdee R, Seijo JC, Charles A. Challenges in the assessment and management of small-scale fisheries in Latin America and the Caribbean. Fish Res. 2007; 87 (1): 5-16.

41. Begossi A, Silvano RAM. Ecology and ethnoecology of dusky grouper [garoupa, *Epinephelus marginatus* (Lowe, 1834)] along the coast of Brazil. J Ethnobiol Ethnomed. 2008; 4 (1): 20.

42. Lima EG, Begossi A, Hallwass G, Silvano RAM. Fishers' knowledge indicates short-term temporal changes in the amount and composition of catches in the southwestern Atlantic. Mar Policy. 2016; 71, 111-120.

43. Sáenz-Arroyo A, Roberts CM, Torre J, Cariño-Olvera M, Hawkins JP. The value of evidence about past abundance: marine fauna of the Gulf of California through the eyes of 16th to 19th century travellers. Fish Fish. 2006; 7 (2): 128-146.

44. Martins IM, Medeiros RP, Domenico MD, Hanazaki N. What fishers' local ecological knowledge can reveal about the changes in exploited fish catches. Fish Res. 2018; 198, 109-116.

45. Giglio VJ, Suhett AC, Zapelini CS, Ramiro AS, Quimbayo JP. Assessing captures of recreational spearfishing in Abrolhos reefs, Brazil, through social media. Reg Stud Mar Sci. 2020; 34, 100995.

46. Braga HO, Azeiteiro UM, Oliveira HMF, Pardal MA. Conserving Brazilian Sardine: Fisher's attitudes and knowledge in the Marine Extractive Reserve of Arraial do Cabo, Rio de Janeiro State, Brazil. Fish Res. 2018; 204, 402-411.

47. Taylor SM, Braccini JM, Bruce BD, McAuley RB. Reconstructing Western Australian white shark (*Carcharodon carcharias*) catches based on interviews with fishers. Mar Freshwater Res. 2018; 69 (3): 366-375.

48. Baum JK, Myers RA. Shifting baselines and the decline of pelagic sharks in the Gulf of Mexico. Ecol Lett. 2004; 7 (2): 135-145.

49. Soga M, Gaston KJ. Shifting baseline syndrome: causes, consequences, and implications. Front Ecol Environ. 2018; 16 (4): 222-230.

50. Pauly D. Anecdotes and the shifting baseline syndrome of fisheries. Trends Ecol Evol. 1995; 10 (10): 430.

51. Papworth SK, Coad JRL, Milner- Gulland EJ. Evidence for shifting baseline syndrome in conservation. Conserv Lett. 2009; 2 (2): 93-100.

52. Thurstan RH, McClenachan L, Crowder LB, Drew JA, Kittinger JN, Levin PS. Filling historical data gaps to foster solutions in marine conservation. Ocean Coastal Manage. 2015; 115, 31-40.

53. Bender MG, Floeter SR, Hanazaki N. Do traditional fishers recognise reef fish species declines? Shifting environmental baselines in Eastern Brazil. Fish Manag Ecol. 2013; 20 (1): 58-67.

54. Bender MG, Machado GR, Azevedo PJS, Floeter SR, Monteiro-Netto C, Luiz OJ, et al. Local Ecological Knowledge and Scientific Data Reveal Overexploitation by Multigear Artisanal Fisheries in the Southwestern Atlantic. PloS One. 2014; 9 (10): e110332.

55. Reis-Filho JA, Freitas RHA, Loiola M, Leite L, Soeiro G, Oliveira HHQ, et al. Traditional fisher perceptions on the regional disappearance of the largetooth sawfish *Pristis pristis* from the central coast of Brazil. Endang Species Res. 2016; 29 (3): 189-200.

56. Silvano RAM, MacCord PFL, Lima RV, Begossi A. When Does this Fish Spawn? Fishermen's Local Knowledge of Migration and Reproduction of Brazilian Coastal Fishes. Environ Biol Fishes. 2006; 76 (2-4): 371-386.

57. Silvano RAM, Begossi A. Fishermen's local ecological knowledge on Southeastern Brazilian coastal fishes: contributions to research, conservation, and management. Neotr Ichthyol. 2012; 10 (1): 133-147.

58. Barbosa-Filho MLV, Schiavetti A, Alarcon DT, Costa-Neto EM. "Shark is the man!": ethnoknowledge of Brazil's South Bahia fishermen regarding shark behaviors. J Ethnobiol Ethnomed. 2014; 10 (1): 54.

59. Silvano RAM, Begossi A. What can be learned from fishers? An integrated survey of fishers' local ecological knowledge and bluefish (Pomatomus saltatrix) biology on the Brazilian coast. Hydrobiologia. 2010; 637(1), p.3.

60. Sáenz-Arroyo A, Roberts CM, Torres J, Cariño-Olvera M. Using fishers' anecdotes, naturalists' observations and grey literature to reassess marine species at risk: the case of the Gulf grouper in the Gulf of California, Mexico. Fish Fish. 2005; 6 (2): 121-133.

61. Bevilacqua AHV, Carvalho AR, Angelini R, Christensen V. More than Anecdotes: Fishers' Ecological Knowledge Can Fill Gaps for Ecosystem Modeling. PLoS One. 2016; 11 (5): e0155655.

62. Sánchez-Jiménez A, Fujitani M, MacMillan D, Schlüter A, Wolff M. Connecting a Trophic Model and Local Ecological Knowledge to Improve Fisheries Management: The Case of Gulf of Nicoya, Costa Rica. Front. Mar. Sci. 2019; 6:126.

63. Gerhardinger LC, Godoy EAS, Jones PJS. Local ecological knowledge and the management of marine protected areas in Brazil. Ocean Coast Manage. 2009; 52 (3-4): 154-165.

64. Wiber MG, Young S, Wilson L. Impact of Aquaculture on Commercial Fisheries: Fishermen's Local Ecological Knowledge. Hum Ecol Interdiscip J. 2012; 40 (1): 29-40.

65. Damasio LDMA, Lopes PFM, Guariento RD, Carvalho AR. Matching Fishers' Knowledge and Landing Data to Overcome Data Missing in Small-Scale Fisheries. PLoS One. 2015; 10 (7): e0133122.

66. Aylesworth L, Phoonsawat R, Suvanachai P, Vincent ACJ. Gaenerating spatial data for marine conservation and management. Biodivers Conserv. 2017; 26 (2): 383-399.

67. Santos RO, Rehage JS, Kroloff EKN, Heinen JE, Adams AJ. Combining data sources to elucidate spatial patterns in recreational catch and effort: fisheries-dependent data and local

ecological knowledge applied to the South Florida bonefish fishery. Environ Biol Fishes. 2019; 102 (2): 299-317.

68. Melo JCF, Kruel VSF, Hanazaki N. Árvores e madeiras na cultura naval tradicional Bailey, KD. 1994. Methods of Social Research. New York: The Free Press/Macmillan Publishers. 588; 2019.

69. Carvalho WFD, Gonzalez Rodriguez E. Development of primary and bacterial productivity in upwelling waters of Arraial do Cabo region, RJ (Brazil). Braz J Oceanogr. 2004; 52 (1): 35-45.

70. Cordeiro CAMM, Mendes TC, Harborne AR, Ferreira CEL. Spatial distribution of nominally herbivorous fishes across environmental gradients on Brazilian rocky reefs. J Fish Biol. 2016; 89 (1): 939-958.

71. Mendonça, T.C.M., de Moraes, E.A., Costa, M.A.M., 2013. Turismo e pesca nas Reservas Extrativistas Marinhas de Arraial do Cabo (RJ) e da Prainha do Canto Verde (CE): possibilidades e limites de complementaridade. Caderno Virtual de Turismo. 2013; 13 (3), 372-390.

72. Bailey, KD, 1994. Methods of Social Research. New York: The Free Press/Macmillan Publishers. 588.

73. Maia HA, Morais RA, Siqueira AC, Hanazaki N, Floeter SR, Bender MG. Shifting baselines among traditional fishers in São Tomé and Príncipe islands. Ocean Coast Manage. 2018; 154, 133-142.

74. Pauly D, Froese R. Comments on FAO's State of Fisheries and Aquaculture, or ‘SOFIA 2010’. Mar Policy. 2012; 36 (3): 746-752.

75. Palomares MLD, Pauly D. Editors. 2020. SeaLifeBase. World Wide Web electronic publication. Available from: www.sealifebase.org, version (07/2020).

76. Dulvy NK, Sadovy Y, Reynolds JD. Extinction vulnerability in marine populations. Fish Fisheries. 2003; 4: 25-64.

77. Pauly D, Christensen V, Guénette S, Pitcher TJ, Sumaila UR, Walters CJ, et al. Towards sustainability in world fisheries. Nature. 2002; 418 (6898): 689-695.

78. R Development Core Team. 2019. R: A language and environment for statistical computing. 418 Vienna, Austria: R Foundation for statistical Computing.

79. Harris AN, Poiner IR. Changes in species composition of demersal fish fauna of Southeast Gulf of Carpentaria, Australia, after 20 years of fishing. Mar Biol. 1991; 111: 503-519.

80. Jin X, Tang Q. Changes in fish species diversity and dominant species composition in the Yellow Sea. Fisheries Research. 1996; 26: 337-352.

81. Fogarty MJ, Murawski SA. Large-scale disturbance and the structure of marine systems: fishery impacts on georges bank. Ecological Applications. 1998; 8: S6-S22.

82. Pincinato RBM, Gasalla MA. Priceless prices and marine food webs: Long-term patterns of change and fishing impacts in the South Brazil Bight as reflected by the seafood market. Progress in Oceanography. 2010; 87: 320-330.

83. Lovell S, Johnson AE, Ramdeen R, McClenachan L. Shifted baselines and the policy placebo effect in conservation. Oryx. 2018; 54 (3): 383-391.

84. Begossi A, Salivonchyk SV, Barreto T, Nora V, Silvano RAM. Small-scale Fisheries and Conservation of Dusky Grouper (Garoupa), *Epinephelus marginatus* (Lowe, 1834) in the Southeastern Brazilian Coast. Sci J Agric Res Manag. 2012; 2012:4.

85. Ferreira HM, Reuss-Strenzel GM, Alves JA, Schiavetti A. Local ecological knowledge of the artisanal fishers on *Epinephelus itajara* (Lichtenstein, 1822) (Teleostei: Epinephelidae) on Ilhéus coast-Bahia State, Brazil. J Ethnobiol Ethnomed. 2014; 10 (1): 51.

86. Seyboth E, Condini MV, Albuquerque CQ, Varela Jr AS, Velasco G, Vieira JP, et al. Age, growth, and reproductive aspects of the dusky grouper *Mycteroperca marginata* (Actinopterygii: Epinephelidae) in a man-made rocky habitat in southern Brazil. Neotrop Ichthyol. 2011; 9 (4): 849-856.

87. Condini MV, García-Charton JA, Garcia AM. A review of the biology, ecology, behavior and conservation status of the dusky grouper, *Epinephelus marginatus* (Lowe 1834). Rev Fish Biol Fish. 2018; 28 (2): 301-330.

88. Sadovy de Mitcheson Y, Craig MT, Bertoncini AA, Carpenter KE, Cheung WWL, Choat JH, et al. Fishing groupers towards extinction: a global assessment of threats and extinction risks in a billion dollar fishery. Fish Fish. 2013; 14(2): 119-136.

89. Duarte MR, Tubino RA, Monteiro-Neto C, Martins RRM, Vieira FC, Andrade-Tubino MF et al. Genetic and morphometric evidence that the jacks (Carangidae) fished off the coast of Rio de Janeiro (Brazil) comprise four different species. Biochem Syst Ecol. 2017; 71, 78-86.

90. Ceretta BF, Fogliarini CO, Giglio VJ, Maxwell MF, Waechter LS, Bender MG. Testing the accuracy of biological attributes in predicting extinction risk. Perspect Ecol Conserv. 2020; 18 (1): 12-18.

91. Bhathal B, Pauly D. ‘Fishing down marine food webs’ and spatial expansion of coastal fisheries in India, 1950-2000. Fish Res. 2008; 91 (1): 26-34.

92. Ding Q, Chen X, Yu W, Tian S, Chen Y. An evaluation of underlying mechanisms for “fishing down marine food webs”. Acta Oceanol Sin. 2016; 35 (8): 32-38.

93. Blanchard JL, Coll M, Trenkel VM, Vergnon R, Yemane D, Jouffre D, et al. Trend analysis of indicators: a comparison of recent changes in the status of marine ecosystems around the world. ICES J Mar Sci. 2010; 67 (4): 732-744.

94. Coll M, Shannon LJ, Yemane D, Link JS, Ojaveer H, Neira S, et al. Ranking the ecological relative status of exploited marine ecosystems. ICES J Mar Sci. 2010; 67 (4): 769-786.

95. Liang C, Pauly D. Masking and unmasking fishing down effects: The Bohai Sea (China) as a case study. Ocean Coast Manag 2020; 184, 105033.

96. Foley CMR. Management implications of fishing up, down, or through the marine food web. Mar Policy. 2013; 37: 176–182.

97. Francini-Filho RB, De Moura RL. Dynamics of fish assemblages on coral reefs subjected to different management regimes in the Abrolhos Bank, eastern Brazil. Aquat Conserv Mar Freshwater Ecosyst. 2008; 18 (7): 1166.

98. Roos NC, Pennino MG, De Macedo Lopes PF, Carvalho AR. Multiple management strategies to control selectivity on parrotfishes harvesting. Ocean Coast Manage. 2016; 134, 20-29.

99. Floeter SR, Halpern BS, Ferreira CEL. Effects of fishing and protection on Brazilian reef fishes. Biol Conserv. 2006; 128 (3): 391-402.

100. Fouzai N, Coll M, Palomera I, Santojanni A, Arneri E, Christensen V. Fishing management scenarios to rebuild exploited resources and ecosystems of the Northern-Central Adriatic (Mediterranean Sea). J Mar Syst. 2012; 102, 39-51.

101. MMA, 2018. Instituto Chico Mendes de Conservação da Biodiversidade. Livro Vermelho da Fauna Brasileira Ameaçada de Extinção: Volume VI - Peixes. In: Instituto Chico Mendes de Conservação da Biodiversidade (Org.). Livro Vermelho da Fauna Brasileira Ameaçada de Extinção. Brasília: ICMBio. 1232p. http://www.icmbio.gov.br. Download em 20 maio 2018.

102. Paterson B. Integrating fisher knowledge and scientific assessments. Anim Conserv. 2010; 13 (6): 536-537.

Supporting Information

S1 Table. Trophic level (TL) of species landed in Arraial do Cabo, from 1992 to 2008. Trophic levels were obtained from FishBase database where a higher value means higher trophic levels (Froese and Pauly, 2019).

S2 Table. Parameters of the Poisson Generalized linear model for the number of species cited by fishers based on years of practice.

S3 Table. Fishing effort of the artisanal fishing fleet of Arraial do Cabo, Rio de Janeiro, Brazil.

S1 Fig Differences in mean trophic level (MTL) of overexploited and new target species cited by local fishers. Observed MTL (red diamond) are contrasted against null MTL values (gray circles), generated through randomization of citation frequency of species within each category.

S2 Fig Fishing effort in number of boats (a) and fishing days (b) over the years for Arraial do Cabo, Rio de Janeiro, Brazil.

S1 File. Questionnaire applied in interviews with fishers in Arraial do Cabo.

Figure 1

Click here to access/download;Figure;Fig 1.tiff.tif

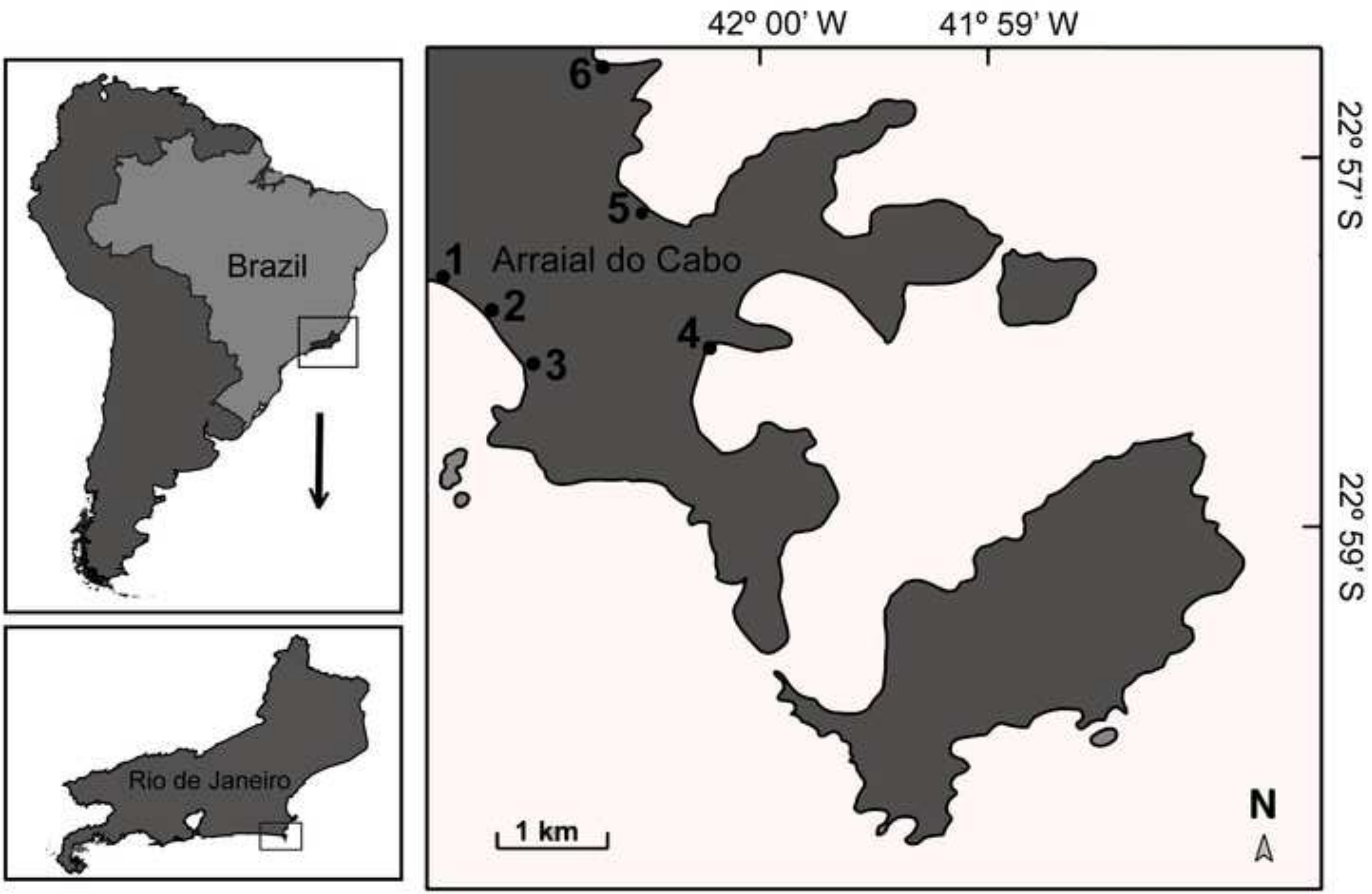

 Click here to access/download;Figure;Fig 2.tiff.tif

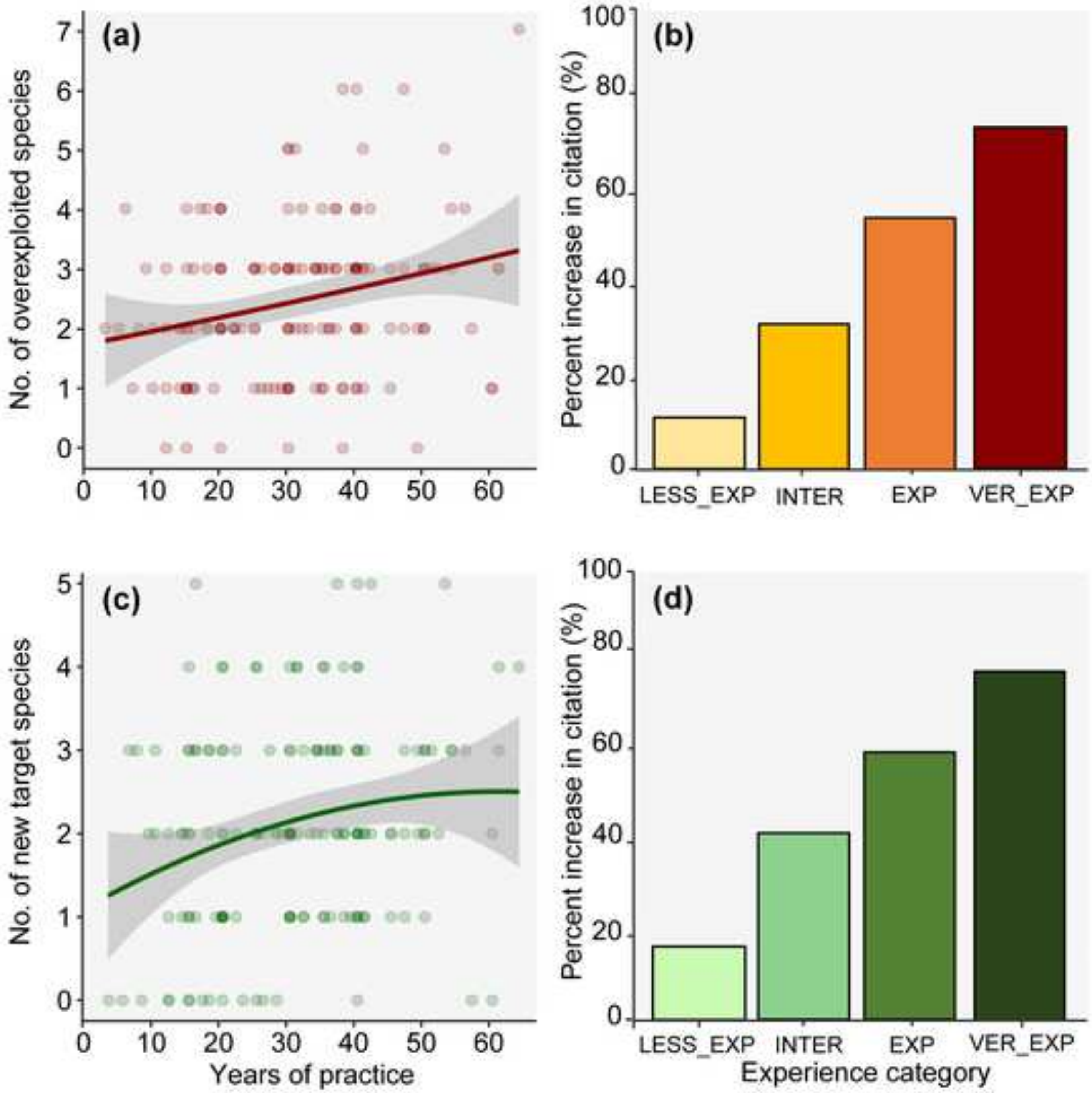

Figure 3

Click here to access/download;Figure;Fig 3.tiff.tif

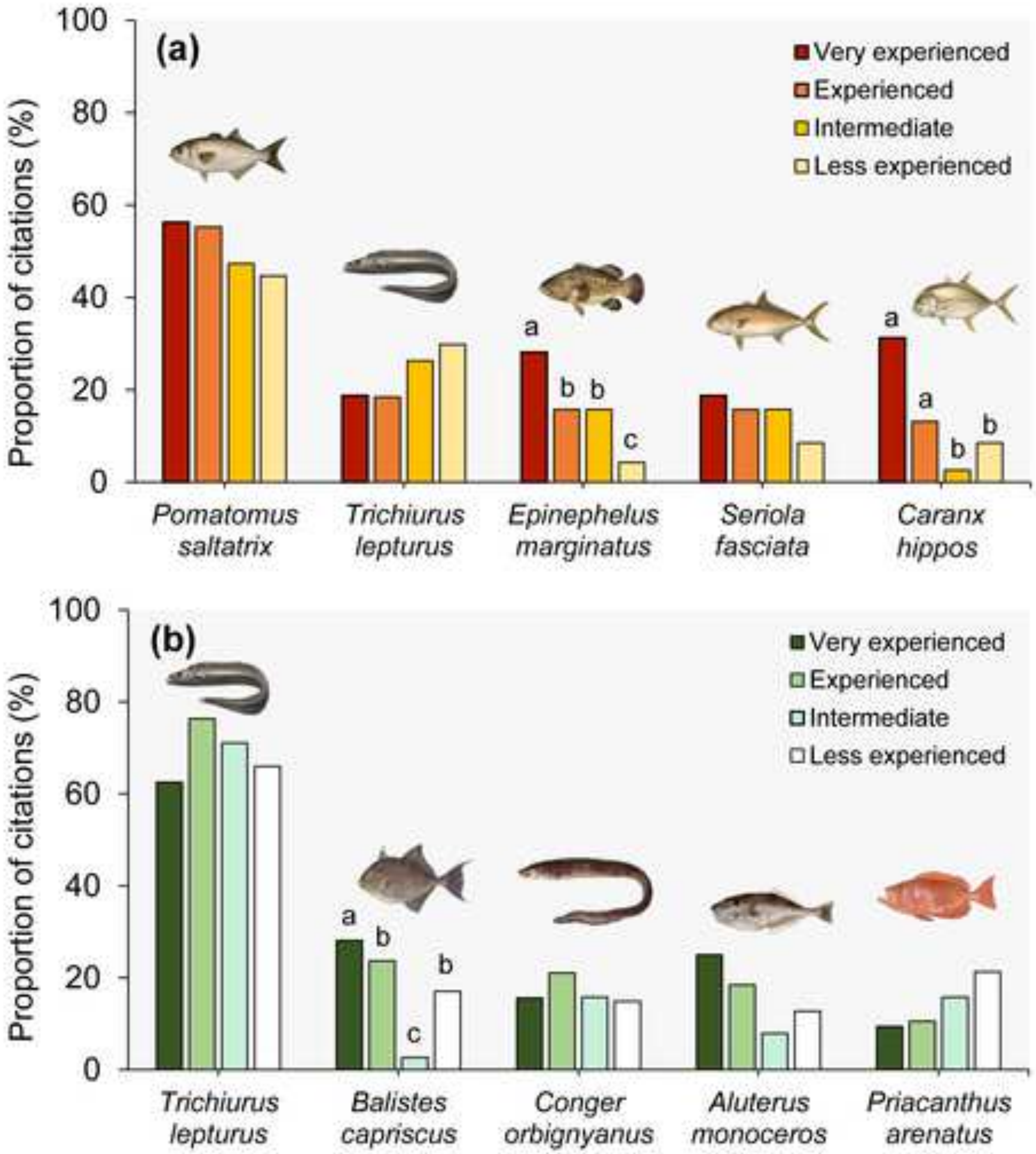

Figure 4

Click here to access/download;Figure;Fig 4.tiff.tif

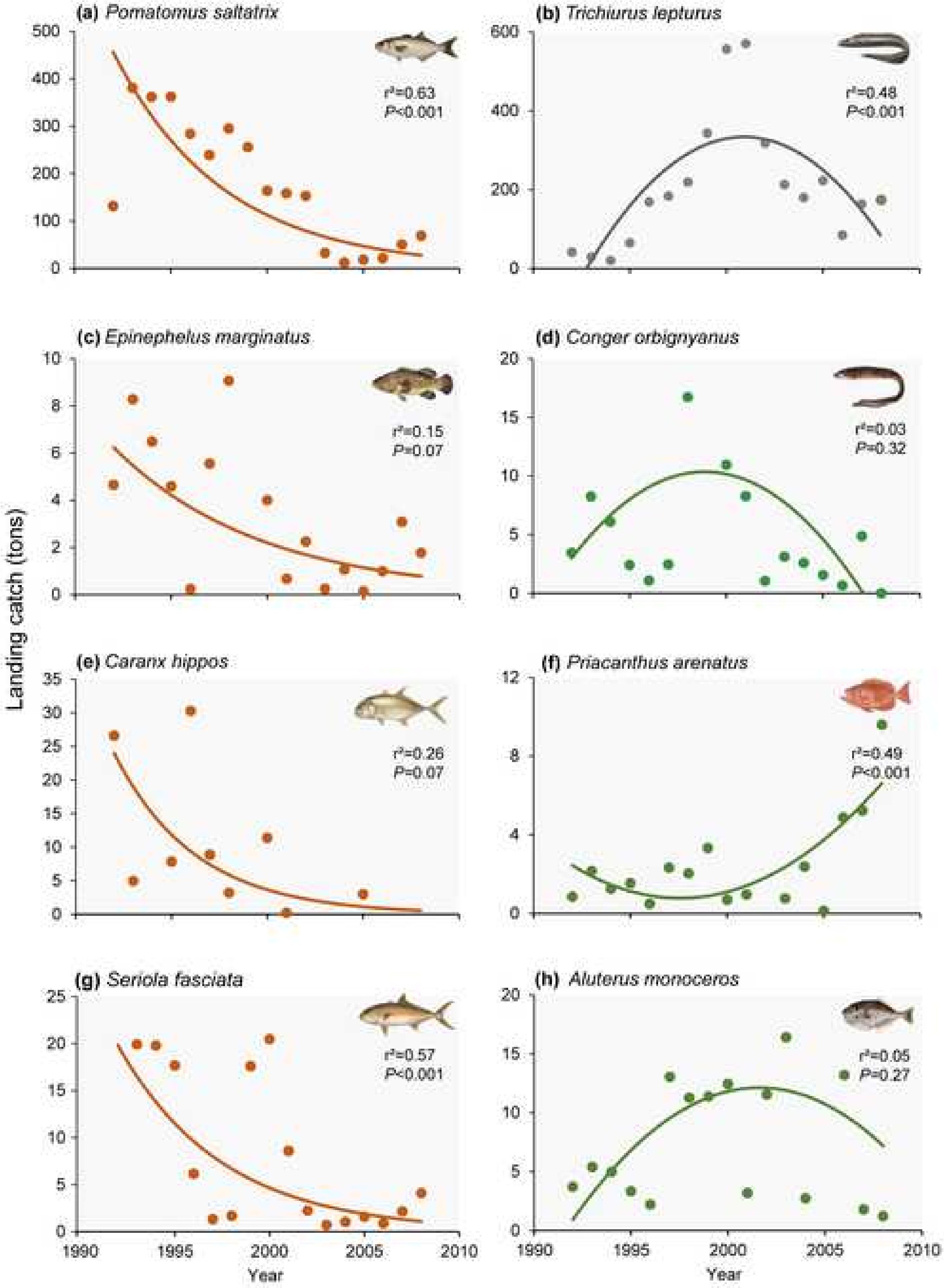

Figure 5

Click here to access/download;Figure;Fig 5.tiff.tif

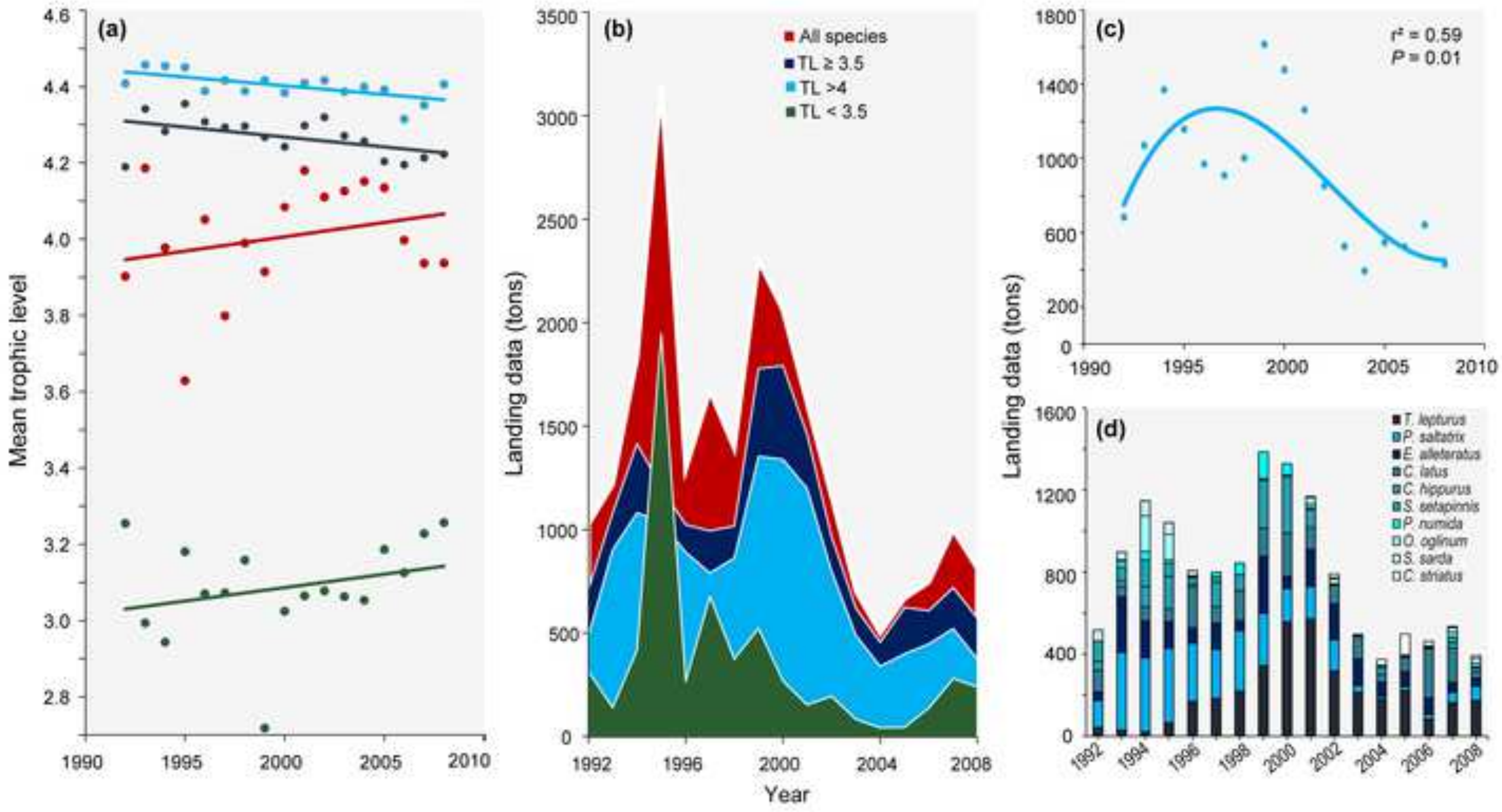

Figure 6

Click here to access/download;Figure;Fig 6.tiff.tif

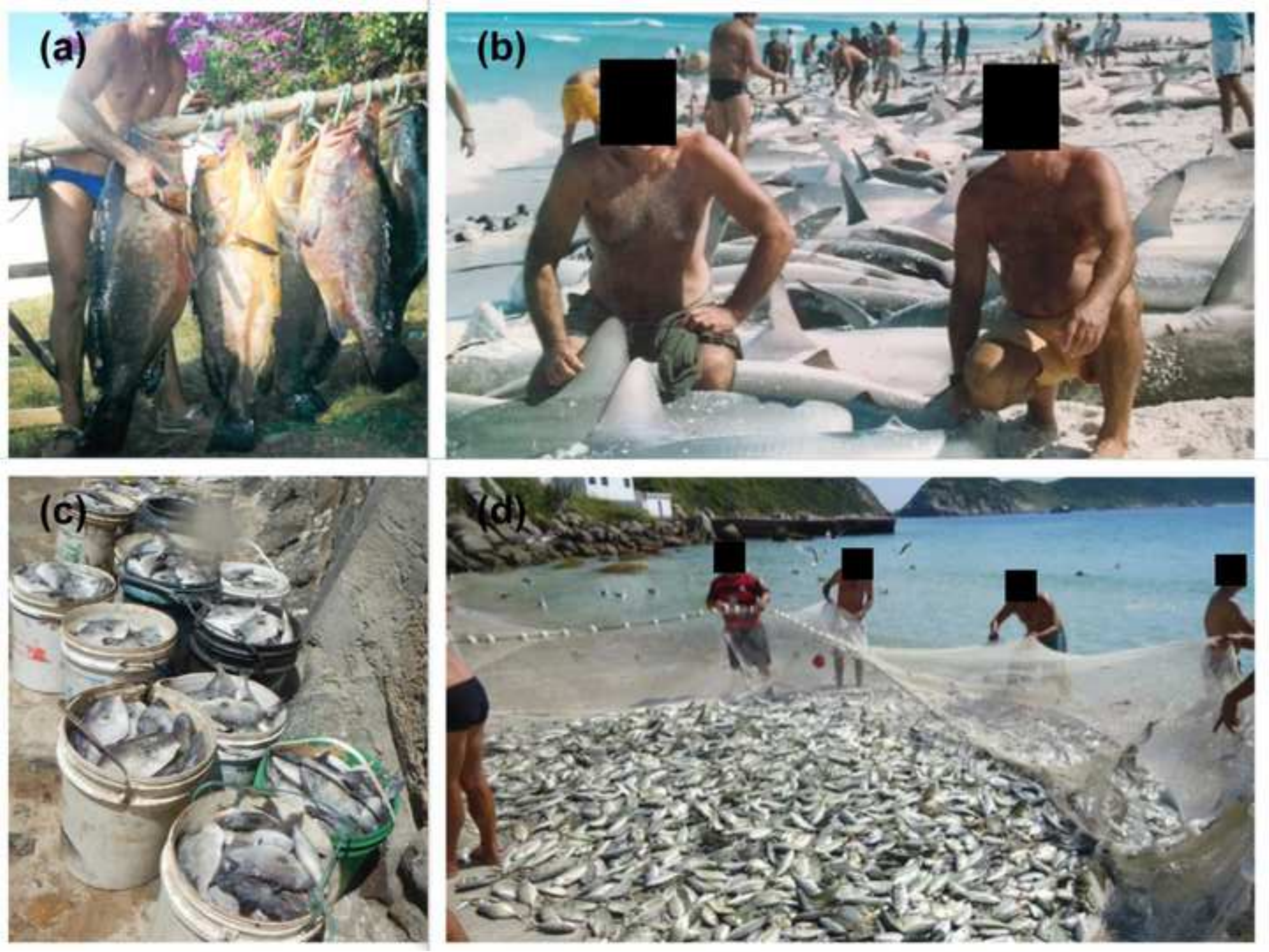

Click here to access/download
Supporting Information
Supporting information_Plos One.docx

Click here to access/download
Supporting Information - Compressed/ZIP File Archive
content-permission-form_MBender_assinado.pdf

Telling the same story: fishers and landing data reveal changes in fisheries on the Southeastern Brazilian Coast

Carine O. Fogliarini[a*], Carlos E.L. Ferreira[b], Jéssica Bornholdt[a], Moysés. C. Barbosa[b], Vinicius J. Giglio[c], Mariana G. Bender[a]

[a]Marine Macroecology and Conservation Lab, Departamento de Ecologia e Evolução, Universidade Federal de Santa Maria. Avenida Roraima, 1000, prédio 17 sala 1140, Bairro Camobi, Santa Maria, Rio Grande do Sul, Brazil.

[b]Reef Systems Ecology and Conservation Lab, Departamento de Biologia Marinha, Universidade Federal Fluminense, Niterói, RJ, Brazil.

[c]Marine Conservation and Ecology Lab, Instituto do Mar, Universidade Federal de São Paulo, Santos, São Paulo, Brazil.

* Corresponding author

E-mail: carine_fogliarini@hotmail.com

Abstract

An ~~complete~~ understanding of the effects of fishing on marine ecosystems relies on information about the ~~most~~ conserved state of these environments. Non-conventional approaches such as the use of historical data and local ecological knowledge can provide information and help adjust our references ~~regarding~~of changes in the environment~~environmental references~~. Also, the combination of different types of data can indicate a fisheries trend~~patterns~~ that would be undetectable when evaluated separately. Here we investigated changes in fisher's perceptions regarding overexploited and new target species in artisanal fisheries in a secular fishing village of the subtropical, southeastern Brazilian coast ~~Brazil~~. ~~Using landing data, w~~We identified temporal changes in landings ~~catches~~ and in the mean trophic level (MTL) of high trophic level species (≥ 3.5 and >4) over 16 years. Fishers' knowledge revealed shifts in perception associated with years of fishing practice. More experienced fishers recognized a greater number of overexploited and new target species than fishers in the beginning of their careers. Landing data has revealed declining ~~catch~~ trends of 72% for five ~~large-bodied~~ mesopredators species. Due to the overfishing of mesopredators, there was a shift in target species, towards ~~lower trophic level~~ fish that were previously discarded. ~~-~~Temporal changes in landings and in the MTL metric are concordant with previous reports on the overexploitation of species caught by local fishers~~Temporal changes in landings and in the MTL metric corroborated the occurrence of fishing down the web, previously identified through reports about overexploitation of species caught the replacement of target species by local fishers~~. Our work reveals that multiple sources of information can be combined to establish historical baselines and improve the detection of change in marine ecosystems.

Keywords: Shifting baseline, marine resources, overfishing, local ecological knowledge, fisheries monitoring~~landing data~~, fisheries management

Introduction

Marine and coastal ecosystems are globally threatened due to human impacts such as overfishing, pollution, habitat destruction and climate change [1,2]. Indeed, anthropogenic activities have been changing the oceans for millennia, with fishing being the most ancient activity ~~impact~~ performed by ~~the~~ humans ~~to marine ecosystems~~ [3]. ~~For a long period of time~~ Some types of ~~Over~~fishing hav~~es~~e been unsustainable [4], surpassing the recovery limits of several fish stocks across the globe [5]. The status assessment of fishery resources revealed that the fraction of fish stocks that are within biologically sustainable levels decreased from 90% ~~percent~~, in 1974, to 65.8% ~~percent~~ in 2017 [6]. In contrast, ~~R~~recent studies argue that some fishing stocks are gradually recovering in different parts of the world [7], ~~mainly~~ especially in regions where there are effective fisheries management [8]. Although these recent findings show positive global scenarios [9], overfishing is responsible for substantially modifying the abundance and biomass patterns of species and ~~can~~ decreasing ~~individuals'~~ the body size of individuals in a population [10–13]. Some ~~types of~~ fishing gears ~~fisheries~~ such as longline ~~fishing~~ are usually directed towards top predators~~Fisheries are directed towards top predators~~ (e.g. tunas and sharks) and select individuals with larger body sizes

[14], a common pattern with known consequences for marine ecosystem functioning, including ~~among the consequences is~~ the prey release effect [15,16].

Fishing effort concentrated on large-bodied fish can cause the decline of top predators stocks such as groupers, sharks and tunas [17–21], and even drive species to local extinction [3,22]. These large-bodied species tend to occupy higher levels in the food chain and play a key role in maintaining the trophic structure and functioning of communities [23,24]. Thus, the removal of top predators can trigger cascading effects on lower levels of the food web [24–26]. Declining trends of higher trophic level stocks lead to a systematic replacement by lower trophic level and smaller-bodied target species, a process identified as “fishing down the food web” [27–29]. This process has been measured through a decline in fisheries’ mean trophic level (MTL) [30–33]. In an attempt to understand how fishing impacts marine food~~trophic~~ webs, studies highlight that there are other ways in which fishing may be affecting the ecosystem. In “fishing through the food web” ~~process~~ a decline in MTL is caused due to the sequential addition of low trophic level rather than to a decline of high trophic level catches [25]. The “fishing up the food web” process corresponds to a shift from low to high trophic level species over time, causing an increase in the MTL of catches [34,35]. Multiple hypotheses may explain how fishing affects marine food webs [36], such as profit-driven fishing [37]. ~~-~~The use of catch MTL as an indicator to measure the magnitude of fishing impacts on marine ecosystems may not be reliable in some scenarios, ~~. The authors suggest that~~and ~~the use of this biological indicator~~ should ~~, that it needs to~~ be interpreted cautiously [38]. ~~One way to get a clear picture of what is happening to the ecosystem is to~~The use MTL in combination with other measures may offer a better picture of the ecosystem [39] .

The assessment of temporal changes in MTL and replacement of target species requires long-term fisheries monitoring. However, most low and lower-middle income

Formatted: Not Highlight

countries lack fisheries monitoring programs [40]. In Brazil, such programs are rare and fragmented, and since 2008, there is no governmental fisheries monitoring program established at the national level. In addition, lack of financial resources, a large (~8000 km) and heterogeneous coast, a multigear fishing scenario, differential efforts and target species, pose a challenge to the implementation of fisheries management strategies in Brazil [41,42]. To circumvent the difficulties caused by the lack of fishing data, non-conventional approaches can be useful to estimate the impacts of fishing over time [43–45]. For instance, local ecological knowledge (LEK) may provide a relevant source of historical information, in which marine resource users (e.g. fishers) experienced different scenarios in the past [44,46,47].

A robust assessment of the magnitude and effect of anthropogenic impacts (e.g. fishing) on ecosystems requires knowledge of the unexplored state of these environments [48]. However, throughout human generations, information on composition, size and abundance of species caught on fisheries may be lost, compromising the environmental perception adopted by human populations [49]. Such cross-generational change in baselines has implications to environmental issues, as it results in increased tolerance for progressive environmental degradation because the younger generations consider a more degraded environment as the norm [50,51]. Pauly et al. [50] described this phenomenon as the “shifting baseline syndrome”, a socio-psychological tendency to assume that "natural" conditions of an ecosystem are those first observed by users, researchers and~~/or~~ contemporary managers. When data on the conservation status of the environment is scarce, historical data from alternative sources (e.g. works of art, reports of naturalists, logbooks, photographs, grey literature, and anecdotal information) have been used to help adjust references [52].

Fisher’s LEK has been widely used to investigate the past of fisheries, mainly in data poor countries~~countries that lack long-term monitoring~~. The empirical knowledge of natural

resource users is a cost-effective alternative approach that offers relevant data to inform management. For instance, fishers LEK has~~ve~~ provided information on temporal and spatial patterns of catches [53–55], behavior of targeted species [42,56–59], changes in abundance and body size of targeted species [20,60], trophic and ecosystem models [61,62], and conflicts with other resource users and management interventions [63,64]. In certain cases, LEK has been also associated with other types of data, such as landing data and underwater visual census, to reveal temporal trends in fish populations [54,61,65–67]. These combined approaches offer an opportunity to understand the effect of small-scale fisheries in data poor scenarios – when temporal and systematically collected data are missing. Combining anecdotal evidence from fisher's LEK and fisheries landing data, ~~our study~~we aim~~s~~ to identify temporal changes in (i) small-scale fisher's perception regarding overexploited and new target fish species; (ii) and ~~catches~~landing~~-~~s of overexploited and new target fish species. Specifically, we combined~~using~~ the MTL ~~approach~~metric~~,~~ with fisher~~'~~s' perceptions to reveal temporal changes in local ~~we evaluated changes in local~~ trophic structure caused by fishing. We also discuss the main causes of fish overexploitation and management strategies in a subtropical fisheries hotspot.

Materials and methods

Study site

The study was conducted in Arraial do Cabo, southeastern Brazil (Fig 1). Arraial do Cabo is an ~~important and~~ ancient Brazilian fishing area, where a variety of artisanal fishing gear is employed such as hook and line, gillnet, spearfishing and beach seine. In Arraial do Cabo, artisanal fisheries still occur with small boats such as canoes some without any motors. ~~,~~ Fishers~~which~~ make trips near the shore, or using hook and line or small nets like cast nets in

Formatted: Font: (Default) Times New Roman, 12 pt

the rocky shore or on the beach [68]. The beach seine is ~~a~~ an old/secular practice that was central to the establishment of the local partially protected marine protected area (MPA) – the Arraial do Cabo Marine Extractive Reserve. In this MPA, only local fishers are allowed to fish, with 1600 families relying on fishing activities as the main source of income [68]. Arraial do Cabo holds a diverse marine community, gathering tropical and warm temperate components, which is favored by the coastal morphology and local upwelling events [69,70].

Fig 1. Map of Arraial do Cabo region (state of Rio de Janeiro, Brazil), showing interview sites. Map reprinted from [54] under a CC BY license, with permission from Bender et al (2014), original copyright 2014.

Data collection

Fisher's local ecological knowledge

Between July and August 2018 and 2019, we interviewed 155 artisanal fishers on the fishing communities of Figueira, Monte Alto, Praia Grande, Praia dos Anjos, Prainha, and Pontal (Fig 1). Interviewed fishers correspond to 10.3% of local artisanal fishers (n = 1500) [71]. Interviews were conducted following previous consent and individually, so that there was no influence from others on fishers' answers. Semi-structured questionnaires included questions on (i) which fish species were previously discarded but are now fishing targets; (ii) which species are overexploited in the region; and (iii) what are the main causes of species' overexploitation (S1 File). The fishers answered spontaneously which species they consider overexploited and new targets of fisheries. Fishers were randomly approached at ~~Most of the interviews were carried out at the~~ Praia dos Anjos pier, and ~~where fishers were randomly~~

Formatted: Font: (Default) Times New Roman, 12 pt

invited to participate in the interviews. This procedure was conducted until reaching an appropriate number of interviews (n). ~~In addition, a more experienced fisher helps us with the indication of the interviewees, and to complement the sample number, the fishers were asked to indicate the next interviewee through snowball sampling~~ When completing the questionnaire (S1 File), fishers were asked to indicate an~~the next~~other interviewee [72] ~~through snowball sampling~~.

We also asked fishers their age and years of fishing experience. For data analysis, we chose to use the fisher's experience (time period) rather than age, as these two variables are correlated (~~$P < 0.001$,~~ $r^2 = 0.53$, cor = 0.74, $P < 0.001$, ~~-~~Pearson's correlation test). Fishers were categorized according to their fishing experience into four groups: ~~beginner~~ less experienced (<20 years, n = 24), intermediate (21-35 years, n = 55), experienced (36-40 years, n = 44) and very experienced (>40 years, n = 32). Under this classification, we consider only their time of experience (years of practice), with no qualitative judgment of the real experience of each fisher [73]. We assume that within a given category of experience, all fishers spend the same time fishing, and all gain experience at the same rate [73]. Still, this set of different categories can help estimate changes in fish composition as well as differences in perceptions of trends across generations of fishers. In addition, we chose these experience categories so that they would accommodate similar sample sizes. The small sample size of the less experienced~~beginners~~ category compared to the very experienced is related to the lack of engagement with fishing by the younger generations. Younger fishers are switching to alternative sources of income such as tourism, often encouraged by their families to leave the fishing [71].~~.~~ The interviews were approved by the Ethics Committee of Universidade Federal de Santa Maria, Brazil (CAAAE 29157919.6.0000.5346) and by Sistema de Autorização e Informação em Biodiversidade (Sisbio-ICMBio/IBAMA/Brazil nr. 55911-6).

Fisheries landing data

We searched for landing data on the main species reported by the interviewees. Landing data (e.g. ~~catch~~ in kg) has been incorporated into the study to complement the trends identified by local fishers. Such data corresponds to local annual ~~catches~~ landings ~~(kg)~~ of artisanal fisheries registered between 1992 and 2008 in the port of Arraial do Cabo (Fisheries landing monitoring report, FIPAC).

Mean trophic level

We compiled the trophic level (TL) of 81 species landed in Arraial do Cabo between 1992 and 2008 (S1 Table). The trophic levels were obtained from FishBase, which provides TL estimates from food items for many fish species [74]. For the TL of invertebrates, we used the equivalent database, SeaLifeBase [75].

To assess MTL trends ~~in catches~~ landings over time, we compared the MTL of species in five~~four~~ groups: all landed species, species with TL > 4, TL ≥ 3.5, and TL < 3.5. These categories allow us to assess changes in MTL taking into account the temporal dynamics of higher and lower trophic level species, and visualize potential masking effects [31]. MTL was also calculated excluding low TL species (TL < 3.5), because of possible fisheries expansion in direction to small pelagic species, which can represent an alternative scenario such as 'fishing through' food webs rather than 'fishing down' [25]. High trophic level species (TL > 4) are more sensitive to overfishing, as they have life history traits ~~characteristics~~ such as late maturation, low fertility rates, and slow growth [76]. ~~ThereforeIn this way, the unsustainable removals of their populations se species can bring potential risks to the conservation of their populations.~~ Therefore, we examined the dynamics of species' stocks with a threshold value of TL > 4, as done previously ~~previously analyzed in another study~~

[25]~~Top predators are more sensitive to overfishing, thus the dynamic of species stocks with a threshold value of TL > 4 was examined in previous studies~~ . Species with TL > 3.5 (e.g. tunas, cod and groupers) are considered as mesopredators that feed on a mixture of low and high-TL organisms [77]. Also, s~~S~~pecies with TL < 3.5 were assessed ~~in order~~ to observe changes in the contribution of low trophic level species [77]. We used these threshold values to eliminate the masking effect and to emphasize changes in the relative abundance of fish from certain trophic groups. The MTL was estimated as follows [27]:

$$MTL = \frac{\sum TL_{ij} Y_{ij}}{\sum Y_{ij}}$$

W~~w~~here MTL is the mean trophic level of landing in year *j*, *Yij* the landing of species *i* in year *j* and *TLi* is the trophic level of species *i*.

We used a null model approach to test whether citations of overexploited and new target species (by fishers), within a given trophic level, are more frequent than expected at random. We generated 1000 random samples for the number of citations of each species within the overexploited and new target species groups. For each random sample, we calculated the MTL weighted by the citation number, using the following equation:

$$MTLc = \frac{\sum TLi\ Ci}{\sum Ci}$$

~~w~~Where MTL_c is the mean trophic level of overexploited and new target species and *Ci* is the total number of citations of species *i*.

We then plotted the confidence intervals (C.I. 95%) of the null MTL obtained for overexploited and new target species and the observed MTL_c, to verify if the citation frequency differs than expected at random.

Data analysis

To estimate changes in fishers' perception of target species, we applied a Generalized Linear Model (GLM) between the (i) number of overexploited species and (ii) number of new target species vs. experience of fishers (years of practice). The number of cited species was set as the response variable. Because numbers of species are count~~able~~ data, we assumed a Poisson distribution. The experience represents the explanatory variable. Under these specifications, the equation of model was:

$$log(\mu i) = \beta 0 + \beta 1\ xi$$

Where μi is the conditional expectation of *yi*, E(y|x), $\beta 0$ is the coefficient marked (Intercept) and $\beta 1$ the coefficient marked *x*. We assumed the model of the mean number of species μi using a log link.

We measured the mean percent of increase in citations (*P*) of overexploited fish species and new target species according to fishers' experience (*F*) using the GLM model coefficients (*coef1* and *coef2*):

$$P = \left(\frac{e^{(coef1+coef2*F)}}{e^{(coef1+coef2*0)}} - 1\right) * 100$$

To test for differences between the proportion of overexploited and new target species (previously discarded) mentioned by fishers in different experience categories, we applied a Z-test. We then verified differences between groups through a pairwise comparison of proportions. Regression analyse~~i~~s were used to measure temporal ~~catch~~ trends in landings of fish species by plotting the landed weight (in tons) over the years. All analyzes were performed on R software v. 3.6.1 [78].

Results

Fishers with more years of practice recognized a significantly greater number of overexploited fish species than those with fewer years of practice (β = 0.009, df = 153, *P* = 0.008) (Fig 2a, S2 Table). This relationship ~~can also be observed~~is clearly detected by an increase in the mean percent in the citation, ~~which~~ relateds with years of practice (Fig 2b). We observed the same pattern for the number of species recognized as new target species. As time of practice increases, fishers mentioned a significantly greater number of species as new targets of local fisheries (β = 0.010, df = 153, *P* = 0.01) (Fig 2c, S2 Table). In addition, there is an increase in the percent of citations of new target species by fishers' with more years of practice (Fig 2d).

Fig 2. (a) Number of species mentioned as overexploited according to fisher's experience (years of practice); (b) Probability of citations of overexploited species according to fisher's experience; (c) Number of species mentioned as new target species according to fisher's experience; and (d) Probability of citations of new target species according to fisher's experience. Experience categories: LESS_EXP = less experienced; INTER = intermediate; EXP = experienced; and VER_EXP = very experienced.

In total, 37 species were identified as overexploited by local fishers (Table 1). The bluefish (*Pomatomus saltatrix*) was the most frequently cited species, across all experience categories. The proportion of citations of *P. saltatrix* were similar between categories, with at least 45% of fishers in different experience categories recognizing bluefish as overexploited: 56% of very experienced (n = 18), 55% of experienced (n = 21), 47% of intermediate (n = 18), and 45% of ~~beginner~~ less experienced fishers (n = 21) (Fig 3a). The second most cited species was the largehead hairtail (*Trichiurus lepturus*), although there are no significant differences between the experience categories, the species was ~~more~~ mentioned more by

intermediates (26%, n = 10) and less experiencedbeginners (30%, n = 14) fishers. The dusky grouper (*Epinephelus marginatus*) and the crevalle jack (*Caranx hippos*) were most frequently cited by very experienced fishers (> 40 years of practice) and had a significant difference in the proportion of citations between very experienced and beginners (P = 0.03 and P = 0.001, respectively). In addition to *C. hippos*, the lesser amberjack (*Seriola fasciata*) was another carangid referred to as overexploited (Fig 3a).

Table 1. Species reported as overexploited by fishers in descending order of number of citations (N). Percentage of citation (%) of species by fishers (n = 155). Trophic levels were obtained from FishBase database where a higher value means higher trophic levels (Froese and Pauly, 2019). Trophic categories were obtained from the Southwestern Atlantic reef fish database (Pinheiro et al., 2018). The conservation status at global level derives from the IUCN Red List of Threatened Species (IUCN, 2019) and at national level from the Brazilian Red List of Threatened Species (MMA, 2018).

**OVEREXPLOITED SPECIES**							
**Order**	**Species**	**N**	**%**	**Trophic level**	**Trophic category**	**Global status**	**Regional status**
1	*Pomatomus saltratrix*	78	50.3	4.5	MCAR	VU	NT
2	*Trichiurus lepturus*	37	23.8	4.4	MCAR	LC	LC
3	*Epinephelus marginatus*	23	14.8	4.4	MCAR	VU	VU
4	*Seriola fasciata*	22	14.1	4.5	MCAR	LC	DD
5	*Caranx hippos*	20	12.9	3.6	MCAR	LC	LC
6	*Pseudopercis numida*	20	12.9	3.9	MCAR	LC	NT

7	*Hyporthodus niveatus*	16	10.3	4	MCAR	VU	VU
8	*Seriola lalandi*	14	9	4.2	MCAR	LC	LC
9	*Mugil liza*	11	7	2	HERB	DD	NT
10	*Scomberomorus cavalla*	11	7	4.4	MCAR	LC	LC
11	*Sardinella brasiliensis*	10	6.4	3.1	PLANK	DD	DD
12	*Conger orbignyanus*	10	6.4	3.7	MCAR	LC	DD
13	*Sarda sarda*	8	5	4.5	MCAR	LC	LC
14	*Cynoscion striatus*	7	5	3.9	MCAR	NE	NE
15	*Mycteroperca microlepis*	6	3.8	3.7	MCAR	VU	DD
16	*Coryphaena hippurus*	6	3.8	4.4	MCAR	LC	LC
17	*Seriola dumerili*	6	3.8	4.5	MCAR	LC	LC
18	*Priacanthus arenatus*	5	3.2	4	MINV	LC	LC
19	*Katsuwonus pelamis*	5	3.2	4.4	MCAR	LC	LC
20	*Caranx crysos*	4	2.5	4.1	MCAR	LC	LC
21	*Caranx latus*	4	2.5	4.2	MCAR	LC	LC
22	*Pagrus pagrus*	3	1.9	3.9	MINV	LC	DD
23	*Trachinotus carolinus*	3	1.9	3.5	MINV	LC	LC
24	*Micropongonias furnieri*	3	1.9	3.1	MINV	LC	LC
25	*Cynoscion acoupa*	3	1.9	4.1	MCAR	LC	NT
26	*Lopholatilus villarii*	2	1.2	3.8	MCAR	NE	VU

27	*Balistes vetula*	2	1.2	3.8	MINV	NT	NT
28	*Diplodus argenteus*	2	1.2	3.1	OMNI	LC	LC
29	*Peprilus paru*	2	1.2	4.5	MCAR	LC	LC
30	*Centropomus undecimalis*	2	1.2	4.2	MCAR	LC	LC
31	*Scomber colias*	1	0.6	3.4	MCAR	LC	LC
32	*Scomberomorus brasiliensis*	1	0.6	3.3	MCAR	LC	LC
33	*Aluterus monoceros*	1	0.6	3.8	OMNI	LC	NT
34	*Lagocephalus laevigatus*	1	0.6	4	MCAR	LC	LC
35	*Carcharhinus leucas*	1	0.6	4.3	MCAR	NT	NT
36	*Isurus oxyrinchus*	1	0.6	4.5	MCAR	NT	NT
37	*Pseudobatos horkelii*	1	0.6	3.8	MINV	CR	CR

Trophic categories: Macrocarnivore (MCAR); Mobile Invertivore (MINV); Omnivore (OMNI); Planktivore (PLANK); Herbivore (HERB). IUCN categories: Critically endangered (CR); Vulnerable (VU); Near threatened (NT); Least Concern (LC); Data deficient (DD); Not evaluated (NE).

Fig 3. Fish species most frequently cited as overexploited (a) and new target species (b) according to fisher's experience (years of practice). Letters indicate significance ($P < 0.05$) between categories of experience of each species.

Fishers identified 36 new target species that were previously discarded by local fisheries (Table 2). *T. lepturus* was cited by 63% of very experienced fishers, 76% of experienced, 71% of intermediates, and 66% of less experienced fishers ~~beginners~~ (Fig 3b). The second most cited species was the grey triggerfish (*Balistes capriscus*), followed by the Argentine conger (*Conger orbignyanus*), the unicorn leatherjacket filefish (*Aluterus*

monoceros) and the Atlantic bigeye (*Priacanthus arenatus*) (Fig 3b). For *T. lepturus* and *C. orbignyanus* the proportion of citations was similar across experience categories (Fig 3b). The citations for *P. arenatus* decreased with fisher's experience, therefore being greater for less experienced~~beginner~~ fishers. Only the citations of *B. capriscus* had significant differences between experience categories, being proportionally higher for experienced compared to intermediate fishers (P = 0.01) (Fig 3b).

Table 2. Species cited as new targets by fishers, in descending order of reports (N). Percentage of citation (%) of species by fishers (n = 155). Trophic levels were obtained from FishBase where a higher value means higher trophic levels (Froese and Pauly, 2019). Trophic categories were obtained from the Southwestern Atlantic reef fish database (Pinheiro et al., 2018). The conservation status at global level derives from the IUCN Red List of Threatened Species (IUCN, 2019) and at national status from the Brazilian Red List of Threatened Species (MMA, 2018).

NEW TARGET SPECIES							
**Order**	**Species**	**N**	**%**	**Trophic level**	**Trophic category**	**Global status**	**Regional status**
1	*Trichiurus lepturus*	107	69	4.4	MCAR	LC	LC
2	*Balistes capriscus*	27	17.4	4.1	MINV	VU	NT
3	*Conger orbignyanus*	26	16.7	3.7	MCAR	LC	DD
4	*Aluterus monoceros*	24	15.4	3.8	OMNI	LC	NT
5	*Priacanthus arenatus*	23	14.8	4	MINV	LC	LC
6	*Scomber colias*	13	8.3	3.4	MCAR	LC	LC
7	*Sardinella brasiliensis*	12	7.7	3.1	PLANK	DD	DD
8	*Katsuwonus pelamis*	11	7	4.4	MCAR	LC	LC
9	*Lagocephalus laevigatus*	9	5.8	4	MCAR	LC	LC
10	*Diplodus argenteus*	8	5.1	3.1	OMNI	LC	LC
11	*Porichthys porosissimus*	5	3.2	3.7	MINV	NE	LC

12	*Mugil liza*	4	2.5	2	HERB	DD	NT
13	*Pomatomus saltatrix*	4	2.5	4.5	MCAR	VU	NT
14	*Micropogonias furnieri*	4	2.5	3.1	MINV	LC	LC
15	*Cynoscion striatus*	3	1.9	3.9	MCAR	NE	NE
16	*Umbrina canosai*	3	1.9	3.9	MINV	NE	LC
17	*Selene setapinnis*	3	1.9	3.7	MCAR	LC	LC
18	*Dactylopterus volitans*	3	1.9	3.7	MINV	LC	LC
19	*Trachinotus carolinus*	2	1.2	3.5	MINV	LC	LC
20	*Caranx latus*	2	1.2	4.2	MCAR	LC	LC
21	*Cynoscion acoupa*	2	1.2	4.1	MCAR	LC	NT
22	*Chloroscombrus chrysurus*	2	1.2	3.5	PLANK	LC	LC
23	*Seriola fasciata*	2	1.2	4.5	MCAR	LC	DD
24	*Pseudopercis numida*	2	1.2	3.9	MCAR	LC	NT
25	*Carcharhinus plumbeus*	1	0.6	4.5	MCAR	VU	CR
26	*Isurus oxyrinchus*	1	0.6	4.5	MCAR	NT	NT
27	*Squatina argentina*	1	0.6	4.1	MCAR	EN	CR
28	*Sparisoma axillare*	1	0.6	2	HERB	DD	VU
29	*Epinephelus marginatus*	1	0.6	4.4	MCAR	VU	VU
30	*Carcharhinus brevipinna*	1	0.6	4.2	MCAR	VU	DD
31	*Cynoscion jamaicensis*	1	0.6	3.8	MCAR	LC	LC
32	*Pseudobatos horkelii*	1	0.6	3.8	MINV	CR	CR
33	*Lopholatilus villarii*	1	0.6	3.8	MCAR	NE	VU
34	*Cynoscion guatucupa*	1	0.6	3.7	MCAR	NE	LC
35	*Pagrus pagrus*	1	0.6	3.9	MINV	LC	DD
36	*Sarda sarda*	1	0.6	4.5	MCAR	LC	LC

Trophic categories: Macrocarnivore (MCAR); Mobile Invertivore (MINV); Omnivore (OMNI); Planktivore (PLANK); Herbivore (HERB). IUCN categories: Critically endangered (CR); Vulnerable (VU); Near threatened (NT); Least concern (LC); Data deficient (DD); Not evaluated (NE).

The same declining trend reported by fishers for *P. saltatrix*, *T. lepturus*, *E. marginatus*, *C. hippos*, and *S. fasciata* (Fig 3a) were revealed by fisheries landing data (Fig

4). Since the 1990s, *P. saltatrix* stocks declined significantly (i.e. approximately 47%) ~~(*P* < 0.001)~~ (Fig 4a), this status being perceived by fishers across all experience categories. For *T. lepturus*, the ~~catches~~ landings declined significantly after the 2000s ~~(*P* < 0.001)~~ (Fig 4b), ~~which~~ pattern promptly ~~has been~~ identified by the majority of fishers in less experienced categories. This species has been considered overexploited and also identified as a new target species in the region. Landing data show that since the 1990s, landings~~catches of~~ *E. marginatus* declined 61%, *C. hippos* ~~catches~~ landings diminished by 88%, and *S. fasciata* by 89%, the latter showed significant declining trends ~~(*P* < 0.001)~~ (Fig 4c, e, g). This overexploitation scenario was observed by very experienced fishers, which cited the first two species as overexploited in a higher proportion (Fig 3a).

Fig 4. Artisanal fisheries (tons) landings in Arraial do Cabo from 1992 to 2008. Species on the left (a, c, e, g) correspond to those most frequently cited as overexploited in interviews and on the right (d, f, h), new target species reported by local fishers. (b) *Trichiurus lepturus* corresponds to both an overexploited and a new target species. a, b, d, f, g and h, are second-order polynomial regressions; c and e, an exponential regression.

The ~~catches~~ landings data of the new target species exhibited an increase from the 2000s onwards, followed by a reduction in the ~~amount landed catch~~quantities of landed fish over the years (Fig 4b, d, f, h). From the 1990s to 2000s, the landings of new target species as *T. lepturus, C. orbignyanus*, and *A. monoceros* had an increase ~~in landed catches~~ (Fig 4b, d, h). By the mid-2000s, ~~catches~~ landings of these species declined 69%, 55% and 85%, respectively (Fig 4b, d, h). Fishers also reported this trend, when respondents mentioned these species as overexploited (Fig 3b; Table 1). Landings of *P. arenatus* had a significant increase from 2000 onwards ~~(*P* < 0.001)~~ (Fig 4f), mainly reported by less experienced fishers (Fig 3b).

Catch MTL of TL ≥ 3.5 and TL > 4 species declined significantly from 1992 to 2008 (r^2 = 0.26; P = 0.03 and r^2 = 0.43; P < 0.001, respectively) (Fig~~ure~~ 5a, b). When MTL of all landed species are represented, there were~~as~~ ~~a~~ non-significant trends in ~~increase in~~ MTL over time (r^2 = 0.06; P = 0.32). ~~–~~For~~The increasing trend was observed with~~ species with TL < 3.5, the increasing trend was non-significant (r^2 = 0.07; P = 0.14) (Fig 5a, b).

In the years of 1995 and 1999, there were peaks in landings of all species in Arraial do Cabo (Fig 5c). However, between 2001 and 2004, there were sharp declines in landings for all groups ~~(TL > 4, TL ≥ 3.5, all species, and TL < 3.5)~~ (Fig ~~5c~~5b). Declining trends were also identified for species with TL > 4 through landing data. For the top 10 species ~~landed species~~, we observed a decrease in landings between 1992 and 2008 (Fig 5d).

Fig 5. Trends in MTL of species landed in Arraial do Cabo from 1992 to 2018. (a) Linear regression of catch MTL; (b) ~~moving average of MTL catch; and (c)~~ Landing ~~catch~~ data in tons; (c~~b~~) T~~t~~hird - order polynomial regression of landings (in tons) of species with TL > 4; and (d) Landings of the top 10 ~~main~~ landed species ~~landed~~ with TL> 4. The colors represent MTL calculated after excluding all species below trophic level 4 (blue), calculated after excluding all species below trophic level 3.5 (yellow), ~~calculated with all species landed, excluding sardines (*Sardinella brasiliensis*) (yellow),~~ calculated with all species landed (red), and for ~~only~~ species with trophic level below 3.5 (green).

When contrasting the MTL of species mentioned by fishers as overexploited and as new target species, we observed that the citations of overexploited species ~~are~~ were more frequently within higher TL species relative to new target ones (S1 Fig). In addition, the MTL observed for overexploited species was higher than expected by chance (S1 Fig).

Fishers mentioned 35 different ~~possible~~ causes of fisheries overexploitation in Arraial do Cabo (1.58 ± 1.08, mean ± standard deviation). The five most mentioned causes were the increased fishing effort (22%), which ~~is~~ was recognized by fishers as an increase in the number of fishers and boats; ~~the~~ presence of industrial fishing vessels (21%), ~~the~~ presence of purse seine fisheries (19%), gillnet fishing (16%), and trawling (12%).

Discussion

Our study revealed a replacement of target species identified by fisher's knowledge and landing data in a Brazilian secular fishing village~~the Arraial do Cabo~~. Here, we showed an overexploitation scenario of large and valuable fish species and their replacement by new target species. The decline of species stocks such as *P. saltatrix, E. marginatus, C. hippos* and *S. fasciata* were followed by sequential substitutions by less commercially valuable but more abundant species (e.g., *T. lepturus, B. capriscus, A. monocerus* and *P. arenatus*). Temporal changes in the landing composition of fish species have been reported around the world [29,79–81]. In general, changes are from large-bodied species belonging to high trophic levels to smaller and lower trophic level species [10], and/or replacement of fish to invertebrate species [29]. In southern Brazil, a study used fishing landings, market values and multispecies indicators including MTL to evaluate changes and fishing impacts over time [82]. As in our study, authors did not identify declines in MTL of landings. However, they report the decline of most high trophic level species, as sharks, indicated from market data and landing volumes. This study also suggests that changes based on a single indicator such as MTL can be masked, and the use of multiple approaches can make these changes more explicit [82]. In this context, our study is the first to indicate temporal changes in the composition of fishing resources by combining fisher's knowledge and landing data in Southeastern Brazilian Coast.

Our results show that fisher's perception of overexploited and new target species is shaped by their fishing experience. As experience increases with years of fishing practice, fishers are able to recognize a greater number of overexploited and new target species. These findings indicate the occurrence of a shifted baseline among fishers' generations, which in this case, is the loss in fisher perception regarding changes in fisheries' composition and the decline of fish species throughout generations [50]. At the time young and less experienced fishers started fishing, several species were already overexploited, compromising their current perception of fish stocks. The small number of species reported by less experienced fishers compared with experienced ones may reflect the lack of familiarity and/or a reduced interaction of fishers with the natural environment [49]. Such disconnection with past conditions has effects on the willingness and motivation of new generations to accept management restrictions aimed to restore resources and even mislead recovery perceptions, the "placebo effect" [83]. In Arraial do Cabo, the overexploitation of fishing resources [21,54] has made less experienced fishers look for alternative income sources. Several less experienced ~~young~~ fishers have mentioned being involved in ~~any type of~~ tourism related activities, using their boats for sightseeing, as a parallel activity to fishing. This reduces the engagement of young people with fishing and possibly impairs their perception of the status of fishing resources. The loss of familiarity with the natural environment of future generations can result in the absence of past information or experience with historical conditions [49], and members of each new generation accept the ~~situation~~scenario in which they were raised as being normal.

Our results revealed declining trends in ~~catches~~ landings of commercially important ~~the macrocarnivores~~ mesopredators ~~*P. saltatrix, E. marginatus, C. hippos,* and *S. fasciata*~~ in Arraial do Cabo. Declines of ~~macrocarnivores~~high trophic species like groupers and bluefish have been reported over the last decades [54] ~~A previous survey showed a decline of 80% in *P. saltatrix* catches over the past six decades, and reductions in the abundance and body size of *E. marginatus* for the same period~~. *P. saltatrix* and *E. marginatus*~~These two species~~, one

being pelagic and one a reef-associated, are important resources in Arraial do Cabo, as well as along the south and southeastern Brazilian coast [84]. Our findings highlight that very experienced fishers have identified the overexploited status of *E. marginatus* and *C. hippos* populations in a greater proportion. ~~Previous studies show that m~~More experienced fishers have reported higher catches of ~~groupers~~ target species than less experienced ones [17,20,53,60,73,85], this being a common scenario of overexploitation in small-scale fisheries since fish resources are decreasing. For instance, decades ago~~. For a long time~~, groupers were abundant in Arraial do Cabo, and generations of more experienced fishers witnessed large catches of the species in the past. Today, older fishers perceive this decline, and catches of groupers have become rare events in the region. Due to life history traits such as high longevity, slow growth, late maturation, and low reproductive rates, groupers are highly sensitive to fishing effects [86,87]. Such characteristics have contributed to the threatened status of many grouper species worldwide [15,88]. The decline of other predator species as *C. hippos* and *S. fasciata* is concerning, since these species are important regional fishing resources [89], and as expected for large carnivores, depict a combination of biological traits that can enhance their extinction risk [90].

Large catches of *T. lepturus* in Arraial do Cabo occurred incidentally in the past, i.e. when fishers targeted other species with high commercial value such as *P. saltatrix* and *E. marginatus*. The largehead hairtail was identified both as overexploited and new target species, mostly by less experienced~~younger~~ fishers. Fishing of this species is more recent than of other fishes mentioned as overexploited. Thus, knowledge about the exploitation status of this species is more widespread among young generations. Due to its low commercial value and lack of consumer market, large quantities of *T. lepturus* were previously discarded. Later on, the decline of large predators in the region [21,54], led fishers to replace their target species. This has made *T. lepturus* economically valuable and a target of local fisheries,

which was also observed for *B. capriscus, C. orbignyanus* and *A. monoceros*. Fishing directed at other less valuable species has become an alternative to maintain local fishing activities. The collapse of higher trophic level species caused by overfishing led fishers to target less valuable and lower trophic level species, similar trends are found in case studies that report the occurrence ~~a case~~ of fishing down marine food webs [29].

The fishing down the food web process predicts continuous declines in catches of high trophic level and a shift to small, low-trophic level species~~increases in catches of lower trophic level species~~ [27]. This ubiquitous phenomenon has been identified in several ecosystems, both at local and global scales [30,39,67,91,92, see more case studies in www.fishingdown.org]. Here, we demonstrate the ~~occurrence of this phenomenon~~scenario of overexploitation of high trophic species caught in Arraial do Cabo [54], combining alternative data sources from fishers' LEK and ~~,~~ landing data (from 1992 to 2008), supported by other local studies [54]. ~~The MTL index (TL ≥ 3.5 and TL> 4) [25]. Fishing is an old ancient activity in this region,~~ We observed a declining trend~~s~~ in the mean trophic level and landings of species with TL > 4 and TL ≥ 3.5. When considering the MTL of all species landed, there is no clear pattern in trends, probably due to the decrease in landings of species of low trophic level, such as sardines, and the significant declines in landings of large predators. The same case has been identified in studies in upwelling systems in the Mediterranean Sea [93,94]. Studies point out that in some cases fishing down may not be identified [39], due to s~~S~~everal factors that are responsible for masking the prevalence of this~~e~~ ~~fishing down~~ process [39,95], including taxonomic over-aggregation, the body size of species and geographical expansion of fisheries [33]. ~~landings of top predators, as previously reported by fishers, are one of the consequences of the overfishing scenario in this region. It is therefore essential to understand how fishing affects food webs for efficient fisheries management in Arraial do Cabo. identified for fisheries in the Mediterranean Sea and in some upwelling systems [82, 83].~~

Catch Landing MTL assessments considering all landed species can induce a "masking effect" [84, 81]. Several factors are responsible for masking the prevalence of the fishing down process [85, 86], including taxonomic over-aggregation, the body size of species and geographical expansion of fisheries [31]. In contrast, disregarding expansion leads to a misinterpretation of the increase in MTL, as reported in previous studies [87]. Ultimately, studies encourage the use of Thus, studies suggest the use theof MTL is suggested as a complementary tool for the assessment of the effects of fishing on ecosystems [39,96]. Here, we combine multiple sources of information (local ecological knowledge, landing data, and MTL as a complementary metric) to investigate temporal changes in marine resources caught by artisanal fishing in Arraial do Cabo.

Formatted: English (United States)

Formatted: English (United States)

Formatted: Font: (Default) Times New Roman, 12 pt

Fishers LEK revealed that, in the past, large catches of tunas, sharks and groupers were common in Arraial do Cabo (Fig 6). Shark species such as the spinner shark (*Carcharhinus brevipinna*) were caught in large quantities (Fig 6b), as well as the and currently threatened species the Brazilian guitarfish (*Pseudobatos horkelii*) and the sandbar shark (*Carcharhinus plumbeus*) [54]. Despite never being a major fishing target, these shark species are still caught in the region, but in smaller quantities. The current fishing scenario revealed in the present study is quite different from that reported by the old fishers. Despite its low trophic level, the large body size of parrotfishes such as the endemic greenbeak parrotfish (*Scarus trispinosus*) has made this species a major target of artisanal fisheriesspearfishing in the Southwestern Atlantic [54 97, 9[illegible]]. In Arraial do Cabo, a past study had identified reductions in the abundance and body size of *S. trispinosus* individuals [54]. In addition, spearfishers commonly catch other large parrotfishes as those of the *Sparisoma* genus, indicating shifts in the composition of target species [54].

Fig 6. Photographs showing past and present catches~~captures~~ of ~~in Arraial do Cabo. S~~species mentioned by fishers as overexploited and new target species in Arraial do Cabo: (a) ~~Catch of the~~ *Epinephelus marginatus* in 1982; (b) *Carcharhinus brevipinna* caught in Praia Grande in 2005; (c) ~~Catch of~~ *Balistes capriscus* and (d) beach seine fishing at Praia Grande recorded in 2020.

As we expected, ~~The main cause more mentioned by fishers as responsible for fisheries~~ increasesing in ~~the~~ fishing effort ~~overexploitation~~ was described by ~~the~~ fishers as the main cause of overexploitation ~~increase in the fishing effort~~. While local reports have revealed a declining trend in the number of boats (~~S2 Table,~~ S2 Fig a, S3 Table). ~~, fishing effort in the number of fishing days has increased over time (S2 Table, S2 Fig. b).~~ Due to the overexploited state of fish stocks, fishers have to spend more time fishing to obtain the same yields as in the past. Another cause of overexploitation reported by fishers was purse seine fisheries. This fishing gear has a high catch potential, and it is less selective when compared to other artisanal fishing gear (e.g. line and hook). For example, in 2019, purse seine catches represented 59% (231~~.16~~ kg) of the total landed catch in the region (S2 Fig). In addition, purse seine effort in fishing days corresponded to only 6% of the total fishing days (S2 Fig). Therefore, despite changes in fishing effort, the scenario of species overexploitation is worrisome and can be even greater than reported in our study.

The few existent ~~Our results provide evidence that~~ management initiatives in the region, which rule over spawning periods of few species, have been ineffective to change the decline trends of fishing resources in Arraial do Cabo. Classical management strategies as minimum landing size of capture, closure periods, fishing quotas with quantities determined after stock assessments should be implemented based on national~~species'~~ conservation status of species. We believe that such strategy may be viable through an adaptive comanagement framework involving stakeholders in all steps of the process. Management may be real-time

informed by the small-scale fisheries ~~management~~monitoring that occur in ~~the MPA~~that marine extractive reserve. Threatened species as *E. marginatus, Hyporthodus niveatus, Lopholatilus villarii,* and *Sparisoma axillare,* which were mentioned by fishers as overexploited and new target species (Table 1 and 2), should not be targeted until stocks are recovered. As an alternative to minimize illegal fishing off the season and the incidental catch of juveniles, resource users should be oriented to target healthy fish stocks or to search for alternative income sources such as community-based ~~eco~~tourism initiatives. A previous study has compared the effects of partially protected areas vs. unprotected areas in the abundance and body size of reef fish, including a site in Arraial do Cabo [99]. Groupers had higher density and relative abundance in a partially protected area, indicating that even small no-take areas can help recover~~function to recovery some~~ fish populations [99]. The case of Arraial do Cabo is not different from many reef sites around the world, where the fishing effort has surpassed the recovery potential of natural resources [100]. The good news is that even at relatively small areas groupers can increase in density and size if fishing ceases [99]. Additional ongoing acoustic telemetry study~~tagging experiments~~ in the region indicate relatively ~~short~~ small home ranges (0.05 km^2) for two vulnerable parrotfishes~~-~~ (~~,~~*Sparisoma frondosum* and *S. axillare*) [101], which have implications for establishing the minimum size of no-takes areas (authors, per. com.). Small ~~MPAs~~ no-~~-~~take areas appear as a critical tool to preserve local resources, but more importantly, to show local users their efficiency in rebuilding stocks. Additionally, local fishers can be empowered to explore future established no-~~-~~take areas through ~~by community-based eco~~tourism initiatives, having exclusivity over local tourism agencies.

We also highlight the need for greater inspection by the authorities to ~~prevent~~ banning illegal fishing by industrial vessels such as trawl and gillnet fisheries that occur even within the MPA. Encourage dialogue between fishers, especially older and younger generations, so

that the elders transmit their knowledge on~~about~~ ~~the species captured in the~~ the past of local fisheries, helping young generations to understand the abundance trends of fish resources. ~~A~~The ~~understand~~knowledge of how marine resources were more abundant in the past is linked with higher willingness to collaborate with management and conservation initiatives. This may also influence the ~~occurrence~~ overcome of ~~Environmental education programs aimed at local fishers could minimize the lack of knowledge on the exploitation state of resources, and the influence of~~ the shifting baseline syndrome among fishers.

Here we demonstrated that experienced fishers better recognize changes in fisheries and in marine ecosystems when compared to less experienced ones. In addition, we identified changes in composition and abundance of pelagic and demersal, reef-associated fish species caught in a secular fishing village using fishers' ~~LEK~~ local ecological knowledge and fisheries landing data. Despite the short time series, fishing monitoring data reveals declining trends in stocks of mesopredators species overtime. The combination of multiple sources of information can help prevent biases as underestimated assessments of the impact of fishing [102]. By incorporating past data, it is possible to identify changes in catches over time, as well as to adjust conservation goals and strategies for marine ecosystems.

Acknowledgments

C.O.F. acknowledges Coordenação de Aperfeiçoamento de Pessoal de Nível Superior (CAPES) for financial support. V.J.G. received a post-doctoral grant #2017/22273-0, São Paulo Research Foundation (FAPESP). CELF is supported by grants from FAPERJ and CNPq. Costão Rochoso Project (FUNBIO Grant Pesquisa Marinha 020/2017) has provided financial, and logistic support during field sampling in Arraial do Cabo. This work is part of a research program supported by Serrapilheira Institute (grant number Serra-1708-15364) awarded to GO ~~uilherme~~

~~Ortigara~~ Longo (PI). In addition, we thank fishers for their collaboration and shared knowledge. ICMBio has ever been supportiveness for our projects.

References

1. Worm B, Barbier EB, Beaumont N, Duffy JE, Folke C, Halpern BS, et al. Impacts of biodiversity loss on ocean ecosystem services. Science. 2006; 314 (5800): 787-790.

2. Harnik PG, Lotze HK, Anderson SC, Finkel ZV, Finnegan S, Lindberg DR et al. Extinctions in ancient and modern seas. Trends Ecol Evol. 2012; 27 (11): 608-617.

3. Jackson JBC, Kirby MX, Berger WH, Bjorndal KA, Botsford LW, Bourque BJ, et al. Historical overfishing and the recent collapse of coastal ecosystems. Science. 2001; 293 (5530): 629-637.

4. Jennings S, Kaiser MJ. The effects of fishing on marine ecosystems. In Advances in Marine Biology. Academic Press. 1998; 34, pp. 201-352.

5. Pauly D, Zeller D. Catch reconstructions reveal that global marine fisheries catches are higher than reported and declining. Nat Commun. 2016; 7, 10244.

6. FAO. The State of World Fisheries and Aquaculture 2020. Sustainability in action. Rome. 2020.

7. Knowlton N. Ocean Optimism: Moving Beyond the Obituaries in Marine Conservation. Annu Rev Mar Sci. 2020; 13.

8. Hilborn R, Amoroso RO, Anderson CM, Baum JK, Branch TA, Costello C, et al. Effective fisheries management instrumental in improving fish stock status. PNAS. 2020; 117 (4): 2218-2224.

9. Duarte CM, Agusti S, Barbier E, Britten GL, Castilla JC, Gattuso JP et al. Rebuilding marine life. Nature. 2020; 580 (7801): 39-51.

10. Stevens JD, Bonfil R, Dulvy NK, Walker PA. The effects of fishing on sharks, rays, and chimaeras (chondrichthyans), and the implications for marine ecosystems. ICES J Mar Sci. 2000; 57 (3): 476-494.

11. Shin YJ, Rochet MJ, Jennings S, Field JG, Gislason H. Using size-based indicators to evaluate the ecosystem effects of fishing. ICES J Mar Sci. 2005; 62 (3): 384-396.

12. Coll M, Carreras M, Ciércoles C, Cornax MJ, Gorelli G, Morote E, et al. Assessing fishing and marine biodiversity changes using fishers' perceptions: the Spanish Mediterranean and Gulf of Cadiz case study. PLoS One. 2014; 9 (1): e85670.

13. Peñaherrera-Palma C, Putten IV, Karpievitch YV, Frusher S, Llerena-Martillo Y, Hearn AR, et al. Evaluating abundance trends of iconic species using local ecological knowledge. Biol Conserv. 2018; 225, 197-207.

14. Shackell NL, Frank KT, Fisher JA, Petrie B, Leggett WC. Decline in top predator body size and changing climate alter trophic structure in an oceanic ecosystem. Proc Royal Soc B. 2010; 277 (1686): 1353-1360.

15. Heithaus MR, Frid A, Wirsing AJ, Worm B. Predicting ecological consequences of marine top predator declines. Trends Ecol Evol. 2008; 23 (4): 202-210.

16. Sandin SA, Walsh SM, Jackson JB. Prey release, trophic cascades, and phase shifts in tropical nearshore ecosystems. Trophic Cascades: predators, prey, and the changing dynamics of nature. 2010; 71-90.

17. McClenachan L. Historical declines in goliath grouper in south Florida. Endang Species Res. 2009; 7 (3): 175-181.

18. Venkatachalam AJ, Price ARG, Chandrasekara S, Sellamuttu SS, Kaler J. Changes in frigate tuna populations on the south coast of Sri Lanka: evidence of the shifting baseline syndrome from analysis of fisher observations. Aquat Conserv Mar Freshwater Ecosyst. 2010; 20 (2): 167-176.

19. Luiz OJ, Edwards AJ. Extinction of a shark population in the Archipelago of Saint Paul's Rocks (equatorial Atlantic) inferred from the historical record. Biol Conserv. 2011; 144 (12): 2873-2881.

20. Giglio VJ, Luiz OJ, Gerhardinger LC. Depletion of marine megafauna and shifting baselines among artisanal fishers in eastern Brazil. Anim Conserv. 2015; 18 (4): 348-358.

21. Giglio VJ, Bender MG, Zapelini C, Ferreira CEL. The end of the line? Rapid depletion of a large-sized grouper through spearfishing in a subtropical marginal reef. Perspect Ecol Conserv. 2017; 15 (2): 115-118.

22. Lewison RL, Crowder LB, Read AJ, Freeman SA. Understanding impacts of fisheries bycatch on marine megafauna. Trends Ecol Evol. 2004; 19 (11): 598-604.

23. Myers RA, Baum JK, Shepherd TD, Powers SP, Peterson CH. Cascading Effects of the Loss of Apex Predatory Sharks from a Coastal Ocean. Science. 2007; 315 (5820): 1846-1850.

24. Baum JK, Worm B. Cascading top down effects of changing oceanic predator abundances. J Animal Ecol. 2009; 78 (4): 699-714.

25. Essington TE, Beaudreau AH, Wiedenmann J. Fishing through marine food webs. Proc Natl Acad Sci USA. 2006; 103 (9): 3171-3175.

26. Heithaus MR, Frid A, Vaudo JJ, Worm B, Wirsing AJ 2010. Unraveling the ecological importance of elasmobranchs. In: Carrier JC, Musick J, Heithaus MR (eds) Biology of Sharks and their relatives II. CRC Press, Boca Raton. 611-637.

27. Pauly D, Christensen V, Dalsgaard J, Froese R, Torres Jr F. Fishing down marine food webs. Science. 1998; 279 (5352): 860-863.

28. Rogers SI, Ellis JR. Changes in the demersal fish assemblages of British coastal waters during the 20th century. ICES J Mar Sci. 2000; 57 (4): 866-881.

29. Molfese C, Beare D, Hall-Spencer JM. Overfishing and the Replacement of Demersal Finfish by Shellfish: An Example from the English Channel. PLoS One. 2014; 9 (7): e101506.

30. Pauly D, Palomares ML, Froese R, Sa-a P, Vakily M, Preikshot D, et al. Fishing down Canadian aquatic food webs. Can J Fish Aquat Sci. 2001; 58 (1): 51-62.

31. Pauly D, Watson R. Background and interpretation of the 'Marine Trophic Index' as a measure of biodiversity. Philos Trans R Soc B. 2005; 360 (1454): 415-423.

32. Freire KMF, Pauly D. Fishing down Brazilian marine food webs, with emphasis on the east Brazil large marine ecosystem. Fish Res. 2010; 105 (1): 57-62.

33. Liang C, Pauly D. Fisheries impacts on China's coastal ecosystems: Unmasking a pervasive 'fishing down' effect. PLoS One. 2017; 12 (3): e0173296.

34. Erlandson JM, Rick TC, Braje TJ. Fishing up the Food Web?: 12,000 Years of Maritime Subsistence and Adaptive Adjustments on California's Channel Islands. Pac Sci. 2009; 63 (4): 711-724.

35. Litzow MA, Urban D. Fishing through (and up) Alaskan food webs. Can J Fish Aquat Sci. 2009; 66 (2): 201-211.

36. Branch TA. Fishing Impacts on Food Webs: Multiple Working Hypotheses. Fisheries. 2015; 40: 373-375.

37. Sethi SA, Branch TA, Watson R. Global fishery development patterns are driven by profit but not trophic level. PNAS. 2010; 107, 12163-12167.

38. Branch TA, Watson R, Fulton EA, Jennings S, McGilliard CR, Pablico GT, et al. The trophic fingerprint of marine fisheries. Nature, 2010; 468 (7322): 431-435.

39. Stergiou K, Tsikliras A. Fishing down, fishing through and fishing up: fundamental process versus technical details. Mar Ecol Prog Ser. 2011; 441: 295-301.

40. Salas S, Chuenpagdee R, Seijo JC, Charles A. Challenges in the assessment and management of small-scale fisheries in Latin America and the Caribbean. Fish Res. 2007; 87 (1): 5-16.

41. Begossi A, Silvano RAM. Ecology and ethnoecology of dusky grouper [garoupa, *Epinephelus marginatus* (Lowe, 1834)] along the coast of Brazil. J Ethnobiol Ethnomed. 2008; 4 (1): 20.

42. Lima EG, Begossi A, Hallwass G, Silvano RAM. Fishers' knowledge indicates short-term temporal changes in the amount and composition of catches in the southwestern Atlantic. Mar Policy. 2016; 71, 111-120.

43. Sáenz-Arroyo A, Roberts CM, Torre J, Cariño-Olvera M, Hawkins JP. The value of evidence about past abundance: marine fauna of the Gulf of California through the eyes of 16th to 19th century travellers. Fish Fish. 2006; 7 (2): 128-146.

44. Martins IM, Medeiros RP, Domenico MD, Hanazaki N. What fishers' local ecological knowledge can reveal about the changes in exploited fish catches. Fish Res. 2018; 198, 109-116.

45. Giglio VJ, Suhett AC, Zapelini CS, Ramiro AS, Quimbayo JP. Assessing captures of recreational spearfishing in Abrolhos reefs, Brazil, through social media. Reg Stud Mar Sci. 2020; 34, 100995.

46. Braga HO, Azeiteiro UM, Oliveira HMF, Pardal MA. Conserving Brazilian Sardine: Fisher's attitudes and knowledge in the Marine Extractive Reserve of Arraial do Cabo, Rio de Janeiro State, Brazil. Fish Res. 2018; 204, 402-411.

47. Taylor SM, Braccini JM, Bruce BD, McAuley RB. Reconstructing Western Australian white shark (*Carcharodon carcharias*) catches based on interviews with fishers. Mar Freshwater Res. 2018; 69 (3): 366-375.

48. Baum JK, Myers RA. Shifting baselines and the decline of pelagic sharks in the Gulf of Mexico. Ecol Lett. 2004; 7 (2): 135-145.

49. Soga M, Gaston KJ. Shifting baseline syndrome: causes, consequences, and implications. Front Ecol Environ. 2018; 16 (4): 222-230.

50. Pauly D. Anecdotes and the shifting baseline syndrome of fisheries. Trends Ecol Evol. 1995; 10 (10): 430.

51. Papworth SK, Coad JRL, Milner- Gulland EJ. Evidence for shifting baseline syndrome in conservation. Conserv Lett. 2009; 2 (2): 93-100.

52. Thurstan RH, McClenachan L, Crowder LB, Drew JA, Kittinger JN, Levin PS. Filling historical data gaps to foster solutions in marine conservation. Ocean Coastal Manage. 2015; 115, 31-40.

53. Bender MG, Floeter SR, Hanazaki N. Do traditional fishers recognise reef fish species declines? Shifting environmental baselines in Eastern Brazil. Fish Manag Ecol. 2013; 20 (1): 58-67.

54. Bender MG, Machado GR, Azevedo PJS, Floeter SR, Monteiro-Netto C, Luiz OJ, et al. Local Ecological Knowledge and Scientific Data Reveal Overexploitation by Multigear Artisanal Fisheries in the Southwestern Atlantic. PloS One. 2014; 9 (10): e110332.

55. Reis-Filho JA, Freitas RHA, Loiola M, Leite L, Soeiro G, Oliveira HHQ, et al. Traditional fisher perceptions on the regional disappearance of the largetooth sawfish *Pristis pristis* from the central coast of Brazil. Endang Species Res. 2016; 29 (3): 189-200.

56. Silvano RAM, MacCord PFL, Lima RV, Begossi A. When Does this Fish Spawn? Fishermen’s Local Knowledge of Migration and Reproduction of Brazilian Coastal Fishes. Environ Biol Fishes. 2006; 76 (2-4): 371-386.

57. Silvano RAM, Begossi A. Fishermen's local ecological knowledge on Southeastern Brazilian coastal fishes: contributions to research, conservation, and management. Neotr Ichthyol. 2012; 10 (1): 133-147.

58. Barbosa-Filho MLV, Schiavetti A, Alarcon DT, Costa-Neto EM. “Shark is the man!”: ethnoknowledge of Brazil’s South Bahia fishermen regarding shark behaviors. J Ethnobiol Ethnomed. 2014; 10 (1): 54.

59. Silvano RAM, Begossi A. What can be learned from fishers? An integrated survey of fishers’ local ecological knowledge and bluefish (Pomatomus saltatrix) biology on the Brazilian coast. Hydrobiologia. 2010; 637(1), p.3.

60. Sáenz-Arroyo A, Roberts CM, Torres J, Cariño-Olvera M. Using fishers’ anecdotes, naturalists’ observations and grey literature to reassess marine species at risk: the case of the Gulf grouper in the Gulf of California, Mexico. Fish Fish. 2005; 6 (2): 121-133.

61. Bevilacqua AHV, Carvalho AR, Angelini R, Christensen V. More than Anecdotes: Fishers’ Ecological Knowledge Can Fill Gaps for Ecosystem Modeling. PLoS One. 2016; 11 (5): e0155655.

62. Sánchez-Jiménez A, Fujitani M, MacMillan D, Schlüter A, Wolff M. Connecting a Trophic Model and Local Ecological Knowledge to Improve Fisheries Management: The Case of Gulf of Nicoya, Costa Rica. Front. Mar. Sci. 2019; 6:126.

63. Gerhardinger LC, Godoy EAS, Jones PJS. Local ecological knowledge and the management of marine protected areas in Brazil. Ocean Coast Manage. 2009; 52 (3-4): 154-165.

64. Wiber MG, Young S, Wilson L. Impact of Aquaculture on Commercial Fisheries: Fishermen’s Local Ecological Knowledge. Hum Ecol Interdiscip J. 2012; 40 (1): 29-40.

65. Damasio LDMA, Lopes PFM, Guariento RD, Carvalho AR. Matching Fishers' Knowledge and Landing Data to Overcome Data Missing in Small-Scale Fisheries. PLoS One. 2015; 10 (7): e0133122.

66. Aylesworth L, Phoonsawat R, Suvanachai P, Vincent ACJ. Gaenerating spatial data for marine conservation and management. Biodivers Conserv. 2017; 26 (2): 383-399.

67. Santos RO, Rehage JS, Kroloff EKN, Heinen JE, Adams AJ. Combining data sources to elucidate spatial patterns in recreational catch and effort: fisheries-dependent data and local ecological knowledge applied to the South Florida bonefish fishery. Environ Biol Fishes. 2019; 102 (2): 299-317.

68. Melo JCF, Kruel VSF, Hanazaki N. Árvores e madeiras na cultura naval tradicional Bailey, KD. 1994. Methods of Social Research. New York: The Free Press/Macmillan Publishers. 588; 2019.

69. Carvalho WFD, Gonzalez Rodriguez E. Development of primary and bacterial productivity in upwelling waters of Arraial do Cabo region, RJ (Brazil). Braz J Oceanogr. 2004; 52 (1): 35-45.

70. Cordeiro CAMM, Mendes TC, Harborne AR, Ferreira CEL. Spatial distribution of nominally herbivorous fishes across environmental gradients on Brazilian rocky reefs. J Fish Biol. 2016; 89 (1): 939-958.

71. Mendonça, T.C.M., de Moraes, E.A., Costa, M.A.M., 2013. Turismo e pesca nas Reservas Extrativistas Marinhas de Arraial do Cabo (RJ) e da Prainha do Canto Verde (CE): possibilidades e limites de complementaridade. Caderno Virtual de Turismo. 2013; 13 (3), 372-390.

72. Bailey, KD, 1994. Methods of Social Research. New York: The Free Press/Macmillan Publishers. 588.

73. Maia HA, Morais RA, Siqueira AC, Hanazaki N, Floeter SR, Bender MG. Shifting baselines among traditional fishers in São Tomé and Príncipe islands. Ocean Coast Manage. 2018; 154, 133-142.

74. Pauly D, Froese R. Comments on FAO's State of Fisheries and Aquaculture, or ‘SOFIA 2010’. Mar Policy. 2012; 36 (3): 746-752.

75. Palomares MLD, Pauly D. Editors. 2020. SeaLifeBase. World Wide Web electronic publication. Available from: www.sealifebase.org, version (07/2020).

76. Dulvy NK, Sadovy Y, Reynolds JD. Extinction vulnerability in marine populations. Fish Fisheries. 2003; 4: 25-64.

77. Pauly D, Christensen V, Guénette S, Pitcher TJ, Sumaila UR, Walters CJ, et al. Towards sustainability in world fisheries. Nature. 2002; 418 (6898): 689-695.

78. R Development Core Team. 2019. R: A language and environment for statistical computing. 418 Vienna, Austria: R Foundation for statistical Computing.

79. Harris AN, Poiner IR. Changes in species composition of demersal fish fauna of Southeast Gulf of Carpentaria, Australia, after 20 years of fishing. Mar Biol. 1991; 111: 503-519.

80. Jin X, Tang Q. Changes in fish species diversity and dominant species composition in the Yellow Sea. Fisheries Research. 1996; 26: 337-352.

81. Fogarty MJ, Murawski SA. Large-scale disturbance and the structure of marine systems: fishery impacts on georges bank. Ecological Applications. 1998; 8: S6-S22.

82. Pincinato RBM, Gasalla MA. Priceless prices and marine food webs: Long-term patterns of change and fishing impacts in the South Brazil Bight as reflected by the seafood market. Progress in Oceanography. 2010; 87: 320-330.

83. Lovell S, Johnson AE, Ramdeen R, McClenachan L. Shifted baselines and the policy placebo effect in conservation. Oryx. 2018; 54 (3): 383-391.

84. Begossi A, Salivonchyk SV, Barreto T, Nora V, Silvano RAM. Small-scale Fisheries and Conservation of Dusky Grouper (Garoupa), *Epinephelus marginatus* (Lowe, 1834) in the Southeastern Brazilian Coast. Sci J Agric Res Manag. 2012; 2012:4.

85. Ferreira HM, Reuss-Strenzel GM, Alves JA, Schiavetti A. Local ecological knowledge of the artisanal fishers on *Epinephelus itajara* (Lichtenstein, 1822) (Teleostei: Epinephelidae) on Ilhéus coast-Bahia State, Brazil. J Ethnobiol Ethnomed. 2014; 10 (1): 51.

86. Seyboth E, Condini MV, Albuquerque CQ, Varela Jr AS, Velasco G, Vieira JP, et al. Age, growth, and reproductive aspects of the dusky grouper *Mycteroperca marginata* (Actinopterygii: Epinephelidae) in a man-made rocky habitat in southern Brazil. Neotrop Ichthyol. 2011; 9 (4): 849-856.

87. Condini MV, García-Charton JA, Garcia AM. A review of the biology, ecology, behavior and conservation status of the dusky grouper, *Epinephelus marginatus* (Lowe 1834). Rev Fish Biol Fish. 2018; 28 (2): 301-330.

88. Sadovy de Mitcheson Y, Craig MT, Bertoncini AA, Carpenter KE, Cheung WWL, Choat JH, et al. Fishing groupers towards extinction: a global assessment of threats and extinction risks in a billion dollar fishery. Fish Fish. 2013; 14(2): 119-136.

89. Duarte MR, Tubino RA, Monteiro-Neto C, Martins RRM, Vieira FC, Andrade-Tubino MF et al. Genetic and morphometric evidence that the jacks (Carangidae) fished off the coast of Rio de Janeiro (Brazil) comprise four different species. Biochem Syst Ecol. 2017; 71, 78-86.

90. Ceretta BF, Fogliarini CO, Giglio VJ, Maxwell MF, Waechter LS, Bender MG. Testing the accuracy of biological attributes in predicting extinction risk. Perspect Ecol Conserv. 2020; 18 (1): 12-18.

91. Bhathal B, Pauly D. 'Fishing down marine food webs' and spatial expansion of coastal fisheries in India, 1950-2000. Fish Res. 2008; 91 (1): 26-34.

92. Ding Q, Chen X, Yu W, Tian S, Chen Y. An evaluation of underlying mechanisms for "fishing down marine food webs". Acta Oceanol Sin. 2016; 35 (8): 32-38.

93. Blanchard JL, Coll M, Trenkel VM, Vergnon R, Yemane D, Jouffre D, et al. Trend analysis of indicators: a comparison of recent changes in the status of marine ecosystems around the world. ICES J Mar Sci. 2010; 67 (4): 732-744.

94. Coll M, Shannon LJ, Yemane D, Link JS, Ojaveer H, Neira S, et al. Ranking the ecological relative status of exploited marine ecosystems. ICES J Mar Sci. 2010; 67 (4): 769-786.

95. Liang C, Pauly D. Masking and unmasking fishing down effects: The Bohai Sea (China) as a case study. Ocean Coast Manag 2020; 184, 105033.

96. Foley CMR. Management implications of fishing up, down, or through the marine food web. Mar Policy. 2013; 37: 176–182.

97. Francini-Filho RB, De Moura RL. Dynamics of fish assemblages on coral reefs subjected to different management regimes in the Abrolhos Bank, eastern Brazil. Aquat Conserv Mar Freshwater Ecosyst. 2008; 18 (7): 1166.

98. Roos NC, Pennino MG, De Macedo Lopes PF, Carvalho AR. Multiple management strategies to control selectivity on parrotfishes harvesting. Ocean Coast Manage. 2016; 134, 20-29.

99. Floeter SR, Halpern BS, Ferreira CEL. Effects of fishing and protection on Brazilian reef fishes. Biol Conserv. 2006; 128 (3): 391-402.

100. Fouzai N, Coll M, Palomera I, Santojanni A, Arneri E, Christensen V. Fishing management scenarios to rebuild exploited resources and ecosystems of the Northern-Central Adriatic (Mediterranean Sea). J Mar Syst. 2012; 102, 39-51.

101. MMA, 2018. Instituto Chico Mendes de Conservação da Biodiversidade. Livro Vermelho da Fauna Brasileira Ameaçada de Extinção: Volume VI - Peixes. In: Instituto Chico Mendes de Conservação da Biodiversidade (Org.). Livro Vermelho da Fauna Brasileira Ameaçada de Extinção. Brasília: ICMBio. 1232p. http://www.icmbio.gov.br. Download em 20 maio 2018.

102. Paterson B. Integrating fisher knowledge and scientific assessments. Anim Conserv. 2010; 13 (6): 536-537.

Supporting Information

S1 Table. Trophic level (TL) of species landed in Arraial do Cabo, from 1992 to 2008. Trophic levels were obtained from FishBase database where a higher value means higher trophic levels (Froese and Pauly, 2019).

S2 Table. Parameters of the Poisson Generalized linear model for the number of species cited by fishers based on years of practice.

S3 Table. Fishing effort of the artisanal fishing fleet of Arraial do Cabo, Rio de Janeiro, Brazil.

S1 Fig Differences in mean trophic level (MTL) of overexploited and new target species cited by local fishers. Observed MTL (red diamond) are contrasted against null MTL values (gray circles), generated through randomization of citation frequency of species within each category.

S2 Fig Fishing effort in number of boats (a) and fishing days (b) over the years for Arraial do Cabo, Rio de Janeiro, Brazil.

S1 File. Questionnaire applied in interviews with fishers in Arraial do Cabo.

Response to Reviewers

Reviewer #1: The manuscript presents a careful analysis and data, although several of them do not present relations as convincing as suggested in the text. However, the manuscript presents so much results and approaches without any novelty for an international audience. It is shown by data from formal science and traditional knowledge that fisheries initially reach the highest trophic levels, that perceptions change over time, that fishing targets are being changed. This is the description of a historical process of any artisanal fisheries in the world. It is not clear what are the innovative aspects in the article that would justify its publication. Considering these aspects, I cannot recommend the publication of the article in a journal such as PLOS ONE

R. We respect Reviewer #1 for his opinion and understand that this is a common pattern reported for artisanal fisheries across the world. Yet there are few studies in which Local Ecological Knowledge, from traditional communities, has been contrasted with landing data to reveal matching patterns in local fisheries (Silvano et al. 2009, Ferreira et al. 2014, Herbst & Hanazaki, 2014, Silva et al. 2020). Considering this aspect, at the best of our knowledge, the present study is the first to assess exploitation status of fish species caught by artisanal fishing and changes in target species over time in the southwestern Atlantic. We highlight that the study area is a partially-protected marine area, whose main resource managers are the extractive community. Here, we combine the knowledge of fishers with landings data, to understand the dynamics of fish stocks from the past to the present. Thus, we generate information that can be used to propose conservation and management measures. We point out that there is no information of temporal changes in fishery targets along Brazilian coast. Such information is not only of ecological importance, but also cultural and economic. Also, there are several examples of local scale studies published in PLOS ONE, that are not necessarily of broad interest (ttps://journals.plos.org/plosone/article?id=10.1371/journal.pone.0236146)

(https://journals.plos.org/plosone/article?id=10.1371/journal.pone.0228976)

Reviewer #2: Review of de Oliveira et al. Fishing down the food web inferred from local ecological knowledge and landing data in the Southeastern Brazilian coast

De Oliveira et al used traditional ecological knowledge from fishers in a coastal community of subtropical Brazil (Arraial do Cabo) and historical landing statistics to identify species overexploitation patterns, changes in target fishery resources and trophic levels. Using information from 155 interviews made to fishers and the landing statistics from that area during 1992 and 2008, the authors tried to identify which species according to the perception of fishers were overexploited and which species were new components of the fishery. Additionally, the authors calculated the mean trophic level of the annual landings in that area to verify if the fishing down the food web hypothesis would hold true for that area.

There is a vast amount of literature that has combined TEK with conventional fisheries information to complement our knowledge of a specific fishery. The present study is one of those studies and is a welcome contribution to this relatively new field. I am more critical about how the authors have tried to show their results especially with respect to the fishing down the food web hypothesis and in a lesser degree to the shifting baseline paradigm.

My impression from the data shown is that there is no reason to believe that there is a fishing down the food web pattern in the study system, at least with the information (MTL) that the authors are analyzing. Despite there are a lot of studies that have pointed to a shifting baseline situation in fisheries, where younger fishers won't recognize easily the degraded state of fishery resources, the present study in my opinion does not show that clear pattern (by inspecting Fig 2a,c). This is totally fine as a result and there is no reason to try to adjust the argument to the prevailing paradigm.In my opinion, the most interesting finding of the paper is the change in target species pointed out by fishers. This is something that could be highlighted much more in the manuscript. Discussion of the implications of this with similar results in other parts of the world would make the manuscript much stronger.

Having said this, I welcome this contribution to the field, but I disagree with the main interpretation of the data presented in the manuscript. I therefore recommend the authors to reconsider many of the interpretations of their findings in light of what their actual data

show. After these major criticisms are addressed I think that this manuscript is not suitable for publication in a PloS ONE.

See specific comments below.

R: We agree that the fishing down the food web process was not detected when we analyzed the MTL of all landed species. However, this process may not be easily detected due to the influence of factors that may mask its occurrence. Some factors that may mask fishing down are taxonomic over aggregation of catch statistics and the spatial expansion of fishing, but that can be clarified by local experts. Also, the masking effect can occur when both the proportion of high TL species and pelagic species of low TL are declining. In our case, we identified declines in landings of the two main landed species, largehead hairtail (*Trichiurus lepturus*) and the sardine (*Sardinella brasiliensis*) (see figures below). Landings of *T. lepturus*, a high TL species, had an increase from the 1990s to 2000s, followed by a decrease in landings after the 2000s. The same decline trend was identified for the sardine, the main species landed in the region. Declining trends in landings of these species are possibly causing an increase in the MTL of all species, as demonstrated in other studies (Blanchard et al. 2010, Coll et al. 2010). In this case, the masking effect is named as "false fishing up" and we discussed the possible occurrence of this process in our study (lines 440 - 447). Another important result that suggests the overexploitation scenario was showed through landings of high TL species (TL> 4) that decreased significantly over time (Fig. 5c, d). For some stocks, the reduction exceeded 80% (Fig. 4a, c, e, g). In addition, we asked fishers about the status of fishing stocks, and the answers to this question were not added to our manuscript, but it is important to highlight that 92% of fishers reported a decline in fishing stocks, and most of them reported that fish stocks decrease from 50 to 80% (see figure below). Finally, the overexploitation scenario of marine resources has been supported by another local study (Bender et al. 2014) based on fisher's knowledge, underwater visual census and scientific data. The authors also identified declines in high and low TL species caught by artisanal fisheries.

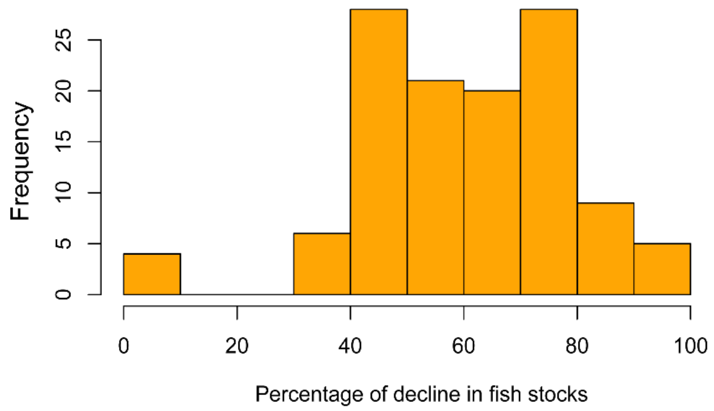

Frequency
0
5
10
15
20
25
0
20
40
60
80
100
Percentage of decline in fish stocks

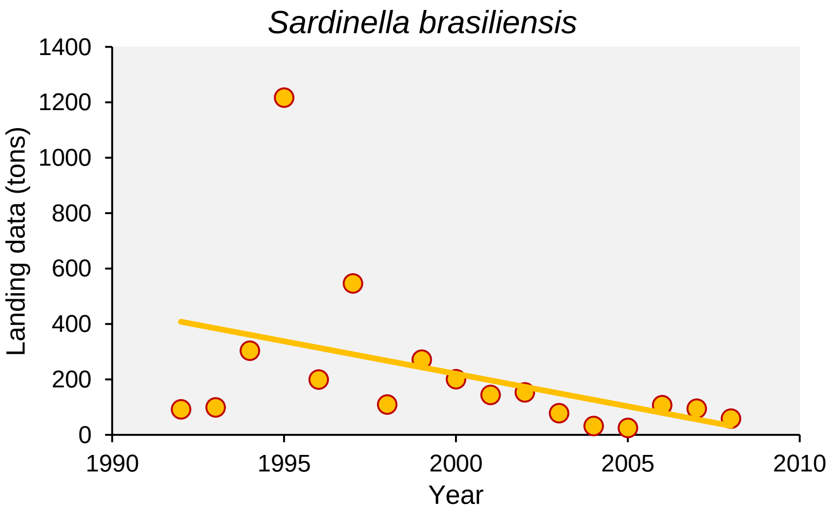

Sardinella brasiliensis
Landing data (tons)
0
200
400
600
800
1000
1200
1400
1990
1995
2000
2005
2010
Year

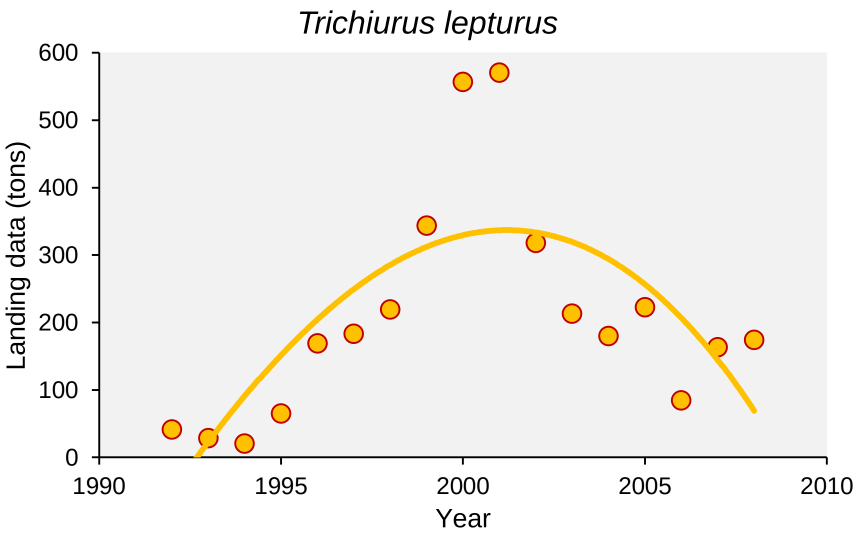

Trichiurus lepturus
Landing data (tons)
0
100
200
300
400
500
600
1990
1995
2000
2005
2010
Year

With respect to the shifting baseline results, we understand the reason for his argument. The distribution of the number of citations does not vary so much between years of fishing practice (fig. 2 a, c). However, we showed that there were different percent increase in citations as fishers' experience increase (Fig. 2 b, d). Another evidence of the shifting baseline occurs when there are significant differences in the proportion of citations between experience categories (Fig. 3). Very experienced fishers identified *Epinephelus marginatus, Caranx hippos,* and *Balistes capriscus* as overexploited and new target species more frequently than the less experienced ones (Fig. 3).

Abstract

L36. Clarify is you are referring to landings or to catches. These are two different concepts. Look at differences here: http://www.fao.org/3/bt981t/bt981t.pdf

R: Thank you for your comment. We changed this expression along to manuscript. In our case, the correct term is "landings" because it represents the quantities landed as recorded at the time of landing as whole or eviscerated fish, fillets, livers, roes, etc.

L55. Provide a more specific sentence on the problem of sustainability in fisheries. The recent FAO SOFIA reports clearly define and provide a historical perspective on the percentage of fish stocks that are fished at biologically sustainable levels since the 70s.

R: We added the following sentence: "The status assessment of fishery resources revealed that the fraction of fish stocks that are within biologically sustainable levels decreased from 90% in 1974 to 65.8% in 2017 (FAO, 2020)."

L61-62. Not all fisheries are directed toward top predators. This is a misleading statement. Modify sentence or bring clarification to what you actually mean.

R: We agree with your statement and modified the sentence (Lines 62 - 65).

L71. The “fishing down the food web“ paradigm has been continuously revised since it was first proposed. I would suggest that the authors consider at least mentioning in the introduction the critics of the paradigm. For this, I would recommend to revise Branch et al. (2010) The trophic fingerprint of marine fisheries. Nature 468, 431–435 and Sethi et al (2010) Global fishery development patterns are driven by profit but not trophic level. PNAS 107, 12163-12167.

R: We have included the following explanation: “In addition, studies criticize the exclusive use of MTL as an indicator to track changes in the ecosystem (Branch et al. 2010). As well as there may be multiple hypotheses (Branch et al. 2015), which explain how fishing affects marine food webs, such as profit-driven fishing (Sethi et al. 2010).”

L73. Change “trophic” for “food”.

R: The term was replaced. (Line 74).

L72-79. See comment from L71. Maybe rephrasing the whole paragraph reflecting that there are many ways how fishing can affect marine ecosystems (i.e. not only by reducing trophic level), would be the way to go.

R: We agreed and added an explanation in lines 73 - 84.

L93. Do you mean unexplored or unexploited?

R: We mean "unexplored state". We clarified it in the text.

L109. Change “have” for “has”

R: Replaced accordingly.

L109-114. A number of recent studies have used LEK to link to trophic models. This application may be worth including here and probably somewhere in the discussion. Just a few examples:

Bevilacqua AHV, Carvalho AR, Angelini R, Christensen V (2016) More than Anecdotes: Fishers' Ecological Knowledge Can Fill Gaps for Ecosystem Modeling. PLoS ONE 11(5): e0155655. https://doi.org/10.1371/journal.pone.0155655

Sánchez-Jiménez A, Fujitani M, MacMillan D, Schlüter A and Wolff M (2019) Connecting a Trophic Model and Local Ecological Knowledge to Improve Fisheries Management: The Case of Gulf of Nicoya, Costa Rica. Front. Mar. Sci. 6:126. doi: 10.3389/fmars.2019.00126

R: We added these studies in lines 112 - 116.

L120. Can you specify what is the MTL approach?

R. Done. Lines 123 - 124.

Also, I am not sure if you can link possible changes in landings trophic structure exclusively to fishing impacts. There may be other reasons associated these to changes. Provide some clarification on how are you able to assign the possible changes just to fishing.

R: First, we observed through information from fishers that a large part of the resources identified as overexploited were high trophic level species. This finding motivated us to try to understand how fishing is affecting the local ecosystem. Second, the temporal replacement of target species caught our attention –as the case of largehead hairtail (*T. lepturus*)– which became a new resource, and posteriorly, revealed declining trends. Finally, to track changes in the trophic structure, we used landing data to calculate the mean trophic level index of landed species. We verified MTL trends in four groups: all landed species, species with TL > 4, TL ≥ 3.5 and TL < 3.5. We identified significant decline trends in MTL of species with TL > 4 and TL ≥ 3.5, while the MTL of all landed species and TL < 3.5 showed non-significant increase trends. Although the MTL of all species had no decline, we noticed that

when the sardine and largehead hairtail are removed, there is a decline over time. The sardine is the main species landed in the period, followed by *P. saltatrix* and *T. lepturus*. The approach of excluding the sardine (low TL) and *T. lepturus* (high TL) from the is based on previous studies (Alleway et al. 2014). Studies have shown that fishing down can be masked due to declines in small pelagic species with low TL and marked by declines of large predators in the past (Blanchard et al. 2010, Coll et al. 2010). We suggest that this masking effect is a result from the overexploitation of sardine and *T. lepturus*.

Also, we checked the landing trends for species reported by fishers as overexploited and new targets. This approach helped us to identify significant declines in landings of species with high trophic levels (TL = 4 and TL ≥ 3.5) (figure 5a, b). In addition, the declining trends for high trophic level species were also observed through landings data (Kg) (figure 5c), and separately for the main species landed (figure 5d).

Materials and methods

L129-130. It is not the artisanal fisheries the one making trips. It is the fishermen those making the trips. Correct wording there.

R: Corrected accordingly (Lines 133 - 135).

L131. Clarify the meaning of the word secular in this context.

R: Secular means that this fishing activity has been developed for over a century in the region. Studies show that beach seine fishing is practiced with old fishing canoes, which were built by the first generations of fishers in Arraial do Cabo, since 1880. This information is a book entitled "Arraial 500" and was clarified in the text (Line 135).

L151. Snowball sampling has been suggested to sometime bias results of interviews. See:

Kirchherr J, Charles K (2018) Enhancing the sample diversity of snowball samples: Recommendations from a research projecton anti-dam movement in Southeast Asia. Plos ONE 13(8): e0201710.https://doi.org/10.1371/journal.pone.0201710

Explain how do you overcome these limitations or acknowledge the limitations of your strategy.

R: In fact, we used snowball sampling to complete our sample number. At the fishing pier, we approached fishers at random, and they were invited to participate in our research. In addition, we had the help of an experienced fisher known to the community. With this contact, it was possible to access a large number of interviewees and it mainly facilitated the process of interviews. Kirchherr & Charles (2018) recommend strategies to overcome snowball sampling limitations, as having previous personal contacts before starting snowball sampling. We therefore did not rely exclusively on snowball sampling.

L155. I would not call a beginner a fisher that has been fishing 15 years. Maybe change the name of this first category.

R: Thank you for the suggestion. We modified that term into "less experienced".

L156-157. What is the reasoning to differentiate fishers with 36-40yr of experience from those with >40 yr.? I am not arguing for a different categorization, but the reasoning behind this choice needs to be clear.

R: We chose these experience categories to accommodate similar sample sizes. Thus, there is a better distribution of the number of interviewees in each experience category. In addition, experience categories were based on previous studies (e.g. Maia et al. 2018). We have included an explanation in lines 164 – 173.

L173. Landings and catch are two different terms. Be clear in what data you actually obtained. See comment from above.

R: We have replaced "catches" by "landings".

L186-188. Revise this sentence. It is not clear what you are trying to say.

R: We have rephrased this fragment, see lines 196 – 198.

L196. Is "citations" the right word here?

R: Yes, we consider that the correct form is "citations" because it is the frequency at which a species was mentioned by the fishers as overexploited and new target species.

L212. The normal term is "count data"

R: Done. Line 224.

Results

L232-239. By looking at the distribution of your data points in Fig2a and 2c it is difficult to find a good fit for any model there. Can you provide a measure of the goodness of fit of your model? Something like AIC or a pseudo-R? include a table with the results of your GLM in the ms or in the Supplement.

R: As suggested by the reviewer, we added a table with the results of GLM (Please, see table 1).

L300-302. Fisheries landings data does not necessarily reflect the stock status. Work of Trevor Branch and co-workers among others has repeatedly shown the weaknesses of using catch data to determine stock status. This needs appropriate acknowledgement in the manuscript.

R: We agree with your comment, the study by Branch and collaborators was included in lines 80 - 83, this point was also argued in lines 439 – 448, in which we discussed that MTL index may not always reveal the true status of stocks. However, our work uses as a prior knowledge the information from local fishers, who reported

that overexploited species such as *Pomatomus saltatrix* and *Trichiurus lepturus*, among many others, species whose declines have also been evidenced through landing data. In general, when fishers were asked about the status of fishing stocks, 92% of fishers reported a decline in stocks of target species. In addition, fishers were asked about the percentage of declines, and the highest percentages are between 50% and 80% (see figure in the answer above). This finding, added to the declining trends in landings of high trophic level species suggest the overexploitation scenario of fisheries in Arraial do Cabo. Old photographs help fishers' remember the past composition and abundance of landed species, and visualize change in fisheries (Fig. 6). These photographs reveal catches with large predators such as groupers and sharks, which have become rare events in the region. Photographs can be valuable historical information sources. In previous studies, old photographs were used to estimate decreases in the maximum individual fish size and the proportion of large groupers caught in South Florida, USA (McClenachan, 2009).

L323-324. This statement is problematic. To my understanding your interviews did not ask about temporal trends to be able to compare those directly with the landings data that you are presenting. Please clarify how you are making these comparisons.

R: Thank you for your comment. Indeed our interviews did not ask fishers on the past vs. present day catches of each species, but solely on which fish species they considered overexploited, and which were new target species, based on their accumulated experiences. Our questionnaire was organized to detect overexploited and new target species, which were then compared to temporal trends in local landings. We understand that landing trends are similar to the species identified as overexploited by fishers, where we can see declines in landings, and also for new target species, for which landings increase (as they become new targets) and are followed by a decrease.

Discussion

L352-353. By looking at your Fig2a and 2c, I would argue that there is no clear pattern to make such a statement.

R: This sentence refers to the increase in the number of species reported by fishers as years of practice increase. We identified this pattern in figure 2a, c and through the GLM results, which show significant increases in the number of species cited as overexploited (*P* = 0.003) as well as for the number of species identified as new target species (*P* = 0.014) across years of practice. We understand that the variance around the number of species mentioned by fishers across experience categories gives a sense of no clear pattern, yet our model has revealed there are slight increases across each category.

L396-398. Again, your Fig 5a, does not fully support your statement.

R: We agree that figure 5a does not fully support our argument for the occurrence of this phenomenon in Arraial do Cabo. For clarification, we have corrected this statement in the fragment (lines 429 - 432) and throughout the manuscript. We also included a case study that showed the change in target species from demersal finfish to shellfish, which identified the fishing down process through a long series of landing data (1920 - 2010) and MTL. In our study, we used a short series of landing data (1992 - 2008) in combination with fisher's knowledge. Despite the short series of landing data, we interviewed 32 very experienced fishers (with practice time between 40 - 60 years) who also reported declines in fish stocks. In addition, landing data show significant declines in species with TL > 4 (Fig. 5c, d). Finally, we are cautious in affirming the occurrence of fishing down in Arraial do Cabo, although there is the possibility that it may be masked by other issues.

L402-404. From what I got from your methods, you used the landings data to derive mean trophic level of the landings per year. Less clear to me is how you are incorporating LEK into that analysis. Almost all the most important new fishery species identified by the fishers are high trophic level (>4). And the most important, *T. lepturus* is 4.4.

R: We agree that most of the new target species reported by fishers are of a high trophic level, such as *T. lepturus* (TL = 4.4) and *B. capriscus* (TL = 4.1). The fisher's knowledge helped us to identify the declines in top predators such as *Pomatomus saltatrix* (TL = 4.5), *Trichiurus lepturus* (TL = 4.4), *Epinephelus marginatus* (TL =

4.4), *Seriola fasciata* (TL = 4.5), and *Caranx hippos* (TL = 3.6). Yet, fishers reported a scenario of overfishing in the region, which 92% of them noticed alarming declines in stocks of the main target species of fishing. Another way to incorporate the fisher's knowledge in our analysis was comparing the MTL of overexploited with MTL of new target species mentioned by fishers (see Supporting information, Fig. S1). We observed that the citations of overexploited species are more frequently within higher TL species than new target species (see Supporting information, Fig. S1). In addition, the MTL observed for overexploited species was higher than expected at random.

L409-416. This sounds as if you would like to prove that fishing down the food webs is occurring in your study site, but your data does not tell that is actually happening. You provide alternative explanations from other areas but don't provide an explanation or analysis for your case.

R: Thank you for your comment. We have rewritten this paragraph (Lines 439 - 448).

L418-419. Fig 6 show very impressive pictures, but your LEK study did not ask questions about tunas or sharks and their historical catches.

R: The shark species of the *Carcharhinus* genus have been cited by fishers as overexploited and new target species (see tables 2 and 3), as it is the case for *C. brevipinna* (Figure 6). Our mention of shark and tuna catches merely intended to offer the reader an insight into catches' composition at different time periods, as previous studies have identified declines in large predators in the region (Bender et al. 2014).

L425-430. Why *Scarus trispinosus* did not appear in your LEK results? Fishers did not recognize this species as overexploited. Also this whole section does not discuss anything related to your study.

R: Thank you for your comment. *S. trispinosus* did not appear in our results because this species is mainly a target of spearfishers. For this fishing gear, we interviewed

two local fishers, and one of them mentioned the species *Sparisoma axillare* as a new target species. Another point that explains why this species was not mentioned by fishers is that, unlike in our study, Bender et al. (2014) asked questions directed towards parrotfish and grouper species, using photographs. Indeed, the collapse of parrotfishes in the same region is pretty well covered in Bender et al. (2014).

L436-440. By looking at your supplement data, the number of fishing boats has been reduced to around half of those reported in 1995. Fishing days in contrast have more than doubled. The average number of fishing days per boat per year in 1995 was only 5 days. In 2019 this value is 25 days per year. The 25 days value is still very low for a year. What are the explanation that you have for this? Are the methodologies to calculate these numbers comparable among reports?

R: We agree with your comment and removed that sentence (lines 468 - 471). In figure and table S2, the increase in fishing days corresponds to the most recent reports from PMAP-BS, both accounting for the fishing days of all fishing gear operating in Arraial do Cabo. These recent reports also consider gear that does not use boats, e.g. hand line, manual collection and some types of fishing nets, as beach seine. Although these reports used different methodologies to estimate the number of fishing days, we can still consider the number of boats as the fishing effort to compare the causes reported by fishers.

L448. In the manuscript you do not present any information about those management initiatives and how you relate those to your actual work

R: The only management initiatives existent in the local marine extractive reserve is regarding the spawning period for some species and few species prohibition. Indeed, there are no initiatives for stock recovering based on data produced locally. Additionally, local managers are poorly skilled to absorb state and national management rules to be locally applied and enforced.

L450. What is the likelihood that fishing quotas are going to be enforced in such areas? Is this a realistic strategy?

R: Yes, it is a realistic strategy since fishing is practiced in a relatively small partially protected MPA. Also, this MPA has financial and logistic resources to implement a collaborative co-management framework involving stakeholders and being informed by the small-scale fishing monitoring conducted by the state fishery agency. We clarified in the text (lines 480 - 485).

L469-471. This argument is counter-intuitive. Your manuscript is about using LEK to show the exploitation status of fisheries resources. And your recommendation now is to educate those fishers because they do not know about the status of their resources.

R: We agree with your suggestion and have rewritten this sentence. (Lines 507 - 512).

Useful reference missing in the ms:

Silvano, R.A.M. and Begossi, A., 2010. What can be learned from fishers? An integrated survey of fishers' local ecological knowledge and bluefish (Pomatomus saltatrix) biology on the Brazilian coast. Hydrobiologia, 637(1), p.3.

R: We added this reference in the introduction section.

Reviewer #3: Fogliarini et al., is an interesting ms dealing with the fishing down the food web process inferred from local ecological knowledge and landing data. The ms is pleasant and the ideas are organized and written with high quality. I have included a series of comments throughout the ms that I would like to see addressed before publication.

One important concern is regarding the different time scale from LEK data and from landing data. Interviews were performed from 2018 to 2019 and fishing land data from 1992 to 2008. there is at least a decade temporal difference on this data. Authors are confident that this do not imply results bias on the present study? I would like to see the development of this controversial time different in the ms discussion.

Results are well-presented and consistent. However, figures are very low quality and must be redone. See comments on the text. Fig 1 (map) is extremely low quality and unsuitable for publication.

Discussion is organized but lack of details in some important information. Comments are in the attached PDF.

I would be keen to review the ms once again before publication.

R. We thank Reviewer #3 for his/her comments and suggestions. We have tried to include all of the proposed alterations in this new MS version. We understand there is a time lag from landing data and the time when interviews were conducted but we are also certain that this does not affect our MS main conclusions. If the current time difference already reveals concordant patterns between landings trends and species mentioned as overexploited (declining trends) and new target species (increasing), we believe that if a longer landings data-series was available, that would make the patterns even clearer. If fishers were interviewed back in 2008, they would have possibly given the same answers on the current new target species and overexploited ones (see Bender et al. 2014 for overexploited taxa). We tried to have access to a longer landing data-series but did not receive any answers from local authorities that are responsible for fisheries monitoring. Data exists but won't be made available. We must also point out that targeted species have remained the same for the last 20 years (CEL Ferreira).

Major comments reviewer #3:

L46. ecological conclusion?? It is not a methodological paper...

The study adds extremely relevant information on the overexploitation status of many different species…etc.

R: We agree and modified this sentence into: "Our work reveals a depletion scenario of fishing stocks and changes in composition of fish species caught in the Arraial do Cabo."

L55. Fishing is not only an impact. Several fishing communities live from those resources and cope with the human X ocean relationship. Important to point out that unregulated and predatory fishing is the problem addressed herein.

R: Thank you for your comment. We agreed and modified this sentence. See line 54.

L62. Briefly mention the consequences please.

R: Among the consequences of unsustainable removal of top predators are the release of prey. In other words, the loss of top predators can cause numerical increases in meso-consumers and alter community structure. We mention other consequences in the following paragraph. Please see line 65.

L79."....have been proposed so far and our study aims to add information on..."
Complete your sentence as suggested so it would be more useful.

R: This sentence was rewritten (Lines 79 - 84).

L126. Poor figure quality. Difficult to identify where is study area in Brazilian coast.
I would like to suggest a new map with better quality.

R: We adjust the quality of figures.

L132. Not mentioned in the Fig where is the MPA.

R: The figure 1 was substitute by an adapted map due copyright for publication, in this new version there is no mention of the MPA.

L165. This seems like a discussion. Any reference for this idea?

R: We added the reference Mendonça et al. (2013).

L175. Interviews were acquired from 2018 to 2019 and fishing land data from 1992 to 2008. there is at least a decade temporal difference on this data. Authors are confident that this do not imply results bias on the present study? I would like to see the development of this controversial time different in the ms discussion.

R: Please see answer above (L180). We have also included that in the discussion.

L176. In the reference list? How this data was acquired?

R: It is not in the list of references because these data were acquired directly from the Fundação de Pesca de Arraial do Cabo - FIPAC. These is the first fisheries monitoring reports in Arraial do Cabo, and are available on the FIPAC website (see https://www.fipac.rj.gov.br).

L180. Again. As previously mentioned, this is a quite old data. Fishing data from 1992 (almost 30 years old) represents the MTL current scenario? Probably trophic levels are even worst nowadays. This must be discussed.

R: We agree with reviewer's comment that there are temporal differences in the data sources. Fisheries statistics were obtained through Fundação Instituto da Pesca de Arraial do Cabo (FIPAC) which shared only a short monitoring time series (1992 – 2008). We agree that the current scenario for trophic levels can be even worse, as our results showed declines in high-trophic species.

L346. This result came out of nowhere. Please, describe in the aims, methods etc.

R: We describe this result in all sections of our manuscript. Please see how we described in each section:

Introduction (Objectives): "We also discuss the main causes of fish overexploitation and management strategies in a subtropical fisheries hotspot."

Materials and methods (Data collection): "(iii) what are the main causes of species' overexploitation".

Results: "Fishers mentioned 35 different possible causes of fisheries overexploitation in Arraial do Cabo (1.58 ± 1.08, mean ± standard deviation). The five most mentioned causes were the increased fishing effort (22%), which is recognized by fishers as an increase in the number of fishers and boats; presence of industrial fishing vessels (21%), presence of purse seine fisheries (19%), gillnet fishing (16%), and trawling (12%)."

In the following paragraph of the discussion: "The cause more mentioned by fishers as responsible for fisheries overexploitation was increase in the fishing effort…"

L366. where are the ecological implications? this paragraph needs to end up with a broad view of your main results.

R: We agree with your suggestion and incorporated more explanations in this paragraph. Please see lines 396-400.

L465. how small? Is a no-take zone implementation process been currently discussed with local community?

R: We correct the term "small MPAs" to "small no-take areas". The implementation process had been discussed with the local community recently. In 2021 a management plan with the contribution of researchers, community members, and fishers, will suggest the implementation of 3 to 4 small no-take areas within the extractive marine reserve. It is a participatory process, which will establish specific rules for the use of each of these areas.

L465. how short? More info is necessary.

R: We add more explanation in lines 497 - 500.

L480. I would like to see more discussion on the conservation strategies along the ms discussion.

R: We added more details on the management strategies, following the reviewers' suggestion. Now, we describe, minimum landing size of capture, closure periods how fishing quotas can be implemented in a participatory manner the importance of no-take MPAs, a improve of surveillance for banning the illegal fisheries and the importance of young generations acknowledging the past abundance of marine

resources to be more willing to accept management and conservation initiatives (lines 478 - 504).

References

Alleway HK, Connell SD, Ward TM, & Gillanders BM. (2014). Historical changes in mean trophic level of southern Australian fisheries. Marine and Freshwater Research, 65(10), 884-893.

Bender MG, Machado GR, de Azevedo Silva PJ, Floeter SR, Monteiro-Netto C, Luiz OJ, & Ferreira CE. (2014). Local ecological knowledge and scientific data reveal overexploitation by multigear artisanal fisheries in the Southwestern Atlantic. PLoS One, 9(10), e110332.

Blanchard JL, Coll M, Trenkel VM, Vergnon R, Yemane D, Jouffre D, et al. (2010). Trend analysis of indicators: a comparison of recent changes in the status of marine ecosystems around the world. ICES J Mar Sci. 67 (4): 732-744.

Branch et al. (2010). The trophic fingerprint of marine fisheries. Nature 468, 431–435.

Branch TA. (2015). Fishing Impacts on Food Webs: Multiple Working Hypotheses. 576 Fisheries. 40 (8): 373-375.

Coll M, Shannon LJ, Yemane D, Link JS, Ojaveer H, Neira S, et al. (2010). Ranking the ecological relative status of exploited marine ecosystems. ICES J Mar Sci. 67 (4): 769-786.

Da Silva, L. D. M. C., Machado, I. C., dos Santos Tutui, S. L., & Tomás, A. R. G. (2020). Local ecological knowledge (LEK) concerning snook fishers on estuarine waters: Insights into scientific knowledge and fisheries management. Ocean & Coastal Management, 186, 105088.

FAO. 2020. The State of World Fisheries and Aquaculture 2020. Sustainability in action. Rome. https://doi.org/10.4060/ca9229en

Ferreira, H. M., Reuss-Strenzel, G. M., Alves, J. A., & Schiavetti, A. (2014). Local ecological knowledge of the artisanal fishers on *Epinephelus itajara* (Lichtenstein, 1822) (Teleostei: Epinephelidae) on Ilhéus coast–Bahia State, Brazil. Journal of ethnobiology and ethnomedicine, 10(1), 51.

Herbst, D. F., & Hanazaki, N. (2014). Local ecological knowledge of fishers about the life cycle and temporal patterns in the migration of mullet (*Mugil liza*) in Southern Brazil. Neotropical Ichthyology, 12(4), 879-890.

Kirchherr, J., Charles, K. (2018). Enhancing the sample diversity of snowball samples: Recommendations from a research projecton anti-dam movement in Southeast Asia. Plos ONE 13(8): e02017

McClenachan, L. (2009). Historical declines of goliath grouper populations in South Florida, USA. Endangered Species Research, 7(3), 175-181.

Sethi et al. (2010). Global fishery development patterns are driven by profit but not trophic level. PNAS 107, 12163-12167.

Silvano, R. A. M., & Begossi, A. (2010). What can be learned from fishers? An integrated survey of fishers’ local ecological knowledge and bluefish (*Pomatomus saltatrix*) biology on the Brazilian coast. Hydrobiologia, 637(1), 3.
